# Identification and Expression Analyses of Atypical NLR Genes in Potato

**DOI:** 10.3390/plants15213325

**Published:** 2026-10-27

**Authors:** Bao Hai, Niu Niu, Wenjing Bai, Wei Shao

**Affiliations:** 1 Key Laboratory of Herbage & Endemic Crop Biology, Ministry of Education, School of Life Sciences, Inner Mongolia University, Hohhot 010070, Chinaniuniu1901@126.com (N.N.); 22608034@mail.imu.edu.cn (W.B.); 2 School of Life Science and Technology, Inner Mongolia University of Science & Technology, Baotou 014010, China

**Keywords:** potato, NLR, integrated domain, stress and defense, C-JID, expression pattern

## Abstract

Defense responses against pathogens are essential for plant growth, and the nucleotide-binding leucine-rich repeat receptor (NLR) family is an important component of plant immunity. Besides NBS and LRR domains, NLR proteins also contain TIR, CC, or RPW8 domain at their N-terminus and the functions of these canonical domains are well documented. Recently, some atypical X domains, also named integrated domains (IDs), have been reported to be encoded by NLR genes and to play key roles in effector-triggered immunity. Here, we screened the plant NLR database to identify atypical NLRs in potato. A total of 181 atypical NLR protein entries, identified from 642 candidates as truncated or St-NLR-IDs (potato NLRs encoding ID) and comprising 147 NLR-IDs and 34 truncated NLRs, were classified into four major groups, including T(TIR)-class, C(CC)-class, N(NBS)-class, and X-class, and together with 12 typical CNL/TNL sequences, they were used for phylogenetic analysis. Promoter regions of the majority of these entries contain “stress and defense” elements such as MYB/MYC *cis*-regulatory element, indicating their involvement in plant immune response. In the Rywal background, the expression of six genes was upregulated and that of nineteen genes was downregulated. In the NahG-Rywal plants, the expression of seventeen genes was upregulated and that of eight genes was downregulated. In the shRBOHD-Rywal background, the expression of eighteen genes was upregulated and that of six genes was downregulated. We also investigated the expression of four C-JID-containing NLR-ID genes, *M1BEE2*, *M1BF53*, *M1A5Z0*, and *M1BZB1*, upon PVX, PVY infection and SA treatment by RT-qPCR and suggested their potential involvement in plant immunity. Promoter–GUS assays for the above four genes also supported their responses to viral infection and SA treatment. In summary, we identified and classified atypical NLRs in potato, analyzed their expression under PVY^N-Wilga^ infection using public transcriptome data, and validated the virus- and SA-responsive expression of four C-JID-encoding genes, providing a resource for future functional studies.

## 1. Introduction

In nature, plants face many environmental challenges, including abiotic and biotic stresses, which are characterized by viruses, bacteria, fungi, nematodes, and herbivorous insects. Plants have evolved complex immune receptors such as pattern-recognition receptors (PRRs) to specifically perceive pathogenic factors known as microbe-associated molecular patterns (MAMPs) and pathogen-associated molecular patterns (PAMPs), leading to the activation of pattern-triggered immunity (PTI). In addition, plants also trigger effector-triggered immunity (ETI), which is often accompanied by a stronger and more robust response characterized by local cell death and hypersensitive response (HR) [1,2,3]. ETI is often initiated by the activity of resistance (R) proteins, which typically contain two conserved domains, the nucleotide-binding site (NBS) domain and the leucine-rich repeat (LRR) domain; these are collectively known as NLR proteins. The NLR family in plants belongs to the STAND (signal transduction ATPases with numerous domains) AAA+ superfamily. In addition to NBS and LRR domains, NLR proteins also possess several characteristic N-terminal domains such as Toll/interleukin-1 receptor (TIR), coiled coil (CC), and resistance to powdery mildew 8 (RPW8)-like domains [4]. The functions of these typical domains, such as NBS, LRR, TIR, CC, and RPW8, are well characterized in plant immunity, and the downstream immune signaling landscape has also been uncovered.

Besides these typical domains, an increasing number of NLR proteins have been shown to contain noncanonical, so-called integrated domains (IDs) that display polymorphic distributions. The type, number, and position of IDs in NLR proteins are varied according to the specific R protein and most of them may function as regulatory elements, cofactors, or baits, acting by themselves or participating in the “integrated decoy” mechanism [5], as exemplified in the *RPS4/RRS1* gene pair [6] in *Arabidopsis thaliana* and the *RGA4/RGA5* gene pair [7] in rice. For example, RRS1 contains an atypical WRKY domain that recognizes the pathogen effector PopP2, an acetyltransferase from *Ralstonia solanacearum*, and AvrRps4 from *Pseudomonas syringae pv. pisi*, and cooperates with its partner RPS4 to mediate immune response [8,9]. In the *RGA4/RGA5* gene pair, RGA5 possesses the atypical HMA domain that recognizes the effectors such as AVR-Pia and AVR1-CO39 from the rice blast fungus *Magnaporthe oryzae* and modulates *RGA4*-mediated immune signaling [10,11]. Several IDs are reported to function in TNL-type R pairs such as *Arabidopsis RRS1B/RPS4B* [12] and *CHS3/CSA1* [13], while others are identified in CNL-type R pairs such as rice *Pik-1/Pik-2* alleles [14] and rapeseed *BnRPR1/BnRPR2* [15].

In addition to participating in R pairs, some IDs have also been shown to function independently, either by recognizing pathogen effectors or by collaborating with other signaling partners. For example, soybean GmNLR-ID#85 possesses a C-terminal integrated WRKY domain that binds to the type III effector PopP2 from *Ralstonia pseudosolanacearum* [16]. The wheat R proteins Yr5, Yr7, and YrSP contain an N-terminal BED domain that mediates downstream signaling [17]. The tomato protein Sw-5b possesses an N-terminal SD domain that mediates resistance to tomato spotted wilt virus [18]. The wheat resistance protein YrU1 contains both the N-terminal ankyrin repeat domain and the C-terminal WRKY domain, which participate in immune response toward stripe rust [19]. The existence of IDs in NLR proteins largely broadens the recognition spectra and immune repertoire of plants, thereby necessitating their identification and functional analyses in plant immunity. In recent years, with the in-depth study of NLR proteins and the rapid development of bioinformatics technology, researchers have identified the high-frequency occurrence of IDs in over 40 plant species [20,21,22]. In a genome-wide survey of soybean (*Glycine max*(L.) Merr.), Shao et al. identified 210 *TNL-like* genes encompassing 36 integrated domains. In addition, multiple soybean mosaic virus (SMV) resistance genes were characterized, and several SMV effectors showing interaction with different integrated domains were selected [23].

Potato (*Solanum tuberosum* L.) is the third-largest food crop in the world (after rice and wheat), serving as a strategic crop for ensuring global food security, supporting population growth, and promoting agricultural economies, thanks to its three core advantages: high and stable yield, comprehensive nutrition, and strong adaptability. Potato NLR genes have been extensively investigated, with many canonical NLRs such as *Rx* against PVX and the *R3a/Rpi* family against *Phytophthora infestans* being functionally characterized [24,25]. Recent genome-wide surveys have further revealed the widespread presence of NLRs harboring noncanonical integrated domains (IDs) in the potato NLRome [26]. Notably, the HMA domain has been identified as a functional integrated domain that enables effector perception, as exemplified by *Rpi-brk1*, which recognizes a *P. infestans* effector through its HMA domain [26]. Despite these advances, a systematic classification of atypical NLRs in potato, particularly regarding their expression profiles upon pathogen challenge and hormonal signaling, remains lacking. In this study, the potato genome was comprehensively screened using an online database (https://nlrscape.biochim.ro), yielding 642 NLR protein entries, among which 181 were identified as atypical NLR protein entries, comprising 147 NLR-IDs and 34 truncated NLRs. Based on their domain architectures, these genes were grouped into TIR-, CC-, NBS-, and X-classes. Through detailed transcriptional profiling under biotic stress conditions combined with promoter–GUS assays, we further suggested the involvement of selected candidates in responses to viral infection and salicylic acid signaling. Our findings provide a foundational repertoire of atypical NLR protein entries in potato and offer new insights into their potential roles in effector-triggered immunity, thereby expanding the current understanding of NLR-mediated defense mechanisms in Solanaceous crops.

## 2. Results

### 2.1. Identification of Potato Genes Encoding Atypical NLR Proteins

While nucleotide-binding leucine-rich repeat domains are canonical components of resistance (R) proteins, some atypical domains known as integrated domains (IDs) are fused to form NLR-ID proteins. An increasing number of NLR-ID proteins with noncanonical domains have been reported in several plant species to play important roles in immune signaling [5]. In order to gain insight into the function of these proteins, we screened atypical NLRs in *Solanum tuberosum*, a major crop threatened by a variety of pathogens. In total, 642 NLR protein entries were retrieved from the NLRscape database (https://nlrscape.biochim.ro), an atlas of plant NLRs (Supplementary Table S1). NLRscape curates sequences containing at least one NBS-like domain, so all 642 entries were considered NLR-related by the database. These sequences were also verified in the SpudDB potato genome database DM v6.1 via https://spuddb.uga.edu/ using BLAST searches. Of the 642 NLR protein entries, 44 lacked an assignable PGSC gene ID and were designated as “Unknown”; the remaining 598 entries were assigned to 427 unique PGSC gene IDs. Thus, the 642 protein entries represented 427 uniquely identified gene loci, with multiple protein entries sometimes corresponding to the same gene locus (Supplementary Table S2). As shown in Supplementary Figure S1, these NLRs are distributed on twelve chromosomes in potato in a clustered manner and are more abundant on chromosomes four and eleven.

**Figure 1:**
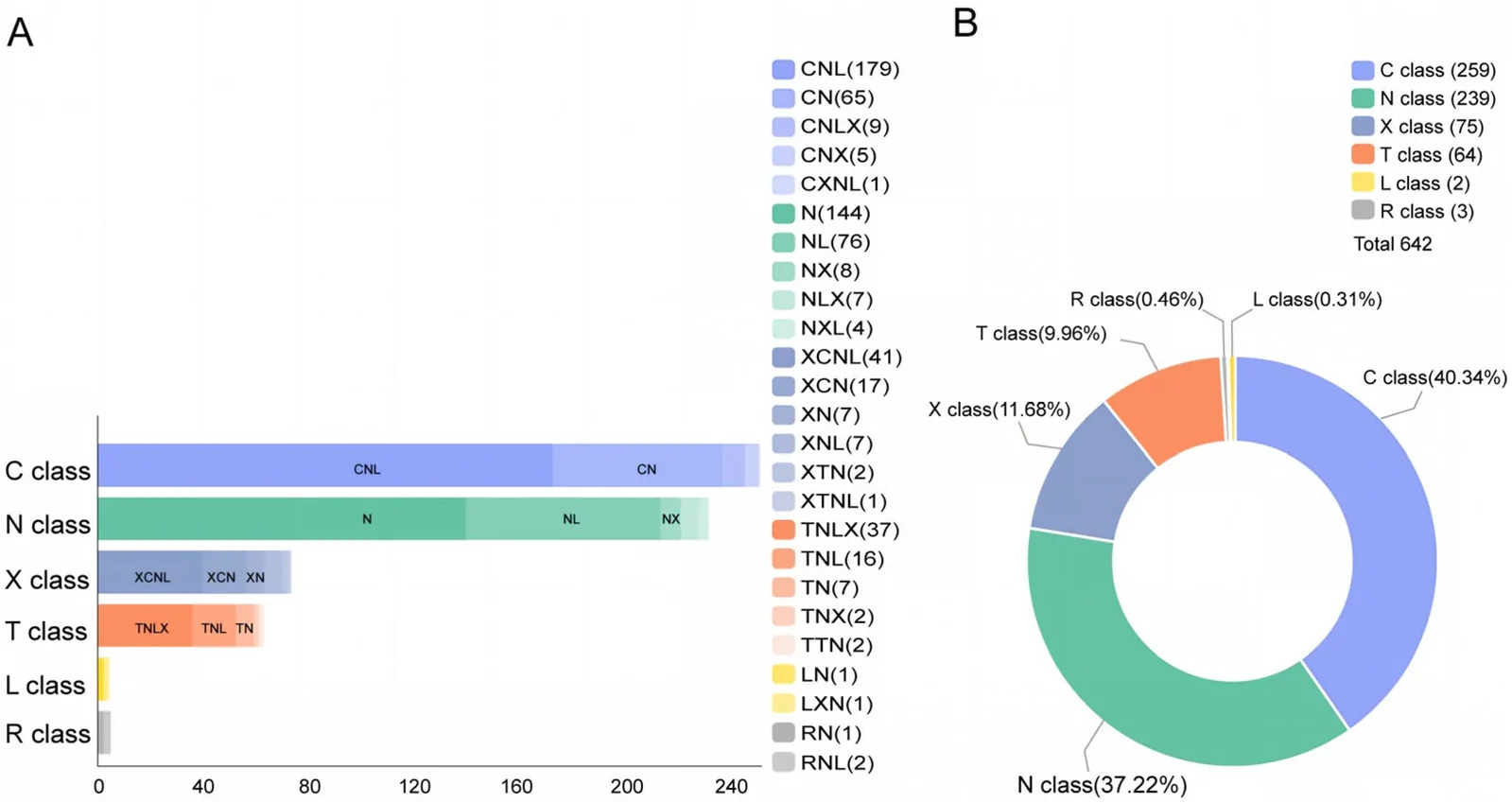
Classification and subtype composition of 642 potato NLR protein entries. (**A**) Distribution of NLR subtypes within the six major classes, C, N, X, T, L, and R. Horizontal stacked bars show the number and composition of individual subtypes within each class. (**B**) Overall distribution of the 642 NLR protein entries among the six major classes. Sector sizes indicate the relative proportion of each class, and numbers in the legend indicate the corresponding number of protein entries.

As NLRs are mainly classified into three types, TNL, CNL, and RNL, the potato NLRs were also grouped into six major groups (CC, NBS, X, TIR, LRR, RPW8) and further into 25 subgroups according to the appearance, composition, and position of typical domains CC, TIR, RPW8, NBS, LRR and atypical IDs (Figure 1). These domains were also annotated and verified in Pfam (http://pfam-legacy.xfam.org/) (accessed on 1 August 2026) and SMART (http://smart.embl-heidelberg.de/) (accessed on 1 August 2026) databases.

As we are interested in atypical NLRs, proteins with truncated domains and atypical IDs were further analyzed. This screening identified a total of 193 NLR-related protein entries, comprising 181 atypical NLRs (including 147 NLR-IDs and 34 truncated NLRs) and 12 typical CNL/TNL protein entries (only as reference sequences for phylogenetic analysis) (Supplementary Table S3). Of the 193 selected protein entries, 191 mapped to 161 unique PGSC gene loci, while the remaining two entries (Q93YA7 and Q93YA6) lacked an identified PGSC gene ID. All downstream classification, phylogenetic and expression analyses were performed at the protein-entry level. After careful sequence alignment and comparison, we classified the atypical NLRs in potato into four categories, C-class with CC domain, N-class with NBS domain, X-class with ID, and T-class with TIR domain at their N-termini, respectively (Supplementary Figure S2). Figure 1 presents the overall classification of all 642 NLR protein entries, whereas Supplementary Figure S2 specifically shows the subtype composition of the selected entries used for downstream analyses.

### 2.2. Analysis of C-Class Atypical NLRs

**Figure 2:**
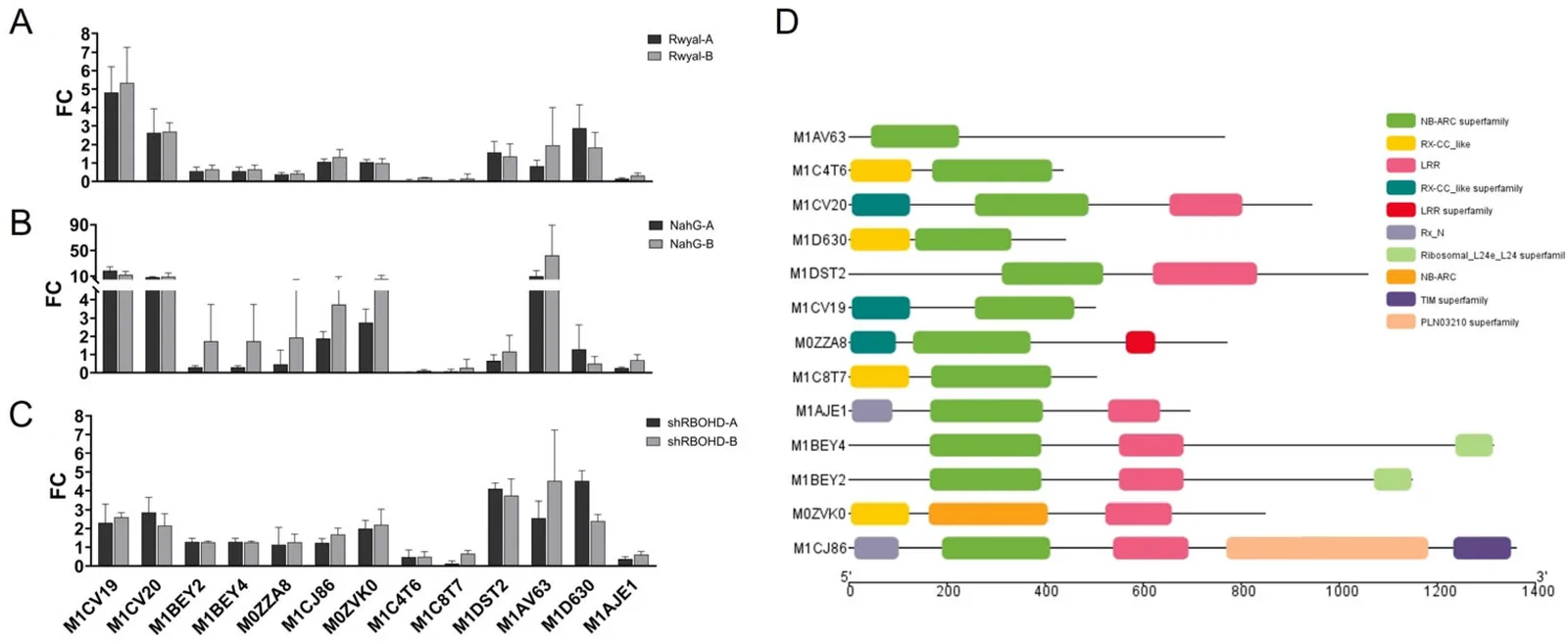
Expression patterns and integrated domain prediction of selected C-class atypical NLRs with altered expression after PVY^N-Wilga^ infection in potato. (**A**–**C**) Transcript levels of selected C-class atypical NLRs in Rywal (**A**), NahG-Rywal (**B**), and shRBOHD-Rywal (**C**) plants. Representative genes with altered expression (FC ≥ 2 or ≤0.5) in at least one of the three backgrounds are shown. Transcripts were analyzed in the cell death region and surrounding tissue using public transcriptome data. The *x*-axis represents UniProt accession numbers of the selected C-class atypical NLRs; the *y*-axis represents fold change (FC). Control values were normalized to 1 and are not shown. Error bars represent the standard deviation among three biological replicates. (**D**) Schematic of predicted integrated domains in the selected C-class atypical NLRs. The *x*-axis represents amino acid sequence length; labels on the right indicate predicted domains. Predicted domains include Rx_N, ribosomal_L24e, TIM, and PLN03210.

C-class atypical NLRs include CNL, CN, CNLX, CNX, and CXNL subgroup protein entries. The promoter regions of C-class atypical NLRs contain highly represented “stress and defense” elements, among which MYB/MYC elements are most enriched (Supplementary Figure S3). Phylogenetic analysis of these C-class atypical NLRs is presented in Supplementary Figure S3, which showed that they formed distinct clades largely consistent with their domain architecture-based subtypes. In order to understand the putative involvement of these genes in pathogen response, we analyzed their expression under the following three conditions by using public transcriptome data (NCBI Gene Expression Omnibus (GEO) database, accession no. GSE142002). Rywal exhibits a hypersensitive response (HR) upon virus infection, which limits the virus to the entry site. Under resistance (Rywal) conditions, expression of *M1CV19* and *M1CV20* was elevated, while that of *M0ZZA8*, *M1C4T6*, *M1C8T7*, and *M1AJE1* was decreased (Figure 2A). NahG-Rywal plants, which have compromised salicylic acid accumulation, develop necrotic lesions that fail to limit viral spread and exhibit a delayed defense response. In NahG-Rywal plants, expression of *M1CV19*, *M1CV20*, *M0ZVK0*, and *M1AV63* was upregulated, while that of *M1C4T6* and *M1C8T7* was downregulated (Figure 2B). The shRBOHD-Rywal plant, in which the *RBOHD* gene is silenced, is unable to arrest the spread of the virus. In shRBOHD-Rywal plants, expression of *M1CV19*, *M1CV20*, *M1DST2*, *M1AV63*, and *M1D630* was upregulated, while that of *M1C4T6* was downregulated (Figure 2C). These protein entries contain integrated domains such as Rx_N, the Ribosomal_L24e superfamily, the TIM superfamily, and the PLN03210 superfamily (Figure 2D).

### 2.3. Analysis of N-Class Atypical NLRs

**Figure 3:**
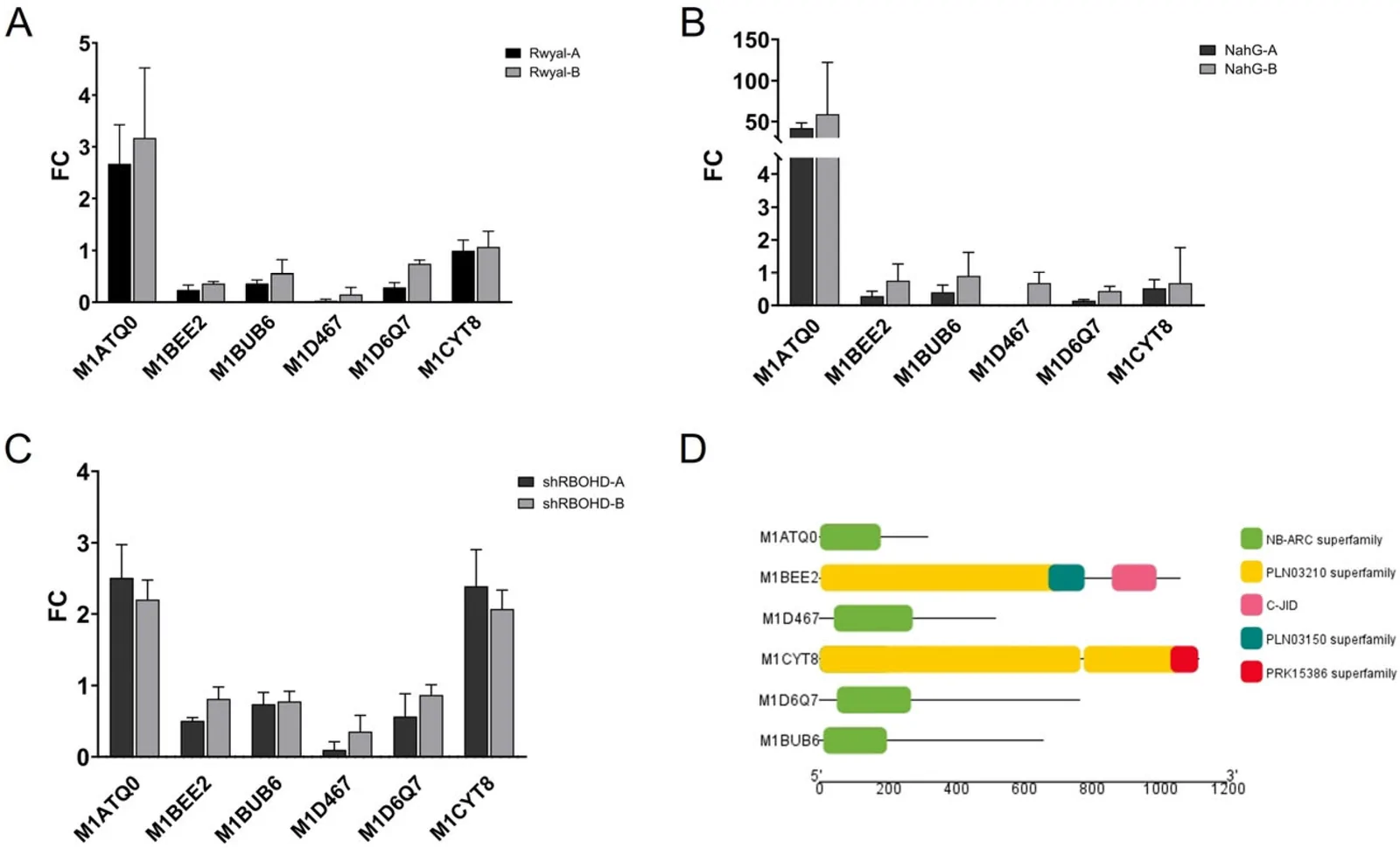
Expression patterns and integrated domain prediction of selected N-class atypical NLRs with altered expression after PVY^N-Wilga^ infection in potato. (**A**–**C**) Transcript levels of selected N-class atypical NLRs in Rywal (**A**), NahG-Rywal (**B**), and shRBOHD-Rywal (**C**) plants. Representative genes with altered expression (FC ≥ 2 or ≤0.5) in at least one of the three backgrounds are shown. Transcripts were analyzed in the cell death region and surrounding tissue using public transcriptome data. The *x*-axis represents UniProt accession numbers of the selected N-class atypical NLRs; the *y*-axis represents fold change (FC). Control values were normalized to 1 and are not shown. Error bars represent the standard deviation among three biological replicates. (**D**) Schematic of predicted integrated domains in the selected N-class atypical NLRs. The *x*-axis represents amino acid sequence length; labels on the right indicate predicted domains. Predicted domains include PLN03210, C-JID, PLN03150, and PRK15386.

N-class atypical NLRs include NX, NL, N, NLX, and NXL subgroup protein entries. The promoter region of N-class atypical NLRs contains highly represented “stress and defense” elements, among which MYB/MYC elements are most richly present (Supplementary Figure S4). Phylogenetic analysis of these N-class atypical NLRs is presented in Supplementary Figure S4, which similarly revealed clades consistent with their domain architecture-based subtypes. In Rywal plants, the expression of *M1ATQ0* was elevated, while that of *M1BEE2* and *M1D467* was decreased (Figure 3A). In NahG-Rywal plants, expression of *M1ATQ0* was upregulated, while that of *M1D6Q7* was downregulated (Figure 3B). In shRBOHD-Rywal plants, expression of *M1ATQ0* and *M1CYT8* was upregulated, while that of *M1D467* was downregulated (Figure 3C). These protein entries contain integrated domains such as the PLN03210 superfamily, C-JID (C-terminal jelly-roll and Ig-like domain), the PLN03150 superfamily, and the PRK15386 superfamily (Figure 3D).

### 2.4. Analysis of T-Class Atypical NLRs

**Figure 4:**
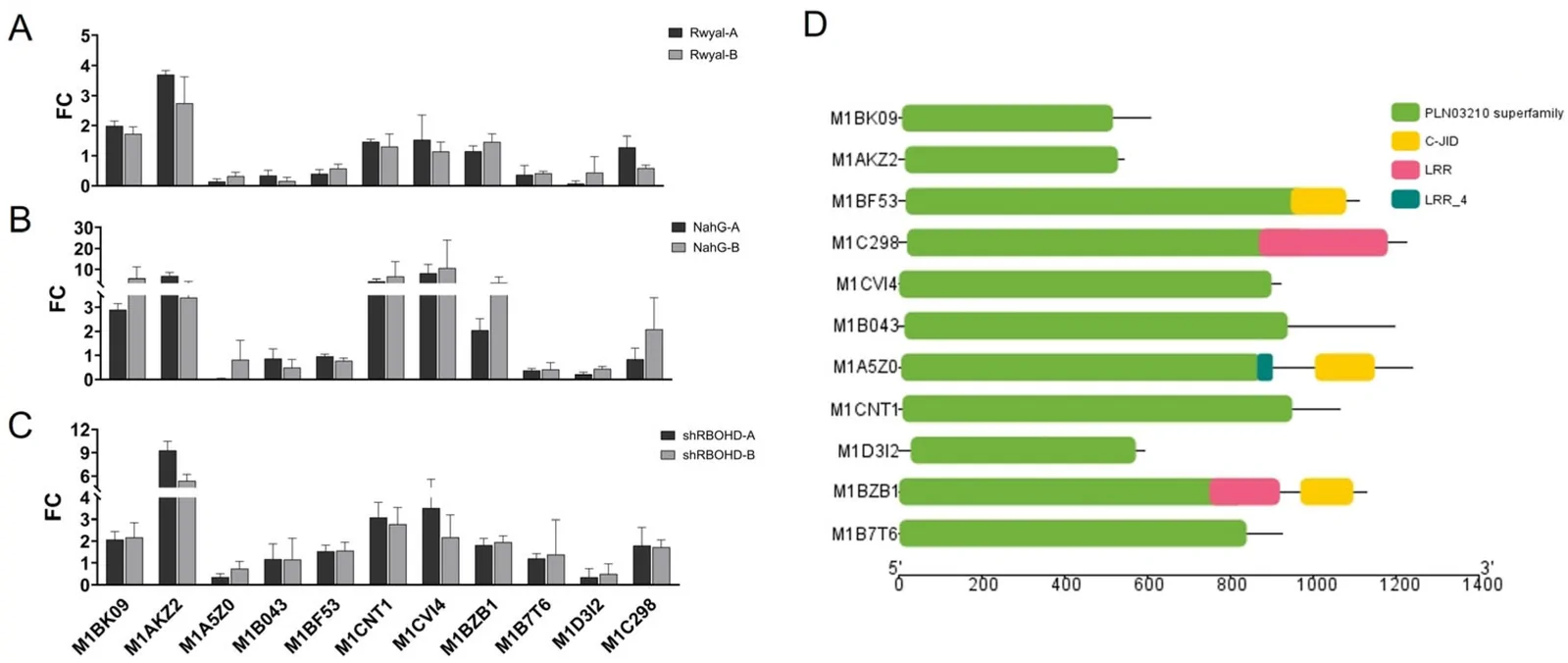
Expression patterns and integrated domain prediction of selected T-class atypical NLRs with altered expression after PVY^N-Wilga^ infection in potato. (**A**–**C**) Transcript levels of selected T-class atypical NLRs in Rywal (**A**), NahG-Rywal (**B**), and shRBOHD-Rywal (**C**) plants. Representative genes with altered expression (FC ≥ 2 or ≤0.5) in at least one of the three backgrounds are shown. Transcripts were analyzed in the cell death region and surrounding tissue using public transcriptome data. The *x*-axis represents UniProt accession numbers of the selected T-class atypical NLRs; the *y*-axis represents fold change (FC). Control values were normalized to 1 and are not shown. Error bars represent the standard deviation among three biological replicates. (**D**) Schematic of predicted integrated domains in the selected T-class atypical NLRs. The *x*-axis represents amino acid sequence length; labels on the right indicate predicted domains. Predicted domains include PLN03210 and C-JID.

T-class atypical NLRs include TNLX, TNX, TN, and TNL subgroup protein entries. The promoter region of T-class atypical NLRs contains highly represented “stress and defense” elements, among which MYB/MYC elements are most richly present (Supplementary Figure S5). Phylogenetic analysis of these T-class atypical NLRs is presented in Supplementary Figure S5, which showed clade structures consistent with their domain architecture-based subtypes. In Rywal plants, the expression of *M1AKZ2* was elevated, while that of *M1A5Z0*, *M1B043*, *M1B7T6*, and *M1D3I2* was decreased (Figure 4A). In NahG-Rywal plants, expression of *M1BK09*, *M1AKZ2*, *M1CNT1*, *M1CVI4*, and *M1BZB1* was upregulated, while that of *M1B7T6* and *M1D3I2* was downregulated (Figure 4B). In shRBOHD-Rywal plants, expression of *M1BK09*, *M1AKZ2*, *M1CNT1*, and *M1CVI4* was upregulated, while that of *M1D3I2* was downregulated (Figure 4C). These protein entries contain integrated domains such as the PLN03210 superfamily and C-JID (Figure 4D).

### 2.5. Analysis of X-Class Atypical NLRs

**Figure 5:**
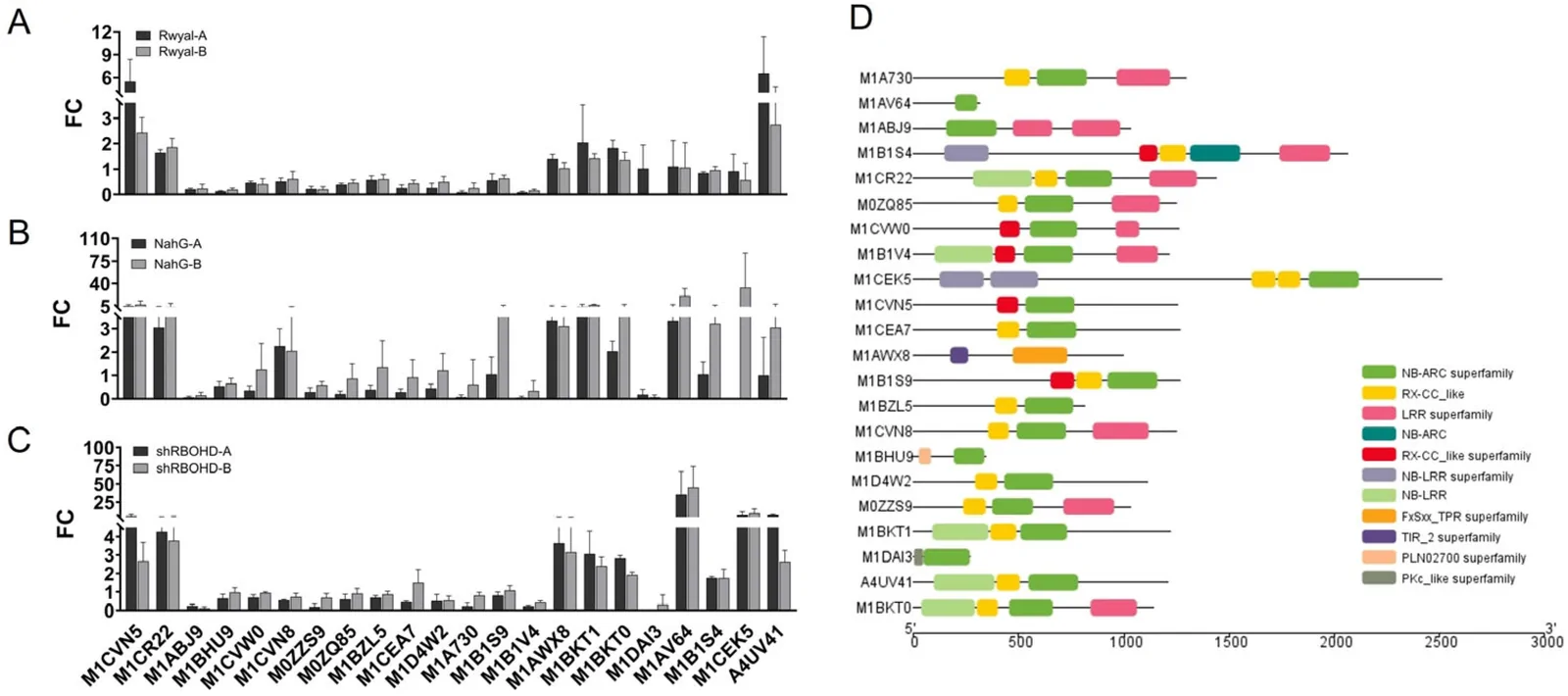
Expression patterns and integrated domain prediction of selected X-class atypical NLRs with altered expression after PVY^N-Wilga^ infection in potato. (**A**–**C**) Transcript levels of selected X-class atypical NLRs in Rywal (**A**), NahG-Rywal (**B**), and shRBOHD-Rywal (**C**) plants. Representative genes with altered expression (FC ≥ 2 or ≤0.5) in at least one of the three backgrounds are shown. Transcripts were analyzed in the cell death region and surrounding tissue using public transcriptome data. The *x*-axis represents UniProt accession numbers of the selected X-class atypical NLRs; the *y*-axis represents fold change (FC). Control values were normalized to 1 and are not shown. Error bars represent the standard deviation among three biological replicates. (**D**) Schematic of predicted integrated domains in the selected X-class atypical NLRs. The *x*-axis represents amino acid sequence length; labels on the right indicate predicted domains. Predicted domains include FxSxx_TPR, PLN02700, and PKc_like.

X-class atypical NLRs include XN, XTN, XNL, XTNL, XCNL, and XCN subgroup protein entries. The promoter region of X-class atypical NLRs contains highly represented “stress and defense” elements, among which MYB/MYC elements are most richly present (Supplementary Figure S6). Phylogenetic analysis of these X-class atypical NLRs is presented in Supplementary Figure S6, which revealed distinct clades consistent with their domain architecture-based subtypes. In Rywal plants, expression of *M1CVN5* and *A4UV41* was elevated, while that of *M1ABJ9*, *M1BHU9*, *M1CVW0*, *M0ZZS9*, *M0ZQ85*, *M1CEA7*, *M1D4W2*, *M1A730*, and *M1B1V4* was decreased (Figure 5A). In NahG-Rywal plants, expression of *M1CVN5*, *M1CR22*, *M1CVN8*, *M1AWX8*, *M1BKT1*, *M1BKT0*, and *M1AV64* was upregulated, while that of *M1ABJ9*, *M1B1V4*, and *M1DAI3* was downregulated (Figure 5B). In shRBOHD-Rywal plants, expression of *M1CVN5*, *M1CR22*, *M1AWX8*, *M1BKT1*, *M1AV64*, *M1CEK5*, and *A4UV41* was upregulated, while that of *M1ABJ9*, *M1B1V4*, and *M1DAI3* was downregulated (Figure 5C). These protein entries contain integrated domains such as the FxSxx_TPR superfamily, the PLN02700 superfamily, and the PKc_like superfamily (Figure 5D).

### 2.6. Expression Analysis of Potato C-JID Encoding Genes upon Viral Infection and SA Treatment

**Figure 6:**
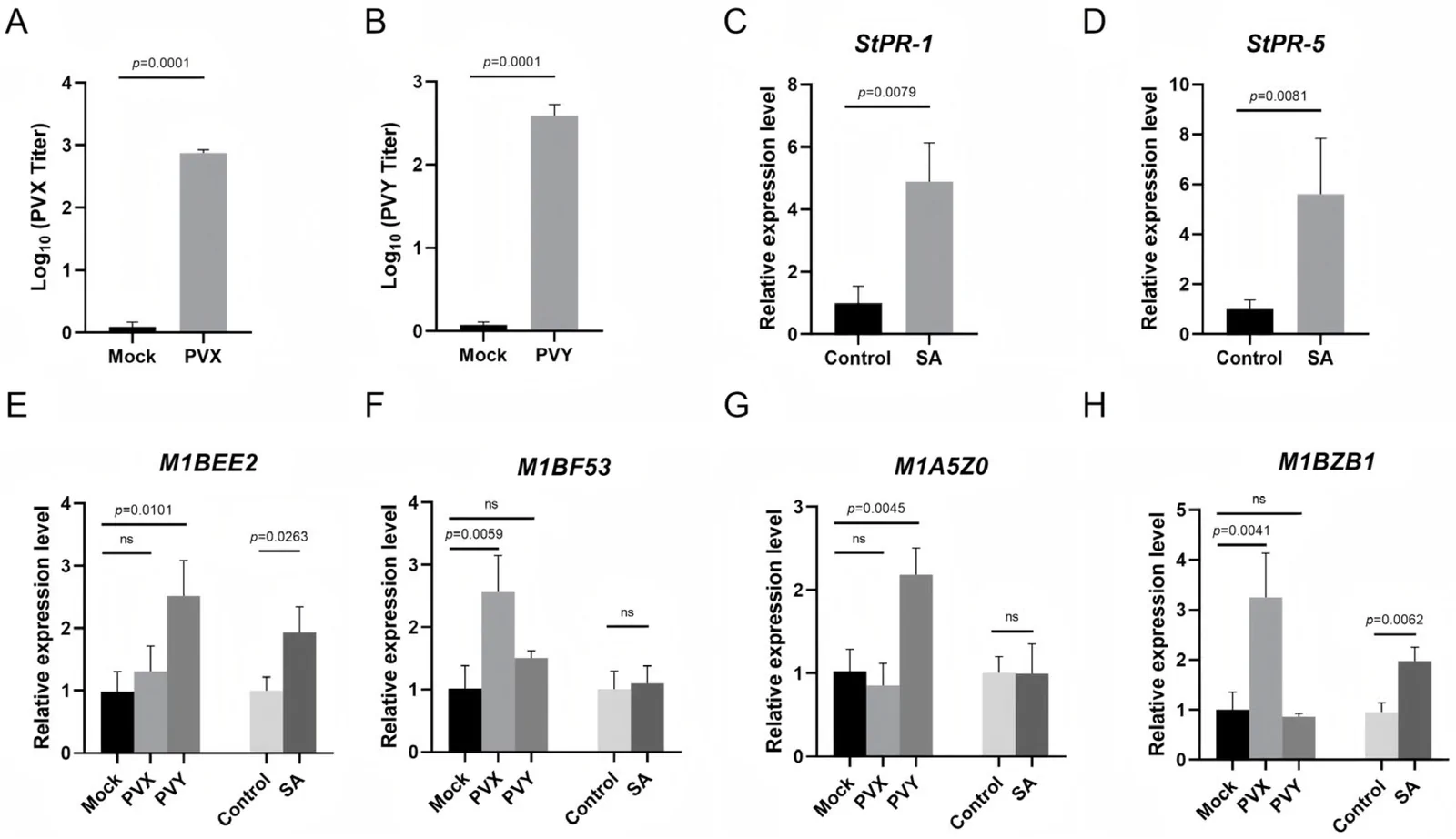
Relative expression of potato resistance-related genes in response to PVX, PVY and SA in *Solanum tuberosum*. Leaf tissues of potato were harvested at 7 days post-inoculation (dpi) with PVY and PVX, or subjected to SA treatment. For the analysis, 10 leaves were pooled to reduce leaf-to-leaf variation and ensure a representative sample. Total RNA was extracted from the pooled sample. Three independent biological replicates were performed. RT-qPCR was performed using gene-specific primers. The 2^−△△Ct^ method was used for calculation of target gene expression level normalized with internal control *StActin* mRNA level. Mock: Wild-type potato without virus inoculation. Control: Wild-type potato without SA treatment. (**A**,**B**) PVX and PVY virus titer detection. Potato leaves were inoculated with PVX or PVY, and viral accumulation was detected at 7 dpi by RT-qPCR. Virus titers are presented as log10-transformed values. The *y*-axis represents the mean log10-transformed virus titer in the leaf infiltration region and the *x*-axis represents the target gene. (**C**,**D**) Effects of SA treatment on defense-related gene expression in potato. Relative expression levels of the potato pathogenesis-related genes *StPR-1* and *StPR-5* were measured by RT-qPCR at 2 dpi. (**E**–**H**) Expression profiles of selected defense-related genes in potato in response to PVX, PVY and SA treatment. Potato leaves were inoculated with PVX or PVY, while additional groups received SA treatment. Transcript levels of *StM1BEE2* (**E**), *StM1BF53* (**F**), *StM1A5Z0* (**G**) and *StM1BZB1* (**H**) were quantified by RT-qPCR. Values are means ± SD from three independent biological replicates (*n* = 3), each with three technical replicates. Error bars represent the standard deviation among biological replicates. Exact *p* values are indicated in the figure. For the viral inoculation experiment (mock, PVX, and PVY), significant differences compared with mock were determined by one-way ANOVA followed by Dunnett’s post hoc test. For the SA treatment experiment (control and SA), significant differences were determined by Student’s *t*-test. *p* < 0.05 was considered statistically significant.

In order to further investigate the involvement of some potato NLR-ID genes in pathogen responses, we selected four genes encoding C-JID domains, *M1BEE2* (*PGSC0003DMG400016808*), *M1BF53* (*PGSC0003DMG402016981*), *M1A5Z0* (*PGSC0003DMG400006003*), and *M1BZB1* (*PGSC0003DMG400021887*) to analyze their expression (Supplementary Figure S7). These four genes were selected because they all contain a C-JID domain, show altered expression in the public transcriptome dataset (GSE142002) upon PVY^N-Wilga^ infection in at least one of the three genetic backgrounds, and represent different atypical NLR subclasses (N-class and T-class). In this regard, potato virus X (PVX) and potato virus Y (PVY) were selected as the pathogens as they are major viral diseases of potato. Salicylic acid (SA) is often regarded as the defense-related hormone in plant immunity. The standard potato variety Atlantic was infected by PVX, PVY, and treated with SA via leaf spraying. Total RNA was isolated from both mock and treated plants, and cDNA was synthesized. Successful viral infection was confirmed by viral titer assays using RT-qPCR (Figure 6A,B). SA treatment was also verified by the significant induction of potato marker genes such as *PR-1* and *PR-5* via RT-qPCR assays (Figure 6C,D).

**Figure 7:**
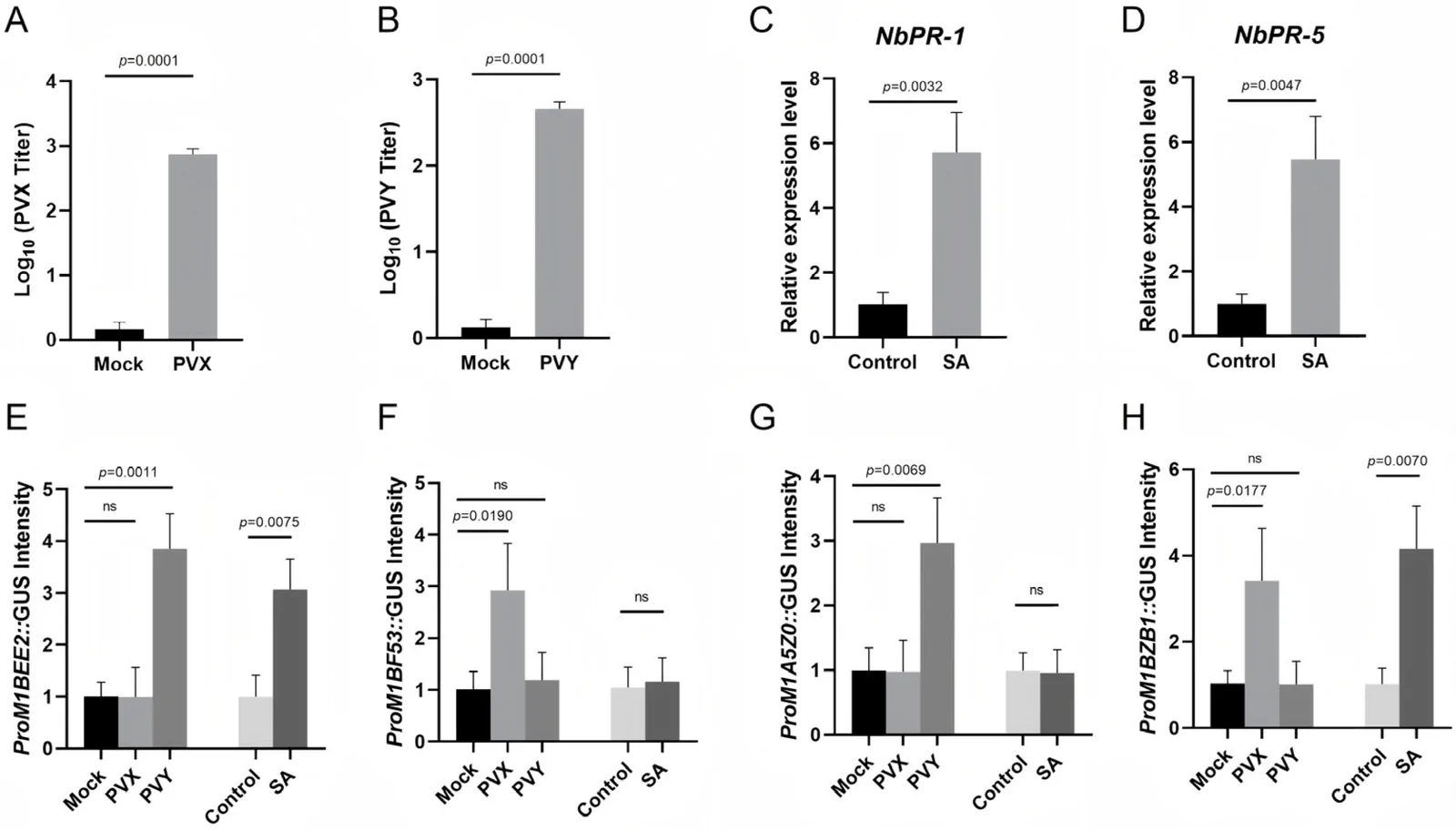
Promoter activities of potato resistance-related genes in response to PVX, PVY and SA in *Nicotiana benthamiana*. Leaf tissues of *N. benthamiana* were harvested at 7 days post-inoculation (dpi) with PVY and PVX, or subjected to SA treatment. For the analysis, 10 leaves were pooled to reduce leaf-to-leaf variation and ensure a representative sample. Total RNA was extracted from the pooled sample. Three independent biological replicates were performed. RT-qPCR was performed using gene-specific primers. The 2^−△△Ct^ method was used for calculation of target gene expression level normalized with internal control *NbActin* mRNA level. Mock: Wild-type *N. benthamiana* without virus inoculation. Control: Wild-type *N. benthamiana* without SA treatment. (**A**,**B**) PVX and PVY virus titer detection. *N. benthamiana* leaves were inoculated with PVX or PVY, and viral accumulation was detected at 7 dpi by RT-qPCR. Virus titers are presented as log10-transformed values. The *y*-axis represents the mean log10-transformed virus titer in the leaf infiltration region and the *x*-axis represents the target gene. (**C**,**D**) Induction of defense-related genes by SA in *Nicotiana benthamiana*. Relative expression levels of the pathogenesis-related marker genes *NbPR-1* and *NbPR-5* were examined by RT-qPCR at 2 dpi. (**E**–**H**) Promoter activity assays of selected potato defense-related genes in response to PVX, PVY, and SA treatment. The promoter activities of *StM1BEE2* (**E**), *StM1BF53* (**F**), *StM1A5Z0* (**G**) and *StM1BZB1* (**H**) were evaluated using GUS reporter assay in *N. benthamiana* leaves. GUS transcript levels were quantified by RT-qPCR using GUS-specific primers. Values are means ± SD from three independent biological replicates (*n* = 3), each with three technical replicates. Error bars represent the standard deviation among biological replicates. Exact *p* values are indicated in the figure. For the viral inoculation experiment (mock, PVX, and PVY), significant differences compared with mock were determined by one-way ANOVA followed by Dunnett’s post hoc test. For the SA treatment experiment (control and SA), significant differences were determined by Student’s *t*-test. *p* < 0.05 was considered statistically significant.

The promoter regions of the above four genes were cloned, and promoter–GUS assays were performed to further validate the above results. The promoter–GUS construct was transformed into *Agrobacterium GV3101* and infiltrated to 4–6-week-old *N. benthamiana*. These plants were infected by PVX and PVY and were also treated separately with SA via leaf spraying. Viral infections were confirmed by the assay on virus titers and SA treatment was confirmed by the RT-qPCR assay on *N. benthamiana* marker genes such as *PR-1* and *PR-5* (Figure 7 A–D). GUS transcript levels were quantified by RT-qPCR using GUS-specific primers, with *NbActin* as the internal reference.

As a result, *M1BEE2* expression was induced by PVY infection and SA treatment but did not respond to PVX infection (Figure 6E and Figure 7E). *M1BF53* expression was upregulated upon PVX infection but did not respond to PVY infection and SA treatment (Figure 6F and Figure 7F). *M1A5Z0* expression was induced by PVY infection but did not respond to PVX infection and SA treatment (Figure 6G and Figure 7G). *M1BZB1* expression was upregulated upon PVX infection and SA treatment but did not respond to PVY infection (Figure 6H and Figure 7H). Taken together, these four genes show different induction and expression patterns upon virus infection and SA treatment, suggesting their potential involvement in disease response and plant immunity. However, further functional validation is required to establish their exact roles.

## 3. Discussion

Plant immunity relies on PTI and ETI, with ETI mediated by NLRs. The NBS domain functions as a molecular switch through ATP/ADP binding and hydrolysis [27,28], whereas the LRR domain participates in ligand binding and autoinhibition [29,30]. The TIR domain hydrolyzes NAD^+^ to produce second messengers, activating ETI and programmed cell death [31,32], the CC domain drives oligomerization into a resistosome [33,34], and RPW8 functions as a helper NLR [35]. These canonical domains have been extensively characterized; however, the contribution of atypical NLRs with integrated domains to immune recognition remains less understood, which is the focus of this study.

The classical immunity of plants, mainly relying on the dual-layer defense of PTI and ETI, has inherent limitations, including a narrow recognition spectrum, easy evasion by pathogen effectors, and growth costs associated with immune activation. Research is therefore increasingly focused on discovering new immune elements and regulatory mechanisms. In this context, identifying new plant immune-related proteins is important because they are reshaping disease-resistance strategies for green agriculture at both molecular and applied levels. The mining of new IDs is the key to breaking through the bottleneck of plant immune recognition and achieving precise design of crop disease-resistant breeding. First, it will expand the immune recognition spectrum of R proteins [5]. Traditional research has mostly focused on HMA or WRKY domains, while the mining of new IDs (such as Exo70, BED, KIN, etc.) has revealed more host protein types that are attacked by pathogen effector proteins [36,37,38]. The newly identified integrated Exo70 domain can recognize the key effector AVR-Pii of rice blast and wheat blast pathogens, addressing the insufficient recognition of cross-species invasive pathogens in cereal crops, thereby dealing with broad-spectrum pathogen threats [38]. The host targets of many pathogen effectors were previously unknown, and the discovery of new IDs directly corresponds to these unknown targets, helping plants to perceive the virulence factors that originally escaped monitoring. Second, it can provide a foundation for structure-oriented immune engineering. The three-dimensional structural information of the new ID allows researchers to optimize its binding interface with effector factors through site-directed mutagenesis [39]. After modification of the Exo70 domain in the barley RGH2 receptor, the binding affinity is increased by 5–10 times, significantly enhancing disease resistance [38]. Based on the modular characteristics of the new ID, it can be modified into other NLRs to create artificial immune receptors that can recognize new or mutated effector factors, shortening the breeding cycle [40]. Natural IDs may have weaker binding due to evolutionary pressure. By exploring new IDs and combining them with structural engineering, this limitation can be overcome to obtain hypersensitivity or broad-spectrum resistance [39]. Third, it will deepen the understanding of plant immune evolution and regulatory networks. The distribution analysis of the new ID shows that plants integrate protein domains with different functions into NLR through gene recombination, which is an efficient and conservative evolutionary strategy that helps us to understand the adaptive evolution of the immune system [21,41]. Different IDs (such as kinases and transcription factors) may trigger differentiated downstream signals, and exploring new IDs can help clarify the specific regulatory pathways from recognition to assembly of resistosomes [42,43,44]. The new ID is often used as part of Sensor NLR, and its discovery helps to design Sensor/Helper pairing combinations more rationally, improving the stability and efficiency of immune responses [45,46]. In the present study, we performed a genome-wide screening of the potato NLRome and identified 181 atypical NLR protein entries, comprising 147 NLR-IDs and 34 truncated NLRs. This provides a preliminary catalog of atypical NLRs in potato and a basis for future functional characterization, thereby contributing to the mining of new IDs in this important crop.

The altered expression patterns across Rywal, NahG-Rywal, and shRBOHD-Rywal backgrounds revealed distinct transcriptional associations of potato atypical NLRs, which may reflect differences in SA- and ROS-related signaling. In Rywal plants, which mount a hypersensitive response that restricts virus spread, only six genes were upregulated and nineteen genes were downregulated. This pattern may indicate that most atypical NLRs are either constitutively expressed at sufficient levels in the resistant background or regulated at the post-transcriptional level. In contrast, NahG-Rywal plants exhibited a reversed transcriptional pattern (seventeen upregulated, eight downregulated), consistent with the known role of SA in *Ny-1*-mediated HR against PVY and *Nb*-mediated defense against PVX [47,48]. The shRBOHD-Rywal plants showed a comparable total number of genes with altered expression (eighteen upregulated, six downregulated), which may reflect a transcriptional response to impaired ROS signaling. Notably, the directionality of regulation differed between the two backgrounds. SA deficiency was associated with more downregulated genes, whereas ROS deficiency was associated with predominantly upregulated genes. The observed expression patterns raise the possibility that SA and ROS may contribute to the regulation of these genes in different ways. The partial overlap between genes with altered expression in NahG-Rywal and shRBOHD-Rywal backgrounds is consistent with the possibility that SA- and ROS-related pathways may converge on some common transcriptional targets.

C-JID is a recently discovered ID in R proteins. When TNL-type resistance protein RPP1 and its effector protein ATR1 were co-expressed, a highly purified active RPP1-ATR1 complex was produced and was named the RPP1 resistosome. The C-terminus of RPP1 contains a novel domain, C-JID, which also directly participates in the recognition of ATR1 [49]. The C-JID has been identified in a number of other TNL-type immune receptors across diverse plant taxa. For instance, the C-JID (or the related post-LRR region) is present in the *Arabidopsis* TNL RPS4, which forms a functional heterodimer with RRS1 for effector recognition. Mutations within the predicted RPS4 C-JID have been shown to abolish *RRS1/RPS4*-mediated immunity, underscoring its functional importance beyond RPP1 [49]. In Solanaceous crops, the C-JID has been detected in Ry_sto_-like proteins from wild potato relatives, which confer resistance to PVY [50]. Large-scale genomic surveys across angiosperms have further revealed that C-JID, together with DUF3542, is among the most prevalent integrated domains in TNL genes [4,51]. The sequence-diversified C-JID is shared by many other TNLs in diverse plant species, suggesting a broader role in the detection of various pathogen effectors. In our study, we selected four C-JID-containing genes (*M1BEE2*, *M1BF53*, *M1A5Z0*, and *M1BZB1*) for expression analysis. Together with its presence in diverse plant species, including potato, these findings suggest that this integrated domain may serve as a conserved module for effector recognition and immune activation across different NLRs.

Expression analysis has become a useful approach for discovering plant immune factors by comparing gene expression differences between pathogen infection and normal conditions. The four selected C-JID-encoding genes exhibited distinct expression patterns after PVX and PVY infection and SA treatment, and these patterns were validated by RT-qPCR and promoter–GUS assays. It should be noted that the promoter–GUS assay was performed by expressing potato promoters in the heterologous host *N. benthamiana*, which provides only indirect evidence for promoter activity and may not fully reflect the regulatory behavior of these promoters in potato. These observations are consistent with, but do not establish, a role for these genes in antiviral responses or effector recognition. Functional validation of this possibility would require loss- and gain-of-function analyses, disease phenotyping, viral accumulation assays, and direct effector interaction studies. This possibility is consistent with the established view that integrated domains in NLRs undergo rapid diversification under pathogen pressure, as documented for HMA domains in rice *Pik* alleles, where different allelic variants have independently evolved high-affinity binding to distinct AVR-Pik effectors through convergent evolutionary trajectories [52]. Whether the potato C-JID genes similarly represent an allelic series or a family of paralogs with subfunctionalized recognition specificities remains to be determined through comprehensive haplotype analysis across diverse potato germplasms.

The differential SA responsiveness among these genes adds another layer of complexity to their regulation. *M1BEE2* and *M1BZB1*, which are induced by SA, may therefore be linked to SA-responsive transcriptional programs, whereas *M1BF53* and *M1A5Z0* showed no SA response under the conditions tested. This distinction may be relevant in the context of potato antiviral immunity, where *Ry_sto_*-mediated extreme resistance to PVY operates independently of SA, while *Ny-1*-mediated hypersensitive response requires SA accumulation [47,53]. However, whether this distinction is functionally relevant to the four C-JID genes remains speculative. Recent evidence has revealed that the C-JID domain of RRS1 can be phosphorylated by the receptor-like cytoplasmic kinase BIK1 [54], raising the possibility that post-translational modification contributes an additional layer of regulation; whether this could account for the divergent SA responsiveness observed here remains to be tested. Whether the potato C-JID proteins are similarly subject to phosphorylation-dependent modulation upon viral infection warrants future investigation. Collectively, our results indicate dynamic transcriptional regulation of C-JID-encoding NLR-ID genes. The virus- and SA-associated expression patterns reported here provide a rationale for functional characterization of this gene family.

Several limitations of this study should be acknowledged. The classification of atypical NLRs relies on database annotation and domain prediction. The public transcriptome analysis used published normalized CPM values without reprocessing raw reads or formal differential expression testing; the reported expression changes are therefore descriptive and correlative rather than statistically inferred or causal. Functional validation was limited to four C-JID-encoding genes, and promoter activity was assessed in the heterologous host *N. benthamiana*. These limitations define the scope of the present findings and provide a basis for future functional studies.

## 4. Materials and Methods

### 4.1. Gene Screening, Domain Prediction, and Phylogenetic Analyses

Candidate NLR protein entries were retrieved from the NLRscape database (https://nlrscape.biochim.ro/, accessed on 12 July 2026), corresponding to the publicly available release described in Martin et al. (2023) [55]. NLRscape curates plant NLR proteins from UniProtKB based on the presence of at least one NBS-like domain and provides comprehensive domain annotations. All 642 protein entries annotated for *Solanum tuberosum* were downloaded and used as the starting dataset; these entries were considered NLR-related by NLRscape. These sequences were further verified by BLAST searches against the SpudDB potato genome database (DM v6.1). Of the 642 NLR protein entries, 44 lacked an assignable PGSC gene ID and were designated as “Unknown”; the remaining 598 entries were assigned to 427 unique PGSC gene IDs. Therefore, the 642 entries represented 427 uniquely identified gene loci, with multiple entries sometimes corresponding to the same locus. The 44 Unknown entries were retained at the protein-entry level for domain-based analyses but were excluded from unique locus counts. For the 193 selected entries used for downstream analyses, 191 mapped to 161 unique PGSC gene loci, while the remaining two entries (Q93YA7 and Q93YA6) lacked an identified PGSC gene ID. All downstream analyses remained at the protein-entry level, and the reported numbers refer to NLR protein entries unless explicitly stated as gene loci. The domain architecture data obtained from the NLRscape platform were used to classify the 181 atypical NLR protein entries (truncated or ID-containing) into four classes: T (TIR-containing), C (CC-containing), N (NBS-containing), and X (atypical), based on the N-terminal domain composition. An entry was defined as an NLR-ID if it contained a demonstrable integrated domain (ID), regardless of whether its canonical domains were truncated. An entry was defined as a truncated NLR if it lacked a detectable ID but possessed truncated canonical domains. The ID was defined as any domain other than NBS, LRR, CC, TIR, and RPW8 with a Pfam or SMART annotation e-value ≤ 0.001. Among these 181 atypical NLRs, 147 contained a demonstrable integrated domain and were defined as NLR-IDs, while the remaining 34 lacked a detectable integrated domain and were defined as truncated NLRs. In addition, 12 typical CNL/TNL protein entries were included solely as reference sequences for phylogenetic analysis and were not included in the atypical NLR classification. Sequence alignments were performed by using the software Geneious Prime 2025.2.

For phylogenetic analyses of the C-, N-, T-, and X-class atypical NLRs (Supplementary Figures S3–S6), trees were constructed based on NBS domain (or NBS-like domain) amino acid sequences using the Maximum Likelihood (ML) method with 1000 bootstrap replicates. For phylogenetic analysis of the four C-JID-containing potato proteins and their homologs (Supplementary Figure S7), the tree was constructed based on multiple sequence alignment of homologous proteins using the Neighbor-Joining (NJ) method with 1000 bootstrap replicates. NJ was used here because this analysis included a relatively large set of full-length homologs from diverse plant species, and the aim was to display the clustering of the four potato C-JID proteins with their homologs rather than to resolve deep phylogenetic relationships. The chromosomal locations of the genes encoding the candidate proteins on 12 chromosomes of potato were visualized using the MapGene2Chrom website (http://mg2c.iask.in/mg2c_v2.0/) (accessed on 12 July 2026). The reported numbers refer to NLR protein entries and thus overestimate the number of unique NLR loci, because multiple protein entries can correspond to the same gene locus.

### 4.2. Promoter Analyses and Cis-Element Identification

The promoter sequence, around 2000 bp upstream of the translation start site, was obtained from the Ensembl Plants database (https://plants.ensembl.org/index.html) (accessed on 10 August 2026) by one-by-one sequence searching. Cis-acting regulatory elements were predicted and analyzed for the presence of various regulatory motifs including hormone-responsive elements, stress-responsive elements, and light and growth elements, using the PlantCARE database (http://bioinformatics.psb.ugent.be/webtools/plantcare/html/) (accessed on 10 August 2026) with default parameters.

### 4.3. Analysis of Public Transcriptome Data

Public transcriptome data were obtained from the GEO dataset GSE142002 [56]. The normalized counts per million (CPM) values reported in the original study were used directly; raw RNA-seq reads were not reprocessed. The dataset comprised three potato genetic backgrounds, Rywal, NahG-Rywal, and shRBOHD-Rywal, sampled at 4 days after PVY^N-Wilga^ or mock inoculation, according to the GEO sample metadata.

For each genetic background, three biological replicates were available for the cell death region (Section A), the immediately surrounding tissue (Section B), and mock-inoculated tissue. For each gene, fold change (FC) was calculated as FC = (mean CPM_treatment)/(mean CPM_control). Control values were normalized to 1 and are not shown in the figures. Genes with FC ≥ 2 were considered upregulated, and genes with FC ≤ 0.5 were considered downregulated. For the supplementary expression analyses, the mean normalized CPM values were log_2_-transformed after adding a pseudocount of 1 [log_2_(mean CPM + 1)] to avoid undefined logarithms for zero values. Log_2_ FC was calculated as log_2_(mean CPM_treatment + 1) − log_2_(mean CPM_mock + 1). No formal statistical test was performed because the analysis was based on published normalized expression values and was intended as a descriptive comparison.

For Figure 2, Figure 3, Figure 4 and Figure 5, representative atypical NLRs were selected if they showed altered expression (FC ≥ 2 or ≤0.5) in at least one of the three backgrounds (Rywal, NahG-Rywal, and shRBOHD-Rywal).

### 4.4. Plant Growth and Stress Treatment Experiments

The potato (*Solanum tuberosum* L.) cultivar Atlantic was used in this study. Virus-free tissue culture seedlings of uniform growth were selected and transplanted into pots containing sterilized soil. Plants were grown in a greenhouse under controlled conditions at 25 °C with a 16 h light/8 h dark photoperiod and 60% relative humidity. *Nicotiana benthamiana* plants were grown under the same greenhouse conditions and used at 4–6 weeks of age for virus propagation.

For viral inoculation, four-week-old potato plants at the 6- to 10-leaf stage were used. PVX and PVY inocula were prepared from systemically infected *Nicotiana benthamiana* leaves harvested 7 days post-inoculation. Briefly, 4–6-week-old *N. benthamiana* plants were mechanically inoculated with PVX or PVY, and systemically infected leaves were collected at 7 dpi. Infected leaves carrying the virus strain were ground with 0.01 M PBS buffer and silica sand to prepare virus suspension, which was then applied to the leaves of potato plants by mechanical friction inoculation. After inoculation, leaves were rinsed with distilled water to remove excess inoculum and abrasive. Mock-inoculated plants were treated with buffer alone under the same conditions. Viral infection was allowed to progress for 7 days, after which leaf tissues were collected for further analysis.

For phytohormone treatments, potato plants were treated by foliar spray with 0.5 mM salicylic acid (SA) or distilled water (mock control). All treatments were applied 48 h prior to sample collection. Treated plants were maintained under the same greenhouse conditions until tissue sampling. All experiments were performed with at least three biological replicates.

### 4.5. Total RNA Extraction, cDNA Synthesis, and RT-qPCR Experiment

For each biological replicate, 10 leaves were pooled and ground to a fine powder to reduce leaf-to-leaf variation and ensure a representative sample. Total RNA was extracted from approximately 100 mg of the pooled tissue using TRIzol reagent (YESEN, cat. 10606ES60) according to the manufacturer’s instructions. Three independent biological replicates were performed. First-strand cDNA was synthesized from 1 μg of total RNA using the HiScript II 1st Strand cDNA Synthesis Kit (Vazyme, cat. R212) with oligo(dT) primers, following the manufacturer’s protocol.

Gene-specific primers were designed using Primer Premier 5.0 software (Premier Biosoft, Palo Alto, CA, USA) with the following parameters: primer length of 18–22 bp and GC content of 40–60%. All primers used in this study are listed in Supplementary Table S4.

RT-qPCR was performed on an Applied Biosystems ViiA 7 Real-Time PCR System (Thermo Fisher Scientific, Waltham, MA, USA) using PerfectStart Green qPCR SuperMix Kit (TransGen Biotech, Beijing, China, cat. TG-AQ601) following the manufacturer’s instructions. Each sample was run in three technical replicates, and three independent biological replicates were performed. Relative expression levels were calculated using the 2^−ΔΔCt^ method. All statistical analyses were performed using GraphPad Prism 8 (La Jolla, CA, USA) and Microsoft Excel 2019 (Microsoft, Redmond, WA, USA). Data are presented as mean ± SD. For the viral inoculation experiment (mock, PVX, and PVY), statistical significance was determined by one-way ANOVA followed by Dunnett’s post hoc test for comparisons with the mock control. For the SA treatment experiment (control and SA), statistical significance was determined by Student’s *t*-test. *p* < 0.05 was considered statistically significant.

### 4.6. Promoter Cloning and Vector Construction

Genomic DNA was extracted from young leaves of potato (*Solanum tuberosum* L. cv. Atlantic) using the cetyltrimethylammonium bromide (CTAB) method according to standard protocols. The promoter regions, approximately 2000 bp upstream of the translation start codon (ATG) of the candidate genes (*M1BEE2*, *M1BF53*, *M1A5Z0*, and *M1BZB1*), were PCR-amplified from genomic DNA using a high-fidelity DNA polymerase (Vazyme, cat. P525) with gene-specific primers. The forward and reverse primers carried the corresponding Nimble Cloning (NC) universal adapter sequences (forward adapter: 5′-AGTGGTCTCTGTCCAGTCCT-3′; reverse adapter: 5′-GGTCTCAGCAGACCACAAGT-3′) [57].

The purified promoter fragments were cloned into the pNC-121-Pro binary vector using the Nimble Cloning system. The resulting *Pro::*GUS constructs were transformed into *Escherichia coli DH5α* competent cells by heat-shock transformation. Positive clones were identified by colony PCR using vector-specific primers, and the inserts were confirmed by Sanger sequencing. The verified constructs were introduced into *Agrobacterium tumefaciens* strain *GV3101* by freeze–thaw transformation method. The positive clones were stored at −80 °C in 30% (*v*/*v*) glycerol for subsequent transient transformation assays.

### 4.7. Agrobacterium-Mediated Transient Expression and Promoter–GUS Assays

*Agrobacterium tumefaciens* strain *GV3101* harboring the *Pro::*GUS recombinant vectors or the empty vector control was cultured in LB medium containing appropriate antibiotics (50 μg/mL kanamycin, 25 μg/mL rifampicin, and 25 μg/mL gentamicin) at 28 °C with shaking at 200 rpm overnight. Bacterial cells were harvested by centrifugation at 6000× *g* for 10 min at room temperature, washed once with infiltration buffer (10 mM MES, pH 5.6, 10 mM MgCl_2_, and 100 μM acetosyringone), and resuspended in the same buffer to a final OD_600_ of 1.0. For transient expression assays, the bacterial suspensions were infiltrated into the abaxial surface of fully expanded leaves of 4–6-week-old *Nicotiana benthamiana* plants using a needleless syringe.

For viral inoculation and SA treatment, the infiltrated *N. benthamiana* leaves were subjected to PVX or PVY infiltration (OD_600_ = 1.0) or SA foliar spray (0.5 mM) at 24 h post-initial infiltration, respectively. Mock-treated control plants received infiltration buffer alone (for virus assays) or the equivalent solvent solution (for SA assays) under the same conditions. Leaf discs (1 cm in diameter) were excised from the infiltrated areas at 48 h post-treatment. For quantification of GUS expression, total RNA was extracted from the remaining leaf discs using TRIzol reagent, and first-strand cDNA was synthesized, and GUS transcript levels were quantified by RT-qPCR as described in Section 4.5.

## 5. Conclusions

In this study, a genome-wide screening of the potato NLRome identified 181 atypical NLR protein entries from an initial set of 642 NLR candidates. These entries were classified into T-, C-, N-, and X-classes based on their domain architectures. Promoter *cis*-element analysis showed that the majority of these atypical NLRs contain stress- and defense-related regulatory motifs. Transcriptional profiling under Rywal, NahG-Rywal, and shRBOHD-Rywal backgrounds revealed that these atypical NLRs exhibit altered expression patterns that may be associated with SA- and ROS-related signaling. Among them, four C-JID-containing genes (*M1BEE2*, *M1BF53*, *M1A5Z0*, and *M1BZB1*) displayed virus-specific and SA-responsive expression profiles, as validated by RT-qPCR and promoter–GUS assay. These results provide a preliminary catalog of atypical NLRs in potato and offer a basis for future functional characterization of this gene family in disease resistance.

## Data Availability

The data used in this research are publicly available. The potato (*Solanum tuberosum*) NLR protein sequences analyzed in this study were retrieved from the NLRscape database (https://nlrscape.biochim.ro/, accessed on 12 July 2026), a publicly available atlas of plant NLR protein. The promoter sequences and gene annotation data were obtained from the Ensembl Plants database (https://plants.ensembl.org/index.html, accessed on 10 August 2026). The gene expression data (RNA-seq) used for transcriptional profiling were obtained from the publicly accessible NCBI Gene Expression Omnibus (GEO) database (accession no. GSE142002), as reported previously [56]. All other data generated or analyzed during this study are included in this published article and its Supplementary Materials.

## Supplementary Materials

The following supporting information can be downloaded at https://www.mdpi.com/article/10.3390/plants15213325/s1: Figure S1: Chromosomal distribution of 427 mapped NLR genes corresponding to 598 mapped NLR protein entries in *Solanum tuberosum*; Figure S2: Classification and subtype composition of 193 selected potato NLR protein entries used for downstream analyses; Figure S3: Phylogenetic relationships, promoter *cis*-regulatory landscapes, and PVY^N-Wilga^-responsive expression profiles of 35 C-class NLR protein entries in potato; Figure S4: Phylogenetic relationships, promoter *cis*-regulatory landscapes, and PVY^N-Wilga^-responsive expression profiles of 38 N-class NLR protein entries in potato; Figure S5: Phylogenetic relationships, promoter *cis*-regulatory landscapes, and PVY^N-Wilga^-responsive expression profiles of 45 T-class NLR protein entries in potato; Figure S6: Phylogenetic relationships, promoter *cis*-regulatory landscapes, and PVY^N-Wilga^-responsive expression profiles of 75 X-class NLR protein entries in potato.; Figure S7: Phylogenetic analysis of four potato proteins (M1BEE2, M1BF53, M1A5Z0, and M1BZB1) and their homologs in representative plant species; Table S1: Classification, gene mapping, and protein characteristics of 642 potato NLR protein entries; Table S2: Correspondence between PGSC gene IDs and the 642 UniProt protein entries listed in Supplementary Table S1; Table S3: The 193 selected potato NLR protein entries used for phylogenetic, *cis*-element, and transcriptomic analyses; Table S4: Primers used for RT-qPCR assays.
