# Supplementary material for: Identification and Expression Analyses of Atypical NLR Genes in Potato
Source: Plants (Basel). 2026 Oct 27;15(21):3325. doi: 10.3390/plants15213325 (PMC13765792; doi:10.3390/plants15213325)
Supplement: Supplementary file 1 [file plants-15-03325-s001.zip › Supplementary Materials.pdf]

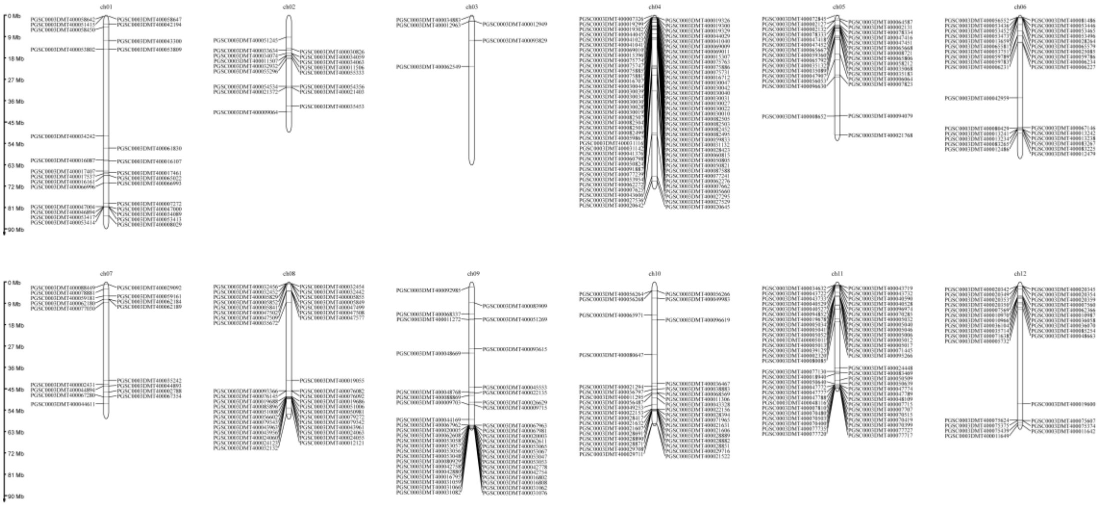

Figure S1. Chromosomal distribution of 427 mapped NLR genes corresponding to 598 mapped NLR protein entries in *Solanum tuberosum*. The cultivated potato (*Solanum tuberosum* L.) is a tetraploid (2n=4x=48) with a monoploid genome of 12 chromosomes (x=12). Of the 642 NLR protein entries retrieved from NLRscape, 44 lacked an assignable PGSC gene ID in SpudDB DM v6.1 and were designated as "Unknown"; the remaining 598 entries were assigned to 427 unique PGSC gene loci. Multiple protein entries can correspond to the same gene locus. The chromosomal positions of the 427 mapped NLR gene loci are shown across all 12 potato chromosomes; the 44 "Unknown" entries are listed in Supplementary Table S1 but are not shown on the chromosome map. The left y-axis represents chromosome length, a cylindrical shape represents a chromosome, and the short lines connected to it represent the coding position of the gene. This image was drawn by the MapGene2Chrom website (http://mg2c.iask.in/mg2c_v2.0/) (accessed on 12 July 2026).

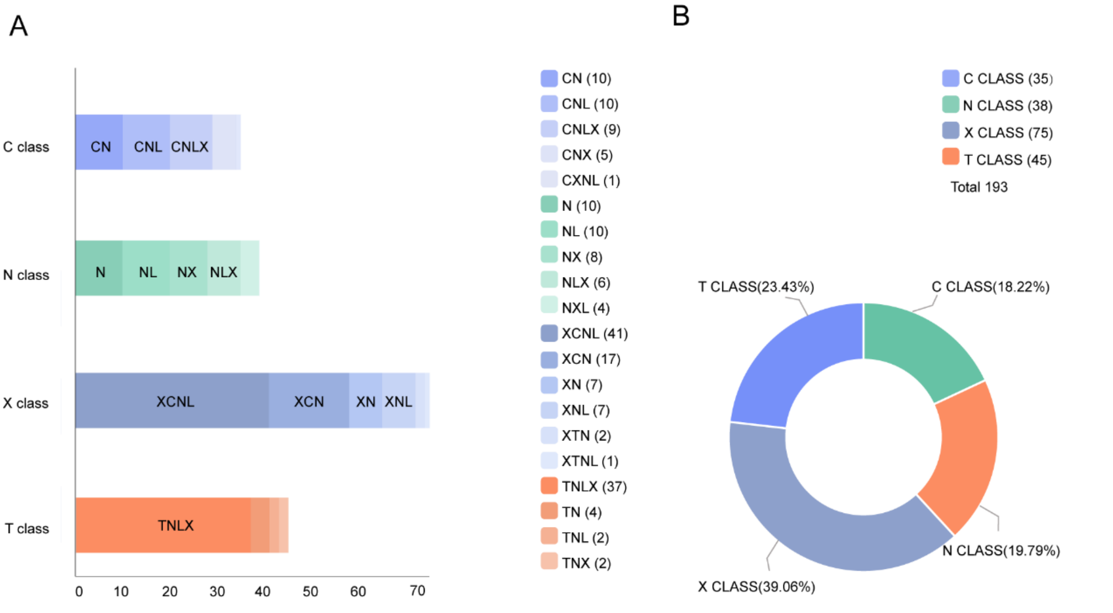

Figure S2. Classification and subtype composition of 193 selected potato NLR protein entries used for downstream analyses. **(A)** Distribution of NLR subtypes within the selected C, N, X, and T class protein sets. Horizontal stacked bars show the number and composition of individual subtypes within each class.

(B) Overall distribution of the selected NLR protein entries among the four major classes. Sector sizes indicate the relative proportion of each class, and numbers in the legend indicate the corresponding number of protein entries. These selected proteins were subsequently used for phylogenetic, *cis*-regulatory element, and transcriptomic analyses.

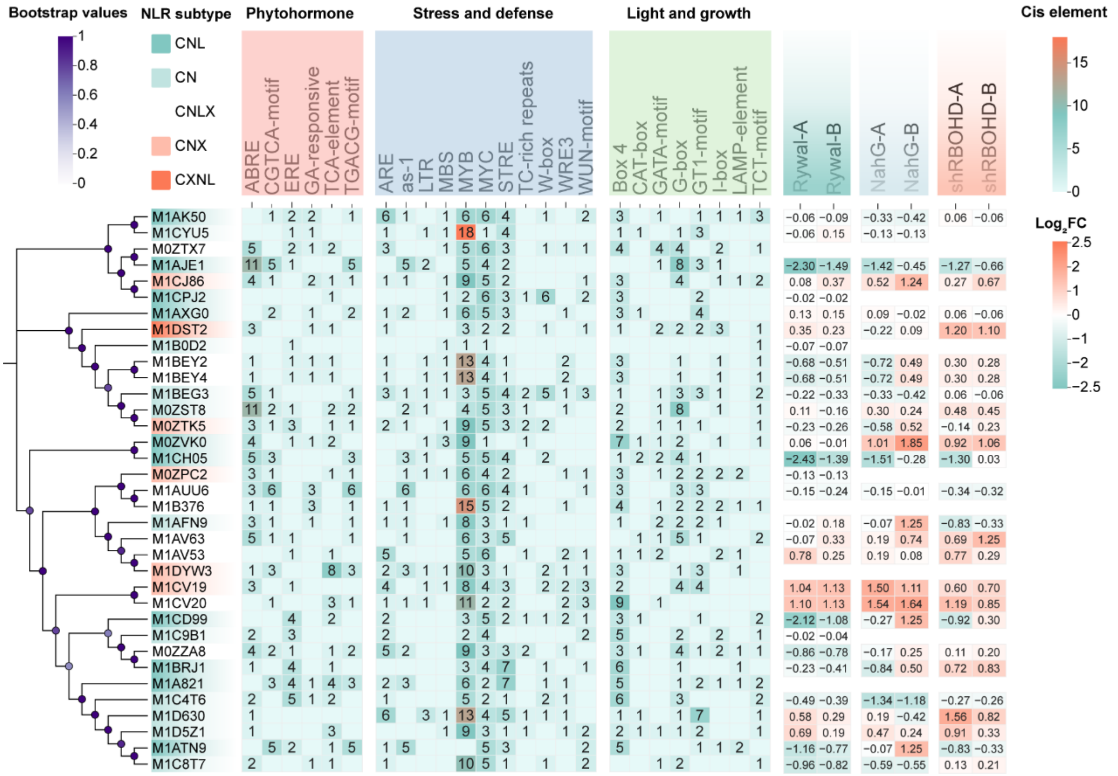

Figure S3. Phylogenetic relationships, promoter *cis*-regulatory landscapes, and PVY$^{N\text{-}Wilga}$-responsive expression profiles of 35 C-class NLR protein entries in potato. The phylogenetic tree was constructed using the NBS domain (or NBS-like domain) sequences of the 35 selected C-class NLR protein entries. Tip labels indicate UniProt accessions, and colored strips denote domain architecture-based subtypes. Node support is indicated by bootstrap values. Promoter *cis*-elements were predicted from the 2-kb upstream regions of the corresponding genes using PlantCARE and grouped into three functional categories: phytohormone, stress and defense, and light and growth. Numbers within promoter heatmap cells indicate the number of occurrences of each cis-element in the corresponding 2-kb promoter region, with color intensity proportional to cis-element abundance. Infection-responsive expression profiles were analyzed in three genetic backgrounds, Rywal, NahG-Rywal, and shRBOHD-Rywal, following PVY$^{N\text{-}Wilga}$ infection. A and B represent the cell-death region and the surrounding tissue, respectively. Expression changes are presented as $\log_2$ fold changes relative to the corresponding mock treatment, calculated as $\log_2(CPM\_{A/B} + 1) - \log_2(CPM\_{mock} + 1)$. Red and cyan indicate increased and decreased expression relative to mock, respectively, whereas values close to zero indicate little change. The same protein order was retained across the phylogenetic, promoter, and expression panels. Blank rows in the *cis*-element or expression heatmaps indicate proteins for which the corresponding promoter sequence or transcriptome data were unavailable. Within the *cis*-element heatmap, blank cells indicate that the corresponding *cis*-element was not detected in that promoter (count = 0), whereas blank cells within the expression heatmap indicate a $\log_2$ fold change of 0, corresponding to no expression change relative to the mock treatment.

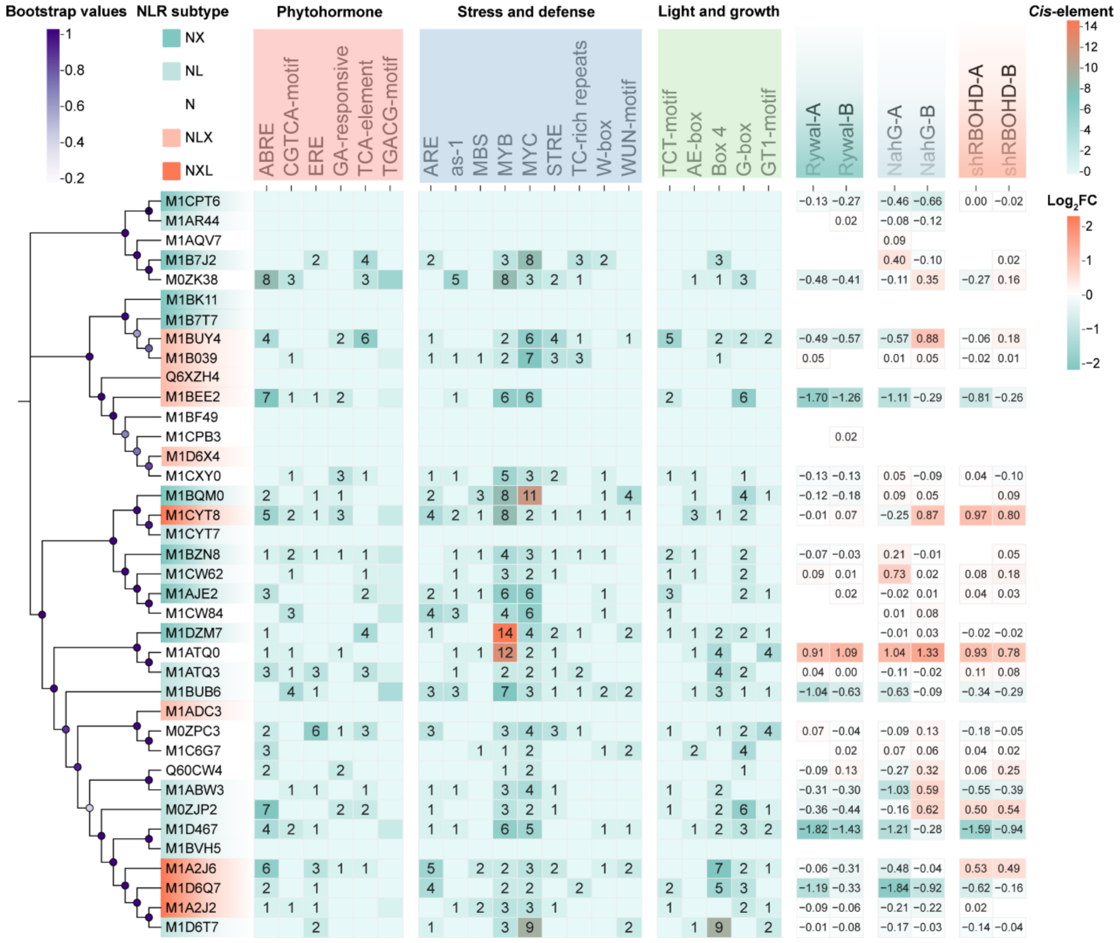

Figure S4. Phylogenetic relationships, promoter *cis*-regulatory landscapes, and PVY$^{N\text{-}Wilga}$-responsive expression profiles of 38 N-class NLR protein entries in potato. The phylogenetic tree was constructed using the NBS domain (or NBS-like domain) sequences of the 38 selected N-class NLR protein entries. Tip labels indicate UniProt accessions, and colored strips denote domain architecture-based subtypes. Node support is indicated by bootstrap values. Promoter *cis*-elements were predicted from the 2-kb upstream regions of the corresponding genes using PlantCARE and grouped into three functional categories: phytohormone, stress and defense, and light and growth. Numbers within promoter heatmap cells indicate the number of occurrences of each *cis*-element in the corresponding 2-kb promoter region, with color intensity proportional to *cis*-element abundance. Infection-responsive expression profiles were analyzed in three genetic backgrounds, Rywal, NahG-Rywal, and shRBOHD-Rywal, following PVY$^{N\text{-}Wilga}$ infection. A and B represent the cell-death region and the surrounding tissue, respectively. Expression changes are presented as $\log_2$ fold changes relative to the corresponding mock treatment, calculated as $\log_2(\text{CPM\_}_{A/B} + 1) - \log_2(\text{CPM\_}_{mock} + 1)$. Red and cyan indicate increased and decreased expression relative to mock, respectively, whereas values close to zero indicate little change. The same protein order was retained across the phylogenetic, promoter, and expression panels. Blank rows in the *cis*-element or expression heatmaps indicate proteins for which the corresponding promoter sequence or transcriptome data were unavailable. Within the *cis*-element heatmap, blank cells indicate that the corresponding *cis*-element was not detected in that promoter (count = 0), whereas blank cells within the expression heatmap indicate a $\log_2$ fold change of 0, corresponding to no expression change relative to the mock treatment.

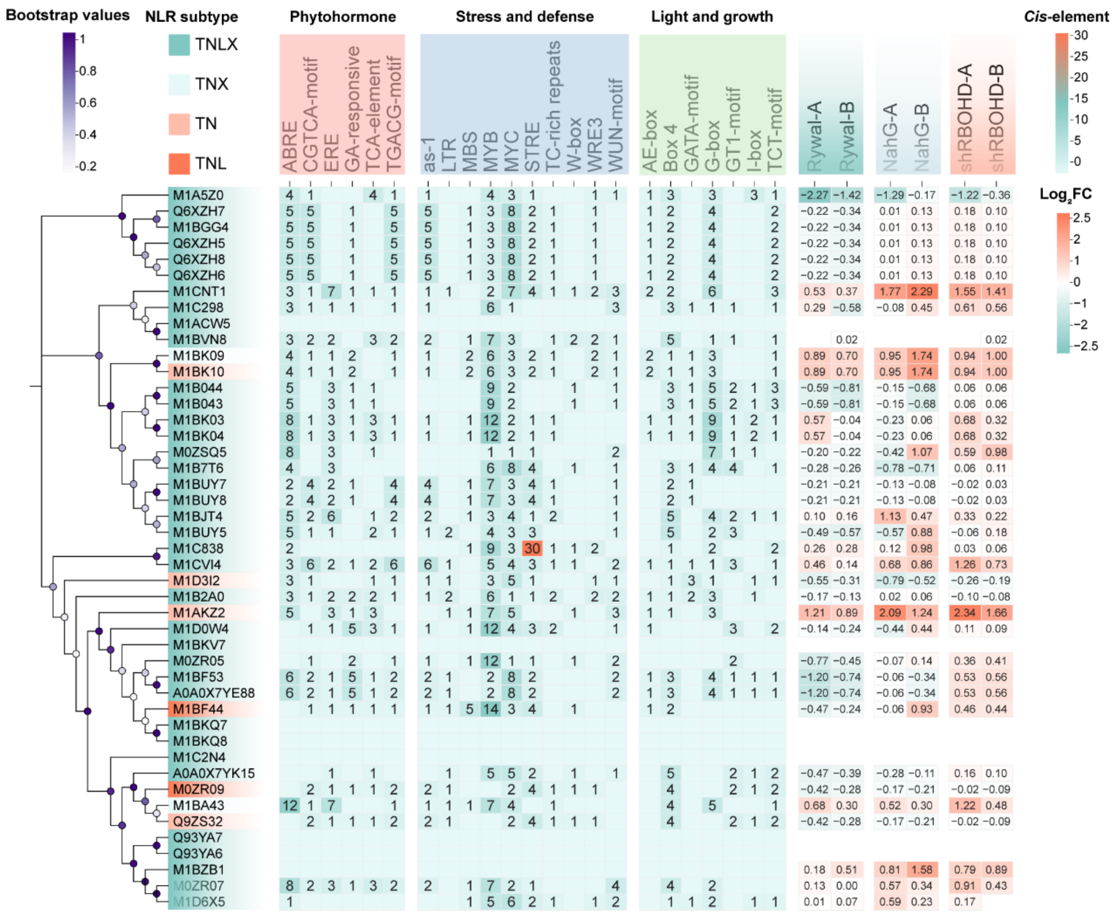

Figure S5. Phylogenetic relationships, promoter *cis*-regulatory landscapes, and PVY$^{N\text{-}Wilga}$-responsive expression profiles of 45 T-class NLR protein entries in potato. The phylogenetic tree was constructed using the NBS domain (or NBS-like domain) sequences of the 45 selected T-class NLR protein entries. Tip labels indicate UniProt accessions, and colored strips denote domain architecture-based subtypes. Node support is indicated by bootstrap values. Promoter *cis*-elements were predicted from the 2-kb upstream regions of the corresponding genes using PlantCARE and grouped into three functional categories: phytohormone, stress and defense, and light and growth. Numbers within promoter heatmap cells indicate the number of occurrences of each *cis*-element in the corresponding 2-kb promoter region, with color intensity proportional to *cis*-element abundance. Infection-responsive expression profiles were analyzed in three genetic backgrounds, Rywal, NahG-Rywal, and shRBOHD-Rywal, following PVY$^{N\text{-}Wilga}$ infection. A and B represent the cell-death region and the surrounding tissue, respectively. Expression changes are presented as $\log_2$ fold changes relative to the corresponding mock treatment, calculated as $\log_2(CPM\_{A/B} + 1) - \log_2(CPM\_{mock} + 1)$. Red and cyan indicate increased and decreased expression relative to mock, respectively, whereas values close to zero indicate little change. The same protein order was retained across the phylogenetic, promoter, and expression panels. Blank rows in the *cis*-element or expression heatmaps indicate proteins for which the corresponding promoter sequence or transcriptome data were unavailable. Within the *cis*-element heatmap, blank cells indicate that the corresponding *cis*-element was not detected in that promoter (count = 0), whereas blank cells within the expression heatmap indicate a $\log_2$ fold change of 0, corresponding to no expression change relative to the mock treatment.

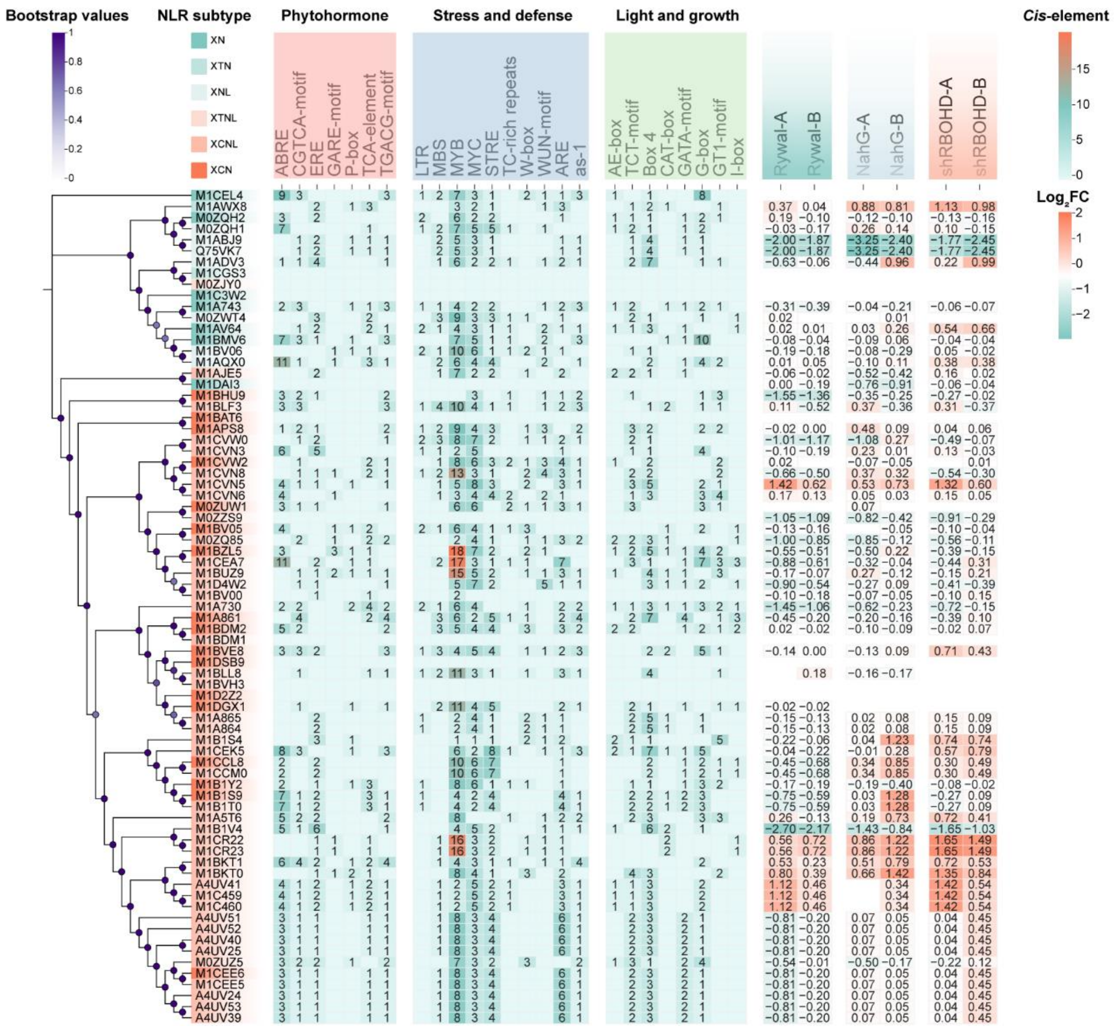

Figure S6. Phylogenetic relationships, promoter *cis*-regulatory landscapes, and PVY$^{N\text{-}Wilga}$-responsive expression profiles of 75 X-class NLR protein entries in potato. The phylogenetic tree was constructed using the NBS domain (or NBS-like domain) sequences of the 75 selected X-class NLR protein entries. Tip labels indicate UniProt accessions, and colored strips denote domain architecture-based subtypes. Node support is indicated by bootstrap values. Promoter *cis*-elements were predicted from the 2-kb upstream regions of the corresponding genes using PlantCARE and grouped into three functional categories: phytohormone, stress and defense, and light and growth. Numbers within promoter heatmap cells indicate the number of occurrences of each *cis*-element in the corresponding 2-kb promoter region, with color intensity proportional to *cis*-element abundance. Infection-responsive expression profiles were analyzed in three genetic backgrounds, Rywal, NahG-Rywal, and shRBOHD-Rywal, following PVY$^{N\text{-}Wilga}$ infection. A and B represent the cell-death region and the surrounding tissue, respectively. Expression changes are presented as $\log_2$ fold changes relative to the corresponding mock treatment, calculated as $\log_2(CPM\__{A/B} + 1) - \log_2(CPM\__{mock} + 1)$. Red and cyan indicate increased and decreased expression relative to mock, respectively, whereas values close to zero indicate little change. The same protein order was retained across the phylogenetic, promoter, and expression panels. Blank rows in the *cis*-element or expression heatmaps indicate proteins for which the corresponding promoter sequence or transcriptome data were unavailable. Within the *cis*-element heatmap, blank cells indicate that the corresponding *cis*-element was not detected in that promoter (count = 0), whereas blank cells within the expression heatmap indicate a $\log_2$ fold change of 0, corresponding to no expression change relative to the mock treatment.

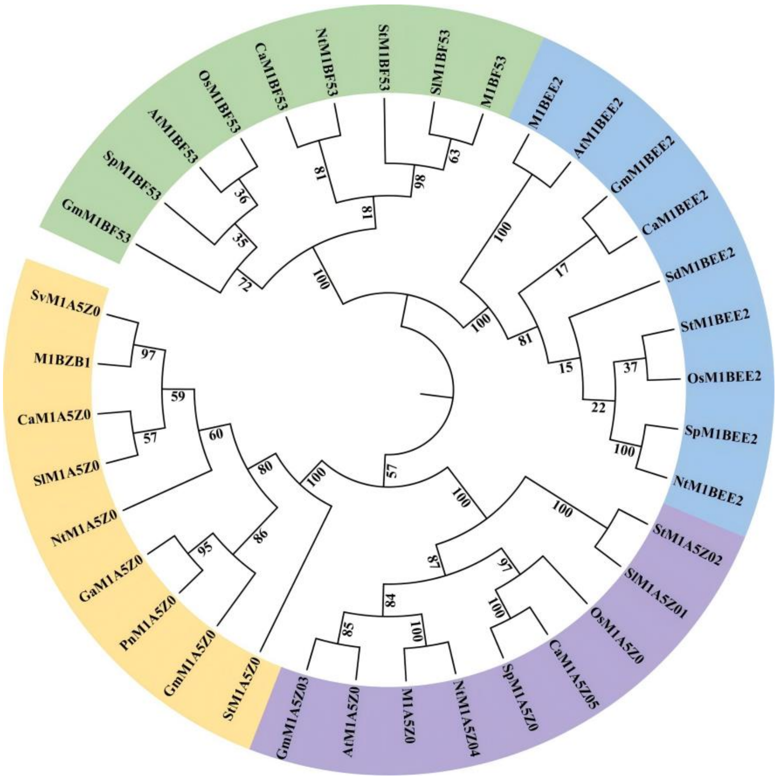

Figure S7. Phylogenetic analysis of four potato proteins (M1BEE2, M1BF53, M1A5Z0, and M1BZB1) and their homologs in representative plant species. The circular phylogenetic tree was constructed using the Neighbor-Joining (NJ) method based onmultiple sequence alignment of homologous proteins. NJ was used for this exploratory clustering analysis because the alignment contained a relatively large set of full-length homologs from diverse species. Four distinct phylogenetic clades are highlighted with background colors: blue indicates the M1BEE2 clade (potato accession *PGSC0003DMG400016808*); green indicates the M1BF53 clade (potato accession *PGSC0003DMG402016981*); purple indicates the M1A5Z0 clade (potato accession *PGSC0003DMG400006003*); and yellow indicates the M1BZB1 clade (potato accession *PGSC0003DMG400021887*). Numbers at branch nodes represent bootstrap support values from 1,000 replicates. Two-letter prefixes designate the following species: St, *Solanum tuberosum* (potato); Sl, *Solanum lycopersicum* (tomato); Sp, *Solanum pennellii*; Sd, *Solanum demissum*; Nt, *Nicotiana tabacum*; Ca, *Capsicum annuum*; At, *Arabidopsis thaliana*; Os, *Oryza sativa*; Gm, *Glycine max*; Ga, *Gossypium arboreum*; Pn, *Populus nigra*; and Sv, *Setaria viridis*.

Table S1. Classification, gene mapping, and protein characteristics of 642 potato NLR protein entries. PGSC gene IDs are indicated where an unambiguous assignment was possible. Entries annotated as "Unknown" lacked an assignable PGSC gene ID in SpudDB DM v6.1; these entries were retained at the protein-entry level for domain annotation and classification but were not counted as unique gene loci. UniProt ID, UniProt accession of each protein entry; PGSC gene ID, corresponding potato genome gene identifier; Subtype, NLR subtype assigned according to domain composition; Length, protein length in amino acids (aa); Domain organization, predicted domain architecture of the encoded protein; Description, protein name associated with the corresponding UniProt entry. Multiple UniProt protein entries may correspond to the same PGSC gene locus where alternative or redundant protein records are present.

UniProt ID	PGSC ID	Subtype	Length	Domain organization	Description
M1AR37	PGSC0003DMG400010946	CN	229 aa	CC-NBD	EDNR2GH3 protein
M0ZT66	PGSC0003DMG402002917	CN	241 aa	CC-NBD	Disease resistance protein BS2
M1BXR3	PGSC0003DMG400021477	CN	265 aa	CC-NBD	CC-NBS-LRR resistance protein
M1AFE8	PGSC0003DMG401008383	CN	289 aa	CC-NBD	Leucine-rich repeat containing protein (Nbs-LRR resistance protein)
M1ATP8	PGSC0003DMG401011522	CN	270 aa	CC-NBD	HJTR2GH1 protein
M1A868	PGSC0003DMG400006576	CN	188 aa	CC-NBD	Tospovirus resistance protein A
M1ARU9	PGSC0003DMG401011117	CN	273 aa	CC-NBD	Leucine-rich repeat containing protein
M1AV54	PGSC0003DMG400011898	CN	273 aa	CC-NBD	Tospovirus resistance protein B
M1BV14	PGSC0003DMG400020749	CN	212 aa	CC-NBD	NBS-LRR resistance protein
M1BUB7	PGSC0003DMG400020586	CN	268 aa	CC-NBD	Late blight resistance protein
M1CJT1	PGSC0003DMG401026842	CN	239 aa	CC-NBD	NRC1
M1ATQ2	PGSC0003DMG401011526	CN	207 aa	CC-NBD	SNKR2GH2 protein
M1BZ78	PGSC0003DMG400021862	CN	220 aa	CC-NBD	Disease resistance protein
M1CM40	PGSC0003DMG401027371	CN	193 aa	CC-NBD	CC-NBS-LRR protein
M1ACF6	PGSC0003DMG400007608	CN	260 aa	CC-NBD	Blight resistance protein RGA1
M1ACF4	PGSC0003DMG400007605	CN	317 aa	CC-NBD	NBS-LRR protein
M0ZQC7	PGSC0003DMG401002242	CN	279 aa	CC-NBD	Disease resistance protein BS2

M1CP25	PGSC0003DMG400027867	CN	215 aa	CC-NBD	Resistance protein PSH-RGH7
M1CJS8	PGSC0003DMG402026842	CN	290 aa	CC-NBD	NRC1
M1BDR2	PGSC0003DMG400016628	CN	127 aa	CC-NBD	Tospovirus resistance protein C
M1BAT9	PGSC0003DMG402015877	CN	212 aa	CC-NBD	NBS-coding resistance gene analog
M1DTD1	PGSC0003DMG400043650	CN	168 aa	CC-NBD	NBS-LRR resistance protein
M0ZPE2	PGSC0003DMG400001994	CN	218 aa	CC-NBD	NBS-LRR protein
M1D626	PGSC0003DMG400032576	CN	320 aa	CC-NBD	Rpi protein
M1CY33	PGSC0003DMG400030044	CN	276 aa	CC-NBD	HJTR2GH1 protein
M0ZPD9	PGSC0003DMG400001990	CN	223 aa	CC-NBD	NBS-coding resistance gene analog
M1ARU2	PGSC0003DMG400011115	CN	282 aa	CC-NBD	CC-NBS-LRR resistance protein
M1D627	PGSC0003DMG400032578	CN	179 aa	CC-NBD	HJTR2GH1 protein
A0A411D3A0	Unknown	CN	349 aa	CC-NBD	Late blight resistance protein (Fragment)
M1D1N7	PGSC0003DMG402030883	CN	269 aa	CC-NBD	Disease resistance protein RGA2
M0ZTB6	PGSC0003DMG400002947	CN	156 aa	CC-NBD	NRC1
M1CIW2	PGSC0003DMG400026573	CN	257 aa	CC-NBD	Resistance protein PSH-RGH6
M1BLE7	PGSC0003DMG400018572	CN	311 aa	CC-NBD	Disease resistance protein I2C-5
M1CJS7	PGSC0003DMG401026842	CN	305 aa	CC-NBD	NRC1
M1BR55	PGSC0003DMG400019804	CN	320 aa	CC-NBD	Disease resistance protein RGA2
M1C9I7	PGSC0003DMG400024429	CN	396 aa	CC-NBD-ARC1	NBS-coding resistance gene analog
M1C4R2	PGSC0003DMG400023250	CN	240 aa	CC-NBD-ARC1	NBS-LRR resistance protein
M1BFI8	PGSC0003DMG402017089	CN	340 aa	CC-NBD-ARC1	NBS-coding resistance gene analog
M1BRH5	PGSC0003DMG400019900	CN	372 aa	CC-NBD-ARC1	Disease resistance protein RGA4
M1ART7	PGSC0003DMG400011109	CN	413 aa	CC-NBD-ARC1	CC-NBS-LRR resistance protein
M1D623	PGSC0003DMG400032572	CN	437 aa	CC-NBD-ARC1	EDNR2GH7 protein
M1ALF3	PGSC0003DMG402009818	CN	550 aa	CC-NBD-ARC1-ARC2	RGC1
M0ZST8	PGSC0003DMG400002820	CN	532 aa	CC-NBD-ARC1-ARC2	NB-ARC domain-containing protein

M1ALF4	PGSC0003DMG402009818	CN	493 aa	CC-NBD-ARC1-ARC2	RGC1
M1BEG3	PGSC0003DMG400016823	CN	428 aa	CC-NBD-ARC1-ARC2	Nucleotide binding site-leucine rich repeat protein
M1BAS7	PGSC0003DMG400015872	CN	559 aa	CC-NBD-ARC1-ARC2	NBS-LRR type resistance protein
M1C4T6	PGSC0003DMG400023273	CN	437 aa	CC-NBD-ARC1-ARC2	HJTR2GH1 protein
M1B0D2	PGSC0003DMG400013165	CN	486 aa	CC-NBD-ARC1-ARC2	Disease resistance protein RPS2
M1AFN9	PGSC0003DMG400008449	CN	383 aa	CC-NBD-ARC1-ARC2	NBS-LRR resistance protein
M1C9B1	PGSC0003DMG400024365	CN	550 aa	CC-NBD-ARC1-ARC2	Rpi-vnt1
M1D630	PGSC0003DMG400032582	CN	441 aa	CC-NBD-ARC1-ARC2	HJTR2GH1 protein
M1C8T7	PGSC0003DMG400024231	CN	505 aa	CC-NBD-ARC1-ARC2	EDNR2GH7 protein
M1CYU5	PGSC0003DMG400030239	CN	503 aa	CC-NBD-ARC1-ARC2	CC-NBS-LRR protein
M1ALF1	PGSC0003DMG401009818	CN	452 aa	CC-NBD-ARC1-ARC2	Disease resistance protein BS2
M1D5Z1	PGSC0003DMG402032547	CN	503 aa	CC-NBD-ARC1-ARC2	HJTR2GH1 protein
M0ZPD8	PGSC0003DMG400001990	CN	408 aa	CC-NBD-ARC1-ARC2	NBS-coding resistance gene analog
M0ZTC8	PGSC0003DMG400002960	CN	427 aa	CC-NBD-ARC1-ARC2	Disease resistance protein BS2
M1B3P2	PGSC0003DMG400014047	CN	542 aa	CC-NBD-ARC1-ARC2	Disease resistance protein R3a
M1ABX5	PGSC0003DMG400007469	CN	481 aa	CC-NBD-ARC1-ARC2	NRC1
M1BR49	PGSC0003DMG400019799	CN	367 aa	CC-NBD-ARC1-ARC2	Blight resistance protein SH10
M1AQV6	PGSC0003DMG400010886	CN	528 aa	CC-NBD-ARC1-ARC2	CC-NBS-LRR resistance protein
M1AR21	PGSC0003DMG401010943	CN	448 aa	CC-NBD-ARC1-ARC2	Nbs-LRR resistance protein
M1ATP4	PGSC0003DMG400011521	CN	524 aa	CC-NBD-ARC1-ARC2-CC	EDNR2GH5 protein
M1BLZ2	PGSC0003DMG400018693	CN	466 aa	CC-NBD-CC-NBD	Disease resistance protein I2
M1C319	PGSC0003DMG400022785	CN	622 aa	CC-NBD-NBD-ARC1-ARC2	Resistance protein PSH-RGH6
M1C317	PGSC0003DMG401022784	CNL	455 aa	CC-ARC1-ARC2-LRR	Resistance protein PSH-RGH6
M1ATB1	PGSC0003DMG401011427	CNL	446 aa	CC-NBD-ARC1-ARC2	CC-NBS-LRR resistance protein
M1CPJ0	PGSC0003DMG400028001	CNL	1241 aa	CC-NBD-ARC1-ARC2-LRR	CC-NBS-LRR resistance protein
Q9LKQ0	PGSC0003DMG402007871	CNL	933 aa	CC-NBD-ARC1-ARC2-LRR	RGC1 (Fragment)

M1CGI2	PGSC0003DMG401026043	CNL	888 aa	CC-NBD-ARC1-ARC2-LRR	NRC1
Q5I6L7	PGSC0003DMG401018576	CNL	1265 aa	CC-NBD-ARC1-ARC2-LRR	Potato resistance-like protein I2GA-SH23-1
M1BFF0	PGSC0003DMG400017060	CNL	990 aa	CC-NBD-ARC1-ARC2-LRR	Disease resistance protein RGA1
M1CD99	PGSC0003DMG400025259	CNL	949 aa	CC-NBD-ARC1-ARC2-LRR	NBS-LRR type disease resistance protein
M1AK50	PGSC0003DMG400009455	CNL	1251 aa	CC-NBD-ARC1-ARC2-LRR	Disease resistance protein R3a
M1AXG0	PGSC0003DMG400012466	CNL	977 aa	CC-NBD-ARC1-ARC2-LRR	NB-ARC domain-containing protein
M1A821	PGSC0003DMG400006531	CNL	850 aa	CC-NBD-ARC1-ARC2-LRR	SNKR2GH5 protein
M1AJE1	PGSC0003DMG401009305	CNL	694 aa	CC-NBD-ARC1-ARC2-LRR	Disease resistance protein RGA3
M1BRJ1	PGSC0003DMG400019913	CNL	864 aa	CC-NBD-ARC1-ARC2-LRR	Tm-2
M1ATN9	PGSC0003DMG400011517	CNL	842 aa	CC-NBD-ARC1-ARC2-LRR	HJTR2GH1 protein
M1CPJ2	PGSC0003DMG400028001	CNL	1241 aa	CC-NBD-ARC1-ARC2-LRR	CC-NBS-LRR resistance protein
M0ZVK0	PGSC0003DMG400003527	CNL	848 aa	CC-NBD-ARC1-ARC2-LRR	Disease resistance protein RGA2
M1AZF6	PGSC0003DMG400012918	CNL	1254 aa	CC-NBD-ARC1-ARC2-LRR	CC-NBS-LRR resistance protein
M1CH05	PGSC0003DMG400026161	CNL	883 aa	CC-NBD-ARC1-ARC2-LRR	NBS-LRR type disease resistance protein
M1BQY3	PGSC0003DMG400019741	CNL	908 aa	CC-NBD-ARC1-ARC2-LRR	NRC1
A6YP83	PGSC0003DMG400018720	CNL	1329 aa	CC-NBD-ARC1-ARC2-LRR	CC-NBS-LRR protein
M1ABW0	PGSC0003DMG400007462	CNL	879 aa	CC-NBD-ARC1-ARC2-LRR	NRC1
M1A8Z3	PGSC0003DMG400006785	CNL	1162 aa	CC-NBD-ARC1-ARC2-LRR	CC-NBS-LRR resistance protein
M1CEA6	PGSC0003DMG400025511	CNL	871 aa	CC-NBD-ARC1-ARC2-LRR	Plant resistance protein
M1BQE7	PGSC0003DMG400019627	CNL	876 aa	CC-NBD-ARC1-ARC2-LRR	Disease resistance protein Gpa2
M1BLF2	PGSC0003DMG401018576	CNL	1262 aa	CC-NBD-ARC1-ARC2-LRR	Disease resistance protein I2C-5
M1BPN0	PGSC0003DMG400019418	CNL	1219 aa	CC-NBD-ARC1-ARC2-LRR	CC-NBS-LRR resistance protein
M1BAT8	PGSC0003DMG401015877	CNL	717 aa	CC-NBD-ARC1-ARC2-LRR	NBS-LRR protein
M1BZ76	PGSC0003DMG400021861	CNL	887 aa	CC-NBD-ARC1-ARC2-LRR	NRC1
M1AXG7	PGSC0003DMG400012471	CNL	987 aa	CC-NBD-ARC1-ARC2-LRR	NB-ARC domain-containing protein
M1BFQ3	PGSC0003DMG400017146	CNL	1094 aa	CC-NBD-ARC1-ARC2-LRR	NBS-LRR resistance protein RGH1

M1BMQ8	PGSC0003DMG400018903	CNL	660 aa	CC-NBD-ARC1-ARC2-LRR	CC-NBS-LRR resistance protein
M1CM95	PGSC0003DMG401027402	CNL	714 aa	CC-NBD-ARC1-ARC2-LRR	Disease resistance protein R3a
M1ACA7	PGSC0003DMG401007575	CNL	728 aa	CC-NBD-ARC1-ARC2-LRR	CC-NBS-LRR resistance protein
M1C9A9	PGSC0003DMG400024363	CNL	869 aa	CC-NBD-ARC1-ARC2-LRR	Rpi-vnt1
M1BUC0	PGSC0003DMG400020587	CNL	862 aa	CC-NBD-ARC1-ARC2-LRR	Rpi-vnt1
Q9S7Q4	PGSC0003DMG402007871	CNL	912 aa	CC-NBD-ARC1-ARC2-LRR	Disease resistance protein Gpa2 (NBS-LRR protein)
M1BNH5	PGSC0003DMG401019148	CNL	922 aa	CC-NBD-ARC1-ARC2-LRR	Leucine-rich repeat-containing protein 2, LRRc2
M1ADD0	PGSC0003DMG400007872	CNL	954 aa	CC-NBD-ARC1-ARC2-LRR	NBS-LRR protein
M1CZW8	PGSC0003DMG400030498	CNL	920 aa	CC-NBD-ARC1-ARC2-LRR	Disease resistance protein BS2
M1ADB8	PGSC0003DMG400007867	CNL	906 aa	CC-NBD-ARC1-ARC2-LRR	Disease resistance protein Gpa2
M1CVH0	PGSC0003DMG400029405	CNL	966 aa	CC-NBD-ARC1-ARC2-LRR	Disease resistance protein RPM1
M1AJH8	PGSC0003DMG400009324	CNL	988 aa	CC-NBD-ARC1-ARC2-LRR	Disease resistance protein RGA4
M1ATP3	PGSC0003DMG400011520	CNL	844 aa	CC-NBD-ARC1-ARC2-LRR	Rpi protein
M1ADC9	PGSC0003DMG400007872	CNL	897 aa	CC-NBD-ARC1-ARC2-LRR	NBS-LRR protein
M1AF10	PGSC0003DMG400008292	CNL	782 aa	CC-NBD-ARC1-ARC2-LRR	NBS-LRR resistance protein
M1A1A6	PGSC0003DMG400004874	CNL	874 aa	CC-NBD-ARC1-ARC2-LRR	Resistance protein PSH-RGH7
M1AF85	PGSC0003DMG401008349	CNL	860 aa	CC-NBD-ARC1-ARC2-LRR	CC-NBS-LRR resistance protein
M1D3S7	PGSC0003DMG400031405	CNL	903 aa	CC-NBD-ARC1-ARC2-LRR	NRC1
M1B383	PGSC0003DMG400013891	CNL	926 aa	CC-NBD-ARC1-ARC2-LRR	Resistance protein PSH-RGH6
M0ZPB9	PGSC0003DMG402001979	CNL	891 aa	CC-NBD-ARC1-ARC2-LRR	Disease resistance protein BS2
M0ZPC4	PGSC0003DMG400001983	CNL	881 aa	CC-NBD-ARC1-ARC2-LRR	Disease resistance protein BS2
M1B035	PGSC0003DMG400013091	CNL	808 aa	CC-NBD-ARC1-ARC2-LRR	Disease resistance protein Gpa2
M1AV57	PGSC0003DMG400011900	CNL	759 aa	CC-NBD-ARC1-ARC2-LRR	Tospovirus resistance protein B
M1BKW7	PGSC0003DMG400018464	CNL	870 aa	CC-NBD-ARC1-ARC2-LRR	Disease resistance protein
M1CVW1	PGSC0003DMG400029505	CNL	887 aa	CC-NBD-ARC1-ARC2-LRR	NRC1
M1C4V3	PGSC0003DMG400023288	CNL	775 aa	CC-NBD-ARC1-ARC2-LRR	Rpi protein

M1ATP9	PGSC0003DMG400011523	CNL	735 aa	CC-NBD-ARC1-ARC2-LRR	HJTR2GH1 protein
M0ZPC5	PGSC0003DMG401001984	CNL	883 aa	CC-NBD-ARC1-ARC2-LRR	Disease resistance protein BS2
M1BFF2	PGSC0003DMG400017062	CNL	977 aa	CC-NBD-ARC1-ARC2-LRR	Disease resistance protein RGA1
M1CH50	PGSC0003DMG400026187	CNL	875 aa	CC-NBD-ARC1-ARC2-LRR	Nbs-LRR resistance protein
M1C8M9	PGSC0003DMG400024203	CNL	839 aa	CC-NBD-ARC1-ARC2-LRR	Disease resistance protein BS2
M1ADC2	PGSC0003DMG400007870	CNL	950 aa	CC-NBD-ARC1-ARC2-LRR	NBS-LRR protein
M1ATQ6	PGSC0003DMG400011529	CNL	775 aa	CC-NBD-ARC1-ARC2-LRR	R2
M1DV09	PGSC0003DMG400044423	CNL	906 aa	CC-NBD-ARC1-ARC2-LRR	NBS-LRR type disease resistance protein
M1BKW5	PGSC0003DMG400018464	CNL	859 aa	CC-NBD-ARC1-ARC2-LRR	Disease resistance protein
M1A8W6	PGSC0003DMG400006769	CNL	998 aa	CC-NBD-ARC1-ARC2-LRR	Leucine-rich repeat containing protein
M1DE52	PGSC0003DMG400037159	CNL	846 aa	CC-NBD-ARC1-ARC2-LRR	Leucine-rich repeat containing protein
M1C982	PGSC0003DMG400024337	CNL	844 aa	CC-NBD-ARC1-ARC2-LRR	Hero resistance protein 2 homologue
M1BKV8	PGSC0003DMG400018462	CNL	951 aa	CC-NBD-ARC1-ARC2-LRR	Disease resistance protein
M1AF26	PGSC0003DMG400008306	CNL	950 aa	CC-NBD-ARC1-ARC2-LRR	Nbs-LRR resistance protein
Q9XGF5	PGSC0003DMG402007871	CNL	937 aa	CC-NBD-ARC1-ARC2-LRR	Rx protein
M1A823	PGSC0003DMG400006533	CNL	840 aa	CC-NBD-ARC1-ARC2-LRR	Rpi protein
M1A225	PGSC0003DMG400005046	CNL	1388 aa	CC-NBD-ARC1-ARC2-LRR	Uncharacterized protein
M1BKW6	PGSC0003DMG400018464	CNL	1016 aa	CC-NBD-ARC1-ARC2-LRR	Disease resistance protein
M1AV76	PGSC0003DMG400011920	CNL	779 aa	CC-NBD-ARC1-ARC2-LRR	SNKR2GH5 protein
A6YP88	PGSC0003DMG400027407	CNL	1318 aa	CC-NBD-ARC1-ARC2-LRR	CC-NBS-LRR protein
A6YP87	PGSC0003DMG400027407	CNL	1316 aa	CC-NBD-ARC1-ARC2-LRR	CC-NBS-LRR protein
M0ZT73	PGSC0003DMG400002920	CNL	908 aa	CC-NBD-ARC1-ARC2-LRR	RGC1
M1C8M7	PGSC0003DMG400024202	CNL	843 aa	CC-NBD-ARC1-ARC2-LRR	Disease resistance protein BS2
M1C6F8	PGSC0003DMG400023645	CNL	813 aa	CC-NBD-ARC1-ARC2-LRR	Disease resistance protein BS2
M1BLZ6	PGSC0003DMG401018696	CNL	697 aa	CC-NBD-ARC1-ARC2-LRR	Disease resistance protein R3a
M0ZKN3	PGSC0003DMG400001081	CNL	851 aa	CC-NBD-ARC1-ARC2-LRR	NBS-LRR protein

M1BZI1	PGSC0003DMG400021948	CNL	1072 aa	CC-NBD-ARC1-ARC2-LRR	CC-NBS-LRR resistance protein
M1BN91	PGSC0003DMG400019083	CNL	668 aa	CC-NBD-ARC1-ARC2-LRR	NB-ARC domain-containing protein
M1DG00	PGSC0003DMG400038020	CNL	910 aa	CC-NBD-ARC1-ARC2-LRR	Disease resistance protein BS2
M1BL12	PGSC0003DMG400018490	CNL	944 aa	CC-NBD-ARC1-ARC2-LRR	NBS-LRR type disease resistance protein
M1ADC5	PGSC0003DMG402007871	CNL	901 aa	CC-NBD-ARC1-ARC2-LRR	Disease resistance protein Gpa2
M1BKW0	PGSC0003DMG400018462	CNL	910 aa	CC-NBD-ARC1-ARC2-LRR	Disease resistance protein
M1A354	PGSC0003DMG401005337	CNL	561 aa	CC-NBD-ARC1-ARC2-LRR	Late blight resistance protein Rpi-blb2
M1ATQ1	PGSC0003DMG400011525	CNL	839 aa	CC-NBD-ARC1-ARC2-LRR	EDNR2GH4 protein
M1ARI8	PGSC0003DMG400011045	CNL	1290 aa	CC-NBD-ARC1-ARC2-LRR	CC-NBS-LRR resistance protein
M1BKV9	PGSC0003DMG400018462	CNL	925 aa	CC-NBD-ARC1-ARC2-LRR	Disease resistance protein
M1AFF9	PGSC0003DMG400008393	CNL	1018 aa	CC-NBD-ARC1-ARC2-LRR	CC-NBS-LRR resistance protein
M1BUB4	PGSC0003DMG402020585	CNL	862 aa	CC-NBD-ARC1-ARC2-LRR	Rpi-vnt1
M1BGU8	PGSC0003DMG401017416	CNL	921 aa	CC-NBD-ARC1-ARC2-LRR	NB-ARC domain-containing protein
M1D3S8	PGSC0003DMG400031405	CNL	886 aa	CC-NBD-ARC1-ARC2-LRR	NRC1
M1CZW7	PGSC0003DMG400030497	CNL	866 aa	CC-NBD-ARC1-ARC2-LRR	Resistance protein PSH-RGH6
M0ZZX0	PGSC0003DMG402004578	CNL	896 aa	CC-NBD-ARC1-ARC2-LRR	Late blight resistance protein homolog R1B-16
M1BUB2	PGSC0003DMG400020584	CNL	865 aa	CC-NBD-ARC1-ARC2-LRR	Rpi-vnt1
M1BZ80	PGSC0003DMG400021863	CNL	614 aa	CC-NBD-ARC1-ARC2-LRR	Nucleotide binding site-leucine rich repeat protein
M0ZZW9	PGSC0003DMG402004578	CNL	907 aa	CC-NBD-ARC1-ARC2-LRR	Late blight resistance protein homolog R1B-16
M1ABE4	PGSC0003DMG400007344	CNL	1270 aa	CC-NBD-ARC1-ARC2-LRR	Disease resistance protein R3a
Q60CX8	PGSC0003DMG400019732	CNL	842 aa	CC-NBD-ARC1-ARC2-LRR	Putative disease resistance protein, identical
M1D7C1	PGSC0003DMG400033651	CNL	938 aa	CC-NBD-ARC1-ARC2-LRR	Nbs-LRR resistance protein
M1AV79	PGSC0003DMG400011924	CNL	800 aa	CC-NBD-ARC1-ARC2-LRR	SNKR2GH2 protein
M1BJ65	PGSC0003DMG400018050	CNL	701 aa	CC-NBD-ARC1-ARC2-LRR	Disease resistance protein RGA1
M1AV80	PGSC0003DMG400011926	CNL	834 aa	CC-NBD-ARC1-ARC2-LRR	Rpi protein
Q6TAF9	PGSC0003DMG400017060	CNL	948 aa	CC-NBD-ARC1-ARC2-LRR	Blight resistance protein SH10 (Fragment)

Q5I6L6	PGSC0003DMG401018576	CNL	1327 aa	CC-NBD-ARC1-ARC2-LRR	Potato resistance-like protein I2GA-SH23-3
A6YP90	PGSC0003DMG400027377	CNL	1289 aa	CC-NBD-ARC1-ARC2-LRR	CC-NBS-LRR protein
M1BQM1	PGSC0003DMG400019669	CNL	1322 aa	CC-NBD-ARC1-ARC2-LRR	Potato resistance I2GA-SH23-3
M1BEY0	PGSC0003DMG401016933	CNL	905 aa	CC-NBD-ARC1-ARC2-LRR	Disease resistance protein; Calcium-binding EF-hand; AAA ATPase
M1AZF4	PGSC0003DMG400012918	CNL	1184 aa	CC-NBD-ARC1-ARC2-LRR	CC-NBS-LRR resistance protein
M1C8M8	PGSC0003DMG400024203	CNL	868 aa	CC-NBD-ARC1-ARC2-LRR	Disease resistance protein BS2
M1BKW1	PGSC0003DMG400018462	CNL	938 aa	CC-NBD-ARC1-ARC2-LRR	Disease resistance protein
M1CGH8	PGSC0003DMG401026043	CNL	912 aa	CC-NBD-ARC1-ARC2-LRR	NRC1
M1BKW3	PGSC0003DMG400018462	CNL	886 aa	CC-NBD-ARC1-ARC2-LRR	Disease resistance protein
M1ATQ4	PGSC0003DMG400011527	CNL	860 aa	CC-NBD-ARC1-ARC2-LRR	SNKR2GH5 protein
M1CGI1	PGSC0003DMG404026043	CNL	886 aa	CC-NBD-ARC1-ARC2-LRR	NRC1
M1D1N3	PGSC0003DMG401030883	CNL	723 aa	CC-NBD-ARC1-ARC2-LRR	Late blight resistance protein Rpi-pta1
M1CMA4	PGSC0003DMG400027407	CNL	1283 aa	CC-NBD-ARC1-ARC2-LRR	Plant disease resistant protein
M1BGV0	PGSC0003DMG402017416	CNL	1089 aa	CC-NBD-ARC1-ARC2-LRR	Disease resistance protein; Calcium-binding EF-hand; AAA ATPase
M1DNM0	PGSC0003DMG400041458	CNL	839 aa	CC-NBD-ARC1-ARC2-LRR	Leucine-rich repeat containing protein
A6YP89	PGSC0003DMG401027402	CNL	711 aa	CC-NBD-ARC1-ARC2-LRR	CC-NBS-LRR protein (Fragment)
M1ATQ5	PGSC0003DMG400011528	CNL	851 aa	CC-NBD-ARC1-ARC2-LRR	R2
M0ZTE2	PGSC0003DMG400002980	CNL	1247 aa	CC-NBD-ARC1-ARC2-LRR	Disease resistance protein R3a
M1AG91	PGSC0003DMG400008588	CNL	1030 aa	CC-NBD-ARC1-ARC2-LRR	Disease resistance protein RGA4
M1BW62	PGSC0003DMG400021098	CNL	913 aa	CC-NBD-ARC1-ARC2-LRR	Nbs-LRR resistance protein
M0ZT72	PGSC0003DMG400002920	CNL	860 aa	CC-NBD-ARC1-ARC2-LRR	RGC1
M1ADC4	PGSC0003DMG402007871	CNL	904 aa	CC-NBD-ARC1-ARC2-LRR	Disease resistance protein Gpa2
M1BKW8	PGSC0003DMG400018464	CNL	1005 aa	CC-NBD-ARC1-ARC2-LRR	Disease resistance protein
B0FWN8	PGSC0003DMG402007871	CNL	930 aa	CC-NBD-ARC1-ARC2-LRR	Resistance protein PSH-RGH7

A1YUW8	PGSC0003DMG400020584	CNL	848 aa	CC-NBD-ARC1-ARC2-LRR	Tm-2 ToMV resistance protein
B0FWN7	PGSC0003DMG402007871	CNL	873 aa	CC-NBD-ARC1-ARC2-LRR	Resistance protein PSH-RGH6
M1BUB3	PGSC0003DMG401020585	CNL	626 aa	CC-NBD-ARC1-ARC2-LRR	Rpi-vnt1
M1ABV9	PGSC0003DMG401007461	CNL	847 aa	CC-NBD-ARC1-ARC2-LRR	NRC1
M1BQW9	PGSC0003DMG400019732	CNL	885 aa	CC-NBD-ARC1-ARC2-LRR	Disease resistance protein
A0A6M5WSJ6	Unknown	CNL	823 aa	CC-NBD-ARC1-ARC2-LRR	Coiled-coil nucleotide-binding leucine-rich repeat protein
M1BAS6	PGSC0003DMG400015872	CNL	837 aa	CC-NBD-ARC1-ARC2-LRR	NBS-LRR type resistance protein
M1A918	PGSC0003DMG400006800	CNL	888 aa	CC-NBD-ARC1-ARC2-LRR	NBS-LRR protein
M1A8W7	PGSC0003DMG400006769	CNL	1111 aa	CC-NBD-ARC1-ARC2-LRR	Leucine-rich repeat containing protein
M1B2N3	PGSC0003DMG400013721	CNL	1348 aa	CC-NBD-ARC1-ARC2-LRR	Plant disease resistant protein
M1BKW2	PGSC0003DMG400018462	CNL	938 aa	CC-NBD-ARC1-ARC2-LRR	Disease resistance protein
M1D1J6	PGSC0003DMG400030855	CNL	995 aa	CC-NBD-ARC1-ARC2-LRR	Disease resistance protein RGA1
M1CJ87	PGSC0003DMG400026666	CNL	780 aa	CC-NBD-ARC1-ARC2-LRR	CC-NBS-LRR resistance protein
M1AXG9	PGSC0003DMG400012472	CNL	981 aa	CC-NBD-ARC1-ARC2-LRR	Disease resistance protein
M1BV01	PGSC0003DMG400020736	CNL	839 aa	CC-NBD-ARC1-ARC2-LRR	Late blight resistance protein Rpi-blb2
Q5I6L8	PGSC0003DMG401018576	CNL	1282 aa	CC-NBD-ARC1-ARC2-LRR	Potato late blight resistance protein R3a
M1BQY0	PGSC0003DMG400019740	CNL	884 aa	CC-NBD-ARC1-ARC2-LRR	Disease resistance protein
A6YP82	PGSC0003DMG400027407	CNL	1217 aa	CC-NBD-ARC1-ARC2-LRR	CC-NBS-LRR protein
M1A232	PGSC0003DMG400005052	CNL	1139 aa	CC-NBD-ARC1-ARC2-LRR	Disease resistance protein RGA3
M1BL13	PGSC0003DMG400018490	CNL	939 aa	CC-NBD-ARC1-ARC2-LRR	NBS-LRR type disease resistance protein
M1BQX0	PGSC0003DMG400019732	CNL	879 aa	CC-NBD-ARC1-ARC2-LRR	Disease resistance protein
M1BCN1	PGSC0003DMG400016372	CNL	941 aa	CC-NBD-ARC1-ARC2-LRR	Resistance protein PSH-RGH7
M1APS6	PGSC0003DMG400010612	CNL	883 aa	CC-NBD-ARC1-ARC2-LRR	HJTR2GH1 protein
Q6TAF8	PGSC0003DMG400030855	CNL	947 aa	CC-NBD-ARC1-ARC2-LRR	Blight resistance protein SH20 (Fragment)
A0A1Z2X6Y5	PGSC0003DMG400011525	CNL	839 aa	CC-NBD-ARC1-ARC2-LRR	Late blight disease resistant protein RPP13
Q5I6L5	PGSC0003DMG401018576	CNL	1286 aa	CC-NBD-ARC1-ARC2-LRR	Potato resistance-like protein I2GA-SH194-2

M1AXG5	PGSC0003DMG400012470	CNL	606 aa	CC-NBD-ARC1-ARC2-LRR	NB-ARC domain-containing protein
M1B036	PGSC0003DMG400013091	CNL	742 aa	CC-NBD-ARC1-ARC2-LRR	Disease resistance protein Gpa2
M0ZYV3	PGSC0003DMG400004296	CNL	824 aa	CC-NBD-ARC1-ARC2-LRR	Disease resistance protein Gpa2
M1AFG0	PGSC0003DMG400008394	CNL	1259 aa	CC-NBD-ARC1-ARC2-LRR-LRR	CC-NBS-LRR resistance protein
M0ZZC5	PGSC0003DMG401004423	CNL	1240 aa	CC-NBD-ARC1-ARC2-LRR-LRR	CC-NBS-LRR resistance protein
M1AFG4	PGSC0003DMG400008394	CNL	1265 aa	CC-NBD-ARC1-ARC2-LRR-LRR	CC-NBS-LRR resistance protein
M1AGA2	PGSC0003DMG400008595	CNL	1238 aa	CC-NBD-ARC1-ARC2-LRR-LRR	CC-NBS-LRR resistance protein
M1AFG2	PGSC0003DMG400008394	CNL	1273 aa	CC-NBD-ARC1-ARC2-LRR-LRR	CC-NBS-LRR resistance protein
M1AGA5	PGSC0003DMG400008596	CNL	1242 aa	CC-NBD-ARC1-ARC2-LRR-LRR	CC-NBS-LRR resistance protein
M0ZZC6	PGSC0003DMG401004423	CNL	1267 aa	CC-NBD-ARC1-ARC2-LRR-LRR	CC-NBS-LRR resistance protein
M1AGA4	PGSC0003DMG400008596	CNL	1266 aa	CC-NBD-ARC1-ARC2-LRR-LRR	CC-NBS-LRR resistance protein
M1CV69	PGSC0003DMG400029344	CNL	956 aa	CC-NBD-ARC1-ARC2-X-LRR	Disease resistance protein RPP13
M1ALF0	PGSC0003DMG401009818	CNL	682 aa	CC-NBD-ARC1-LRR	Disease resistance protein BS2
M1CW59	PGSC0003DMG400029586	CNL	666 aa	CC-NBD-ARC1-LRR	Resistance protein PSH-RGH6
M1C9I8	PGSC0003DMG400024430	CNL	538 aa	CC-NBD-ARC1-LRR	Disease resistance protein BS2
M1D1N4	PGSC0003DMG402030883	CNL	636 aa	CC-NBD-ARC1-LRR	Disease resistance protein RGA2
M0ZPE3	PGSC0003DMG400001996	CNL	741 aa	CC-NBD-ARC2-LRR	Resistance protein PSH-RGH6
M1DRT6	PGSC0003DMG400042937	CNL	724 aa	CC-NBD-ARC2-LRR	Resistance protein PSH-RGH6
M1CM52	PGSC0003DMG400027377	CNL	992 aa	CC-NBD-ARC2-LRR	Disease resistance protein R3a
M0ZTK9	PGSC0003DMG401003025	CNL	504 aa	CC-NBD-LRR	NB-ARC domain-containing protein
M1BXR2	PGSC0003DMG400021477	CNL	625 aa	CC-NBD-LRR	CC-NBS-LRR resistance protein
M1B376	PGSC0003DMG400013889	CNLX	767 aa	CC-NBD-ARC1-ARC2-LRR-X	Disease resistance protein Gpa2
M1AV53	PGSC0003DMG400011898	CNLX	912 aa	CC-NBD-ARC1-ARC2-LRR-X	Tospovirus resistance protein B
M1BEY2	PGSC0003DMG401016933	CNLX	1148 aa	CC-NBD-ARC1-ARC2-LRR-X	Disease resistance protein; Calcium-binding EF-hand; AAA ATPase

M1BEY4	PGSC0003DMG401016933	CNLX	1314 aa	CC-NBD-ARC1-ARC2-LRR-X	Disease resistance protein; Calcium-binding EF-hand; AAA ATPase
M0ZTX7	PGSC0003DMG400003104	CNLX	1178 aa	CC-NBD-ARC1-ARC2-LRR-X-LRR	Nbs-LRR resistance protein
M0ZZA8	PGSC0003DMG400004413	CNLX	770 aa	CC-NBD-ARC1-ARC2-X-LRR	Tm-2 protein
M1AV63	PGSC0003DMG400011906	CNLX	766 aa	CC-NBD-ARC1-ARC2-X-LRR	Tospovirus resistance protein D
M1AUU6	PGSC0003DMG400011809	CNLX	472 aa	CC-NBD-X-LRR	Disease resistance protein BS2
M1CV20	PGSC0003DMG400029314	CNLX	944 aa	CC-X-NBD-ARC1-ARC2-LRR	Disease resistance protein RPM1
M1CJ86	PGSC0003DMG400026666	CNX	1360 aa	CC-NBD-ARC1-ARC2-LRR-LRR-X	Fructose-bisphosphate aldolase (EC 4.1.2.13)
M0ZPC2	PGSC0003DMG400001981	CNX	592 aa	CC-NBD-ARC1-ARC2-X	NBS-LRR protein
M0ZTK5	PGSC0003DMG401003025	CNX	571 aa	CC-NBD-ARC1-ARC2-X	NB-ARC domain-containing protein
M1DYW3	PGSC0003DMG400046201	CNX	246 aa	CC-NBD-X	Tomato spotted wilt virus resistance protein
M1CV19	PGSC0003DMG400029313	CNX	503 aa	CC-X-NBD-ARC1	R2 protein
M1DST2	PGSC0003DMG400043400	CXNL	1268 aa	CC-X-NBD-ARC1-ARC2-LRR	Disease resistance protein RGA2
M1BZW6	PGSC0003DMG400022087	N	95 aa	ARC1	Nucleotide binding site-leucine rich repeat protein
M0ZZN4	PGSC0003DMG400004523	N	109 aa	ARC1	NBS-coding resistance gene analog
M1AV55	PGSC0003DMG401011899	N	90 aa	ARC1	Tospovirus resistance protein B
M1CM53	PGSC0003DMG400027377	N	77 aa	ARC1	Disease resistance protein R3a
M1BLE9	PGSC0003DMG400018573	N	180 aa	ARC1-ARC2	Disease resistance protein R3a
M1BUA9	PGSC0003DMG402020581	N	228 aa	ARC1-ARC2	NBS-coding resistance gene protein
M1CPQ2	PGSC0003DMG400028056	N	231 aa	ARC1-ARC2	Disease resistance protein RPS2
M1D0W6	PGSC0003DMG401030700	N	198 aa	ARC1-ARC2	Resistance gene
M1CM41	PGSC0003DMG402027371	N	193 aa	ARC1-ARC2	CC-NBS-LRR protein
M1CM42	PGSC0003DMG402027371	N	263 aa	ARC1-ARC2	CC-NBS-LRR protein
M1D6U1	PGSC0003DMG400033160	N	250 aa	ARC1-ARC2	TMV resistance protein N
M0ZTK3	PGSC0003DMG400003020	N	138 aa	ARC1-ARC2	Disease resistance protein R3a
M1D629	PGSC0003DMG400032581	N	158 aa	ARC1-ARC2	EDNR2GH5 protein

Q9FQ18	PGSC0003DMG400026104	N	167 aa	NBD	NBS-kinase protein Z2 (Fragment)
P93579	Unknown	N	154 aa	NBD	St332 protein (Fragment)
M0ZYW2	PGSC0003DMG400004303	N	192 aa	NBD	NBS-coding resistance gene analog
M1AS82	PGSC0003DMG400011193	N	165 aa	NBD	Tospovirus resistance protein C
M1DYV5	PGSC0003DMG400046190	N	111 aa	NBD	NB-ARC domain-containing protein
M1CYY1	PGSC0003DMG400030267	N	124 aa	NBD	Bacterial spot disease resistance protein 4
M1D1N6	PGSC0003DMG401030883	N	149 aa	NBD	Late blight resistance protein Rpi-pta1
M1D628	PGSC0003DMG400032580	N	71 aa	NBD	EDNR2GH5 protein
P93577	Unknown	N	120 aa	NBD	13.pep (Fragment)
Q38MH5	Unknown	N	166 aa	NBD	I2 (Fragment)
M1C8U1	PGSC0003DMG400024234	N	122 aa	NBD	SNKR2GH5 protein
M1B7J3	PGSC0003DMG400015031	N	126 aa	NBD	Nucleotide binding site-leucine rich repeat protein
M1D934	PGSC0003DMG400034825	N	170 aa	NBD	NBS-LRR resistance protein
A0A410HXX5	Unknown	N	107 aa	NBD	RYSC3 (Fragment)
P93578	PGSC0003DMG400026104	N	149 aa	NBD	331.pep (Fragment)
P93573	Unknown	N	152 aa	NBD	121.pep (Fragment)
P93575	PGSC0003DMG400015031	N	154 aa	NBD	St124 protein (Fragment)
P93581	Unknown	N	151 aa	NBD	333.pep (Fragment)
P93580	Unknown	N	154 aa	NBD	334.pep (Fragment)
Q38MD5	PGSC0003DMG400018572	N	178 aa	NBD	I2 (Fragment)
M1BR53	PGSC0003DMG400019803	N	125 aa	NBD	NBS-LRR resistance protein
M0ZZN5	PGSC0003DMG400004524	N	92 aa	NBD	NBS-coding resistance gene analog
P93582	Unknown	N	153 aa	NBD	3311.pep (Fragment)
P93576	PGSC0003DMG400015031	N	153 aa	NBD	125.pep (Fragment)
M1BBA6	PGSC0003DMG400016029	N	144 aa	NBD	NBS-coding resistance gene analog
M1BFI2	PGSC0003DMG400017086	N	169 aa	NBD	Disease resistance protein

M1D6T5	PGSC0003DMG400033151	N	172 aa	NBD	NBS-coding resistance gene analog
A0A451G5H9	Unknown	N	107 aa	NBD	RYSC3 (Fragment)
P93583	Unknown	N	150 aa	NBD	3313.pep (Fragment)
Q9XGF6	Unknown	N	110 aa	NBD	ADG3/2x(V-2)7a protein (Fragment)
Q38MI4	Unknown	N	239 aa	NBD-ARC1	I2 (Fragment)
M1BW09	PGSC0003DMG402021043	N	113 aa	NBD-ARC1	Disease resistance protein RGA2
Q38MC5	PGSC0003DMG400027409	N	234 aa	NBD-ARC1	I2 (Fragment)
Q38MF2	PGSC0003DMG400027409	N	234 aa	NBD-ARC1	I2 (Fragment)
Q38MC2	Unknown	N	237 aa	NBD-ARC1	I2 (Fragment)
Q38MF1	Unknown	N	239 aa	NBD-ARC1	I2 (Fragment)
M1AKU1	PGSC0003DMG400009635	N	121 aa	NBD-ARC1	NB-ARC domain-containing protein
M1CXT3	PGSC0003DMG401029961	N	201 aa	NBD-ARC1	NBS-coding resistance protein
M1BMR1	PGSC0003DMG400018907	N	211 aa	NBD-ARC1	NBS-coding resistance gene analog
Q38MH7	Unknown	N	236 aa	NBD-ARC1	I2 (Fragment)
Q38MH6	Unknown	N	237 aa	NBD-ARC1	I2 (Fragment)
Q38MC3	PGSC0003DMG401030883	N	236 aa	NBD-ARC1	I2 (Fragment)
A0A0S1LFZ7	PGSC0003DMG400007999	N	171 aa	NBD-ARC1	Putative disease resistance protein (Fragment)
Q38MF0	Unknown	N	237 aa	NBD-ARC1	I2 (Fragment)
M1C4R3	PGSC0003DMG400023253	N	105 aa	NBD-ARC1	NBS-LRR resistance protein
M1BJ60	PGSC0003DMG400018046	N	274 aa	NBD-ARC1	NBS-coding resistance gene analog
Q38MF7	PGSC0003DMG400017666	N	211 aa	NBD-ARC1	I2 (Fragment)
Q38MI8	PGSC0003DMG401030883	N	242 aa	NBD-ARC1	I2 (Fragment)
Q38MG2	Unknown	N	229 aa	NBD-ARC1	I2 (Fragment)
A0A0S1LG04	PGSC0003DMG400007999	N	172 aa	NBD-ARC1	Putative disease resistance protein (Fragment)
Q38MC4	Unknown	N	237 aa	NBD-ARC1	I2 (Fragment)
Q38MI2	PGSC0003DMG401027371	N	237 aa	NBD-ARC1	I2 (Fragment)

M0ZJP0	PGSC0003DMG400000815	N	240 aa	NBD-ARC1	NBS-coding resistance gene analog
Q38MG8	Unknown	N	239 aa	NBD-ARC1	I2 (Fragment)
Q38MI0	Unknown	N	239 aa	NBD-ARC1	I2 (Fragment)
Q38MF4	PGSC0003DMG400027377	N	233 aa	NBD-ARC1	I2 (Fragment)
M1BFF1	PGSC0003DMG400017061	N	292 aa	NBD-ARC1	Blight resistance protein SH10
Q38MD1	PGSC0003DMG401027371	N	234 aa	NBD-ARC1	I2 (Fragment)
A0A0S1LG15	PGSC0003DMG400026104	N	171 aa	NBD-ARC1	Putative disease resistance protein (Fragment)
Q38MG5	Unknown	N	239 aa	NBD-ARC1	I2 (Fragment)
Q38MJ1	Unknown	N	237 aa	NBD-ARC1	I2 (Fragment)
A0A0S1LG10	PGSC0003DMG400026187	N	177 aa	NBD-ARC1	Putative disease resistance protein (Fragment)
Q38MD2	PGSC0003DMG400018572	N	233 aa	NBD-ARC1	I2 (Fragment)
A0A0S1LG13	PGSC0003DMG400007999	N	171 aa	NBD-ARC1	Putative disease resistance protein (Fragment)
A0A0S1LG24	PGSC0003DMG400007999	N	171 aa	NBD-ARC1	Putative disease resistance protein (Fragment)
M1DR07	PGSC0003DMG400042556	N	135 aa	NBD-ARC1	NBS domain resistance protein
Q38MF3	PGSC0003DMG400017666	N	213 aa	NBD-ARC1	I2 (Fragment)
Q38MI1	Unknown	N	228 aa	NBD-ARC1	I2 (Fragment)
Q38MC7	Unknown	N	237 aa	NBD-ARC1	I2 (Fragment)
Q38ME1	PGSC0003DMG400027409	N	234 aa	NBD-ARC1	I2 (Fragment)
M1ATP5	PGSC0003DMG400011521	N	166 aa	NBD-ARC1	EDNR2GH5 protein
Q38MG9	PGSC0003DMG400027409	N	234 aa	NBD-ARC1	I2 (Fragment)
Q38MG4	PGSC0003DMG400027409	N	234 aa	NBD-ARC1	I2 (Fragment)
Q38ME5	PGSC0003DMG400018572	N	234 aa	NBD-ARC1	I2 (Fragment)
Q38MI9	Unknown	N	234 aa	NBD-ARC1	I2 (Fragment)
M0ZZX1	PGSC0003DMG400004579	N	254 aa	NBD-ARC1	NBS-coding resistance gene analog
Q38MG6	PGSC0003DMG400018572	N	231 aa	NBD-ARC1	I2 (Fragment)
Q38MD4	PGSC0003DMG400027409	N	234 aa	NBD-ARC1	I2 (Fragment)

Q38MG3	Unknown	N	239 aa	NBD-ARC1	I2 (Fragment)
Q38MF6	Unknown	N	239 aa	NBD-ARC1	I2 (Fragment)
Q38MD6	PGSC0003DMG401027371	N	239 aa	NBD-ARC1	I2 (Fragment)
A0A0S1LG02	PGSC0003DMG400007999	N	171 aa	NBD-ARC1	Putative disease resistance protein (Fragment)
Q38MH0	Unknown	N	239 aa	NBD-ARC1	I2 (Fragment)
Q5YEN6	Unknown	N	337 aa	NBD-ARC1-ARC2	R1 (Fragment)
Q60CW4	PGSC0003DMG400005577	N	306 aa	NBD-ARC1-ARC2	NB-ARC domain containing protein
Q5YEN7	Unknown	N	336 aa	NBD-ARC1-ARC2	R1 (Fragment)
M1BF49	PGSC0003DMG403016979	N	262 aa	NBD-ARC1-ARC2	NL27
Q5YEM3	Unknown	N	336 aa	NBD-ARC1-ARC2	R1 (Fragment)
Q5YEM2	PGSC0003DMG400007469	N	336 aa	NBD-ARC1-ARC2	R1 (Fragment)
Q5YEM6	Unknown	N	336 aa	NBD-ARC1-ARC2	R1 (Fragment)
M0ZK38	PGSC0003DMG402000927	N	383 aa	NBD-ARC1-ARC2	Nucleotide binding site-leucine rich repeat protein
M1AQV7	PGSC0003DMG400010886	N	368 aa	NBD-ARC1-ARC2	CC-NBS-LRR resistance protein
M0ZPC3	PGSC0003DMG400001982	N	346 aa	NBD-ARC1-ARC2	NBS-coding resistance gene analog
M1B3A5	PGSC0003DMG400013904	N	347 aa	NBD-ARC1-ARC2	Resistance protein PSH-RGH6
M1C8Z2	PGSC0003DMG400024273	N	373 aa	NBD-ARC1-ARC2	Resistance protein PSH-RGH6
Q5YEN3	PGSC0003DMG400025615	N	336 aa	NBD-ARC1-ARC2	R1 (Fragment)
H6U2T8	Unknown	N	236 aa	NBD-ARC1-ARC2	Rx-like protein (Fragment)
Q5YEM7	Unknown	N	336 aa	NBD-ARC1-ARC2	R1 (Fragment)
Q5YEQ2	PGSC0003DMG400004579	N	336 aa	NBD-ARC1-ARC2	R1 (Fragment)
M1ATQ0	PGSC0003DMG400011524	N	321 aa	NBD-ARC1-ARC2	EDNR2GH3 protein
Q5YEM0	PGSC0003DMG400025615	N	336 aa	NBD-ARC1-ARC2	R1 (Fragment)
Q5YEM1	Unknown	N	315 aa	NBD-ARC1-ARC2	R1 (Fragment)
Q5YEN4	PGSC0003DMG400021986	N	337 aa	NBD-ARC1-ARC2	R1 (Fragment)
M1CXY0	PGSC0003DMG402029995	N	254 aa	NBD-ARC1-ARC2	TMV resistance protein N

H6U2T9	PGSC0003DMG400013889	N	320 aa	NBD-ARC1-ARC2	Rx-like protein (Fragment)
M1D631	PGSC0003DMG400032584	N	363 aa	NBD-ARC1-ARC2	R2 late blight resistance protein
Q5YEK2	PGSC0003DMG400007469	N	336 aa	NBD-ARC1-ARC2	R1 (Fragment)
Q5YEQ3	PGSC0003DMG400023273	N	335 aa	NBD-ARC1-ARC2	R1 (Fragment)
Q5YEQ6	PGSC0003DMG400007469	N	336 aa	NBD-ARC1-ARC2	R1 (Fragment)
M1C6G7	PGSC0003DMG400023656	N	206 aa	NBD-ARC1-ARC2	Resistance protein PSH-RGH6
M1D6U2	PGSC0003DMG400033161	N	190 aa	NBD-ARC1-ARC2	NL25
M1AR33	PGSC0003DMG400010944	N	367 aa	NBD-ARC1-ARC2	CC-NBS-LRR resistance protein
M1D6U0	PGSC0003DMG400033159	N	184 aa	NBD-ARC1-ARC2	NL25
Q5YEK6	PGSC0003DMG400004579	N	336 aa	NBD-ARC1-ARC2	R1 (Fragment)
Q5YEN5	Unknown	N	336 aa	NBD-ARC1-ARC2	R1 (Fragment)
Q5YEK7	PGSC0003DMG400011898	N	336 aa	NBD-ARC1-ARC2	R1 (Fragment)
Q5YEL9	PGSC0003DMG400007469	N	337 aa	NBD-ARC1-ARC2	R1 (Fragment)
Q5YEM8	Unknown	N	336 aa	NBD-ARC1-ARC2	R1 (Fragment)
Q5YEK5	PGSC0003DMG400011898	N	336 aa	NBD-ARC1-ARC2	R1 (Fragment)
Q5YEK9	PGSC0003DMG400011898	N	312 aa	NBD-ARC1-ARC2	R1 (Fragment)
Q5YEK4	Unknown	N	337 aa	NBD-ARC1-ARC2	R1 (Fragment)
M1ABX4	PGSC0003DMG400007469	N	316 aa	NBD-ARC1-ARC2	NRC1
M1CW84	PGSC0003DMG400029606	N	233 aa	NBD-ARC1-ARC2	Disease resistance protein RGA2
Q5YEP9	PGSC0003DMG400004579	N	337 aa	NBD-ARC1-ARC2	R1 (Fragment)
Q5YEK8	PGSC0003DMG400013889	N	337 aa	NBD-ARC1-ARC2	R1 (Fragment)
Q5YEQ4	Unknown	N	336 aa	NBD-ARC1-ARC2	R1 (Fragment)
M1CPB3	PGSC0003DMG400027932	N	226 aa	NBD-ARC1-ARC2	NL27
P93572	PGSC0003DMG400027377	N	227 aa	NBD-ARC1-ARC2	11.pep (Fragment)
Q5YEK3	PGSC0003DMG400007469	N	338 aa	NBD-ARC1-ARC2	R1 (Fragment)
M1A2J7	PGSC0003DMG400005174	N	421 aa	NBD-ARC1-ARC2	NBS-coding resistance gene analog

M0ZSQ3	PGSC0003DMG400002799	N	311 aa	NBD-ARC1-ARC2	TMV resistance protein N
M0ZZD2	PGSC0003DMG402004425	NL	405 aa	ARC1-ARC2-LRR	CC-NBS-LRR resistance protein
M0ZTE6	PGSC0003DMG400002982	NL	949 aa	ARC1-ARC2-LRR	Potato resistance I2GA-SH23-1
M1ADC7	PGSC0003DMG401007871	NL	653 aa	ARC1-ARC2-LRR	NBS-LRR protein
M1CY34	PGSC0003DMG400030045	NL	357 aa	ARC1-ARC2-LRR	EDNR2GH5 protein
M1ARU5	PGSC0003DMG402011117	NL	881 aa	ARC1-ARC2-LRR	CC-NBS-LRR resistance protein
M1ADC0	PGSC0003DMG400007869	NL	440 aa	ARC1-ARC2-LRR	RGC1
M1BLE8	PGSC0003DMG400018572	NL	915 aa	ARC1-ARC2-LRR	Disease resistance protein I2C-5
M1BLE6	PGSC0003DMG400018570	NL	828 aa	ARC1-ARC2-LRR	Disease resistance protein R3a
M1BUA7	PGSC0003DMG402020581	NL	361 aa	ARC1-ARC2-LRR	NBS-coding resistance gene protein
M1CJT0	PGSC0003DMG403026842	NL	559 aa	ARC1-ARC2-LRR	NRC1
M1ATP7	PGSC0003DMG402011522	NL	500 aa	ARC1-ARC2-LRR	EDNR2GH4 protein
M1BR54	PGSC0003DMG400019803	NL	457 aa	ARC1-ARC2-LRR	NBS-LRR resistance protein
M1BRP9	PGSC0003DMG400019970	NL	484 aa	ARC1-ARC2-LRR	RGC1
M1CJS9	PGSC0003DMG403026842	NL	527 aa	ARC1-ARC2-LRR	NRC1
M1ADC1	PGSC0003DMG400007869	NL	441 aa	ARC1-ARC2-LRR	RGC1
M1A777	PGSC0003DMG400006316	NL	625 aa	ARC1-ARC2-LRR	NBS-LRR type disease resistance protein
M1BDV5	PGSC0003DMG400016656	NL	678 aa	ARC1-ARC2-LRR	Disease resistance protein
M1BKW4	PGSC0003DMG400018463	NL	399 aa	ARC1-ARC2-LRR	Disease resistance protein
M1AXG1	PGSC0003DMG400012466	NL	669 aa	ARC1-ARC2-LRR	NB-ARC domain-containing protein
M0ZPE0	PGSC0003DMG400001992	NL	573 aa	ARC1-ARC2-LRR	Disease resistance protein BS2
M1BL11	PGSC0003DMG400018490	NL	605 aa	ARC1-ARC2-LRR	NBS-LRR type disease resistance protein
M1ACF7	PGSC0003DMG401007609	NL	633 aa	ARC1-ARC2-LRR	NBS-LRR resistance protein
M1AFE7	PGSC0003DMG402008383	CN	474 aa	ARC1-ARC2-LRR	NBS-LRR resistance protein
Q60CX9	PGSC0003DMG400019741	NL	512 aa	NBD-ARC1-ARC2-LRR	Putative disease resistance protein, identical
M1AKT2	PGSC0003DMG400009633	NL	649 aa	NBD-ARC1-ARC2-LRR	N protein

M1D467	PGSC0003DMG400031521	NL	520 aa	NBD-ARC1-ARC2-LRR	Tospovirus resistance protein B
M0ZJN9	PGSC0003DMG400000813	NL	697 aa	NBD-ARC1-ARC2-LRR	NBS-LRR resistance protein
M1ATB4	PGSC0003DMG402011427	NL	856 aa	NBD-ARC1-ARC2-LRR	CC-NBS-LRR resistance protein
M0ZJP2	PGSC0003DMG400000816	NL	698 aa	NBD-ARC1-ARC2-LRR	Tospovirus resistance protein A
M1ANG8	PGSC0003DMG400010287	NL	618 aa	NBD-ARC1-ARC2-LRR	NB-ARC domain-containing protein
M1BZ79	PGSC0003DMG400021862	NL	456 aa	NBD-ARC1-ARC2-LRR	Disease resistance protein
M1AYC0	PGSC0003DMG400012650	NL	815 aa	NBD-ARC1-ARC2-LRR	TMV resistance protein N
M1BVH5	PGSC0003DMG402020874	NL	687 aa	NBD-ARC1-ARC2-LRR	Tospovirus resistance protein E
M1AR44	PGSC0003DMG400010953	NL	601 aa	NBD-ARC1-ARC2-LRR	Disease resistance protein RPS5
M1ALF6	PGSC0003DMG401009819	NL	467 aa	NBD-ARC1-ARC2-LRR	Resistance protein PSH-RGH6
M1A0R5	PGSC0003DMG400004756	NL	545 aa	NBD-ARC1-ARC2-LRR	Disease resistance protein
M1CEE4	PGSC0003DMG400025545	NL	729 aa	NBD-ARC1-ARC2-LRR	Late blight resistance protein homolog R1B-23
M1AEV9	PGSC0003DMG400008257	NL	603 aa	NBD-ARC1-ARC2-LRR	CC-NBS-LRR resistance protein
M1C3X4	PGSC0003DMG400022989	NL	434 aa	NBD-ARC1-ARC2-LRR	N protein
M0ZQH5	PGSC0003DMG400002278	NL	798 aa	NBD-ARC1-ARC2-LRR	NB-ARC domain-containing protein
M1A919	PGSC0003DMG400006800	NL	711 aa	NBD-ARC1-ARC2-LRR	NBS-LRR protein
M1ABW3	PGSC0003DMG400007462	NL	695 aa	NBD-ARC1-ARC2-LRR	NRC1
M0ZPE1	PGSC0003DMG400001993	NL	606 aa	NBD-ARC1-ARC2-LRR	Disease resistance protein Gpa2
M0ZYV2	PGSC0003DMG400004295	NL	827 aa	NBD-ARC1-ARC2-LRR	NBS-LRR protein
M0ZPD7	PGSC0003DMG400001989	NL	585 aa	NBD-ARC1-ARC2-LRR	PRF
M1CYT7	PGSC0003DMG402030235	NL	1056 aa	NBD-ARC1-ARC2-LRR	Disease resistance protein R3a
M1ATA8	PGSC0003DMG400011425	NL	597 aa	NBD-ARC1-ARC2-LRR	CC-NBS-LRR resistance protein
M1ART8	PGSC0003DMG400011111	NL	455 aa	NBD-ARC1-ARC2-LRR	CC-NBS-LRR resistance protein
M1ABX6	PGSC0003DMG400007471	NL	734 aa	NBD-ARC1-ARC2-LRR	NRC1
M1B115	PGSC0003DMG400013308	NL	716 aa	NBD-ARC1-ARC2-LRR	Nbs-LRR resistance protein
M1CLX6	PGSC0003DMG400027325	NL	837 aa	NBD-ARC1-ARC2-LRR	BED finger-NBS-LRR resistance protein

M1CEV6	PGSC0003DMG400025683	NL	962 aa	NBD-ARC1-ARC2-LRR	CC-NBS-LRR resistance protein
M1C4R1	PGSC0003DMG400023249	NL	712 aa	NBD-ARC1-ARC2-LRR	NBS-LRR resistance protein
M1BUB6	PGSC0003DMG403020585	NL	660 aa	NBD-ARC1-ARC2-LRR	Rpi-vnt1
M1CW62	PGSC0003DMG400029590	NL	776 aa	NBD-ARC1-ARC2-LRR	Disease resistance protein RGA3
M1CYU4	PGSC0003DMG400030238	NL	733 aa	NBD-ARC1-ARC2-LRR	CC-NBS-LRR protein
M1ATQ3	PGSC0003DMG402011526	NL	608 aa	NBD-ARC1-ARC2-LRR	EDNR2GH8 protein
M1CMA5	PGSC0003DMG400027409	NL	1042 aa	NBD-ARC1-ARC2-LRR	CC-NBS-LRR protein
M1B1G0	PGSC0003DMG400013407	NL	644 aa	NBD-ARC1-ARC2-LRR	CC-NBS-LRR class disease resistance protein
M1A5T5	PGSC0003DMG400005970	NL	725 aa	NBD-ARC1-ARC2-LRR	Late blight resistance protein
M1AXG6	PGSC0003DMG400012470	NL	614 aa	NBD-ARC1-ARC2-LRR	NB-ARC domain-containing protein
M1AKT3	PGSC0003DMG400009633	NL	650 aa	NBD-ARC1-ARC2-LRR	N protein
M1D6T7	PGSC0003DMG400033153	NL	717 aa	NBD-ARC1-ARC2-LRR	Tospovirus resistance protein C
M0ZSQ4	PGSC0003DMG400002799	NL	440 aa	NBD-ARC1-ARC2-LRR	TMV resistance protein N
M1BXQ5	PGSC0003DMG400021469	NL	855 aa	NBD-ARC1-ARC2-LRR	Nbs-LRR resistance protein
M1B2Q1	PGSC0003DMG400013736	NL	898 aa	NBD-ARC1-ARC2-LRR	BED finger-NBS-LRR resistance protein
M0ZQH4	PGSC0003DMG400002277	NL	652 aa	NBD-ARC1-ARC2-LRR	Disease resistance protein
M1ABV8	PGSC0003DMG402007461	NL	681 aa	NBD-ARC1-ARC2-LRR	NRC1
M1A1A5	PGSC0003DMG400004873	NL	658 aa	NBD-ARC1-ARC2-LRR	Resistance protein PSH-RGH6
M1BWI5	PGSC0003DMG400021169	NL	743 aa	NBD-ARC1-ARC2-LRR	Disease resistance protein
A4UV23	PGSC0003DMG400025545	NL	760 aa	NBD-ARC1-ARC2-LRR	Putative disease resistance protein
M1BYW4	PGSC0003DMG400021779	NL	1112 aa	NBD-ARC1-ARC2-LRR-LRR	CC-NBS-LRR resistance protein
M1ACW4	PGSC0003DMG400007742	NL	434 aa	NBD-ARC1-LRR	NBS-coding resistance gene protein
M0ZQG7	PGSC0003DMG400002271	NL	786 aa	NBD-ARC2-LRR	Disease resistance protein
M1BDL8	PGSC0003DMG400016600	NL	702 aa	NBD-ARC2-LRR	Tospovirus resistance protein E
M1A2J4	PGSC0003DMG400005172	NL	377 aa	NBD-LRR	NBS-coding resistance gene analog
M1ADC3	PGSC0003DMG401007871	NLX	873 aa	ARC1-ARC2-LRR-LRR-X	NBS-LRR protein

M1D0W5	PGSC0003DMG401030700	NLX	801 aa	ARC1-ARC2-LRR-X	Resistance gene
Q6XZH4	PGSC0003DMG400017317	NLX	980 aa	NBD-ARC1-ARC2-LRR-X	Nematode resistance-like protein (Fragment)
M1BEE2	PGSC0003DMG400016808	NLX	1065 aa	NBD-ARC1-ARC2-LRR-X	N protein
M1BUY4	PGSC0003DMG402020721	NLX	826 aa	NBD-ARC1-ARC2-LRR-X	Leucine-rich repeat-containing protein
M1B039	PGSC0003DMG400013094	NLX	869 aa	NBD-ARC1-ARC2-LRR-X	Leucine-rich repeat-containing protein
M1D6X4	PGSC0003DMG400033334	NLX	868 aa	NBD-ARC1-ARC2-LRR-X	Bacterial spot disease resistance protein 4
M1B7T7	PGSC0003DMG400015128	NX	288 aa	ARC1-ARC2-X	Leucine-rich repeat-containing protein
M1BQM0	PGSC0003DMG403019668	NX	386 aa	NBD-ARC1-ARC2-X	Disease resistance protein R3a
M1BK11	PGSC0003DMG402018257	NX	386 aa	NBD-ARC1-ARC2-X	Leucine-rich repeat-containing protein
M1AJE2	PGSC0003DMG402009305	NX	415 aa	NBD-ARC1-ARC2-X	Blight resistance protein T118
M1CPT6	PGSC0003DMG400028081	NX	413 aa	NBD-ARC1-ARC2-X	CC-NBS-LRR resistance protein
M1BZN8	PGSC0003DMG400022004	NX	371 aa	NBD-ARC1-ARC2-X	Disease resistance protein RGA1
M1DZM7	PGSC0003DMG400046545	NX	175 aa	NBD-X	Nbs-LRR resistance protein
M1B7J2	PGSC0003DMG400015031	NX	351 aa	NBD-X	Nucleotide binding site-leucine rich repeat protein
M1CYT8	PGSC0003DMG401030236	NXL	1118 aa	NBD-ARC1-ARC2-X-LRR	CC-NBS-LRR protein
M1D6Q7	PGSC0003DMG400033131	NXL	768 aa	NBD-ARC1-ARC2-X-LRR	Tospovirus resistance protein E
M1A2J2	PGSC0003DMG400005170	NXL	550 aa	NBD-ARC1-ARC2-X-LRR	NBS-coding resistance gene analog
M1A2J6	PGSC0003DMG400005173	NXL	768 aa	NBD-ARC1-ARC2-X-LRR	Tospovirus resistance protein A
M1BA17	PGSC0003DMG401015682	TN	261 aa	TIR-NBD	NL25
M1C8N7	PGSC0003DMG400024206	TN	423 aa	TIR-NBD-ARC1	N protein
M1CI84	PGSC0003DMG400026433	TN	445 aa	TIR-NBD-ARC1	ATP binding protein
M1BK10	PGSC0003DMG402018257	TN	542 aa	TIR-NBD-ARC1-ARC2	Leucine-rich repeat-containing protein
Q9ZS32	PGSC0003DMG402002428	TN	533 aa	TIR-NBD-ARC1-ARC2	NL25
M1D3I2	PGSC0003DMG400031318	TN	592 aa	TIR-NBD-ARC1-ARC2	TIR-NBS-LRR resistance protein
M1AKZ2	PGSC0003DMG400009686	TN	542 aa	TIR-NBD-ARC1-ARC2	Bacterial spot disease resistance protein 4
M1B208	PGSC0003DMG400013543	TNL	664 aa	TIR-NBD-ARC1-ARC2-LRR	NL27

M1BF54	PGSC0003DMG403016981	TNL	1037 aa	TIR-NBD-ARC1-ARC2-LRR	Resistance gene
M0ZQS6	PGSC0003DMG400002357	TNL	860 aa	TIR-NBD-ARC1-ARC2-LRR	Bacterial spot disease resistance protein 4
M1B034	PGSC0003DMG400013090	TNL	999 aa	TIR-NBD-ARC1-ARC2-LRR	Leucine-rich repeat-containing protein
M1C837	PGSC0003DMG400024055	TNL	1025 aa	TIR-NBD-ARC1-ARC2-LRR	Nematode resistance
M1BUY9	PGSC0003DMG400020722	TNL	581 aa	TIR-NBD-ARC1-ARC2-LRR	TMV resistance protein N
M1BA09	PGSC0003DMG400015681	TNL	974 aa	TIR-NBD-ARC1-ARC2-LRR	Resistance gene
A0A109NGX3	PGSC0003DMG400002426	TNL	953 aa	TIR-NBD-ARC1-ARC2-LRR	N-like putative resistance gene 3
M1APH0	PGSC0003DMG400010527	TNL	862 aa	TIR-NBD-ARC1-ARC2-LRR	Nematode resistance
M1D6X6	PGSC0003DMG400033334	TNL	539 aa	TIR-NBD-ARC1-ARC2-LRR	Bacterial spot disease resistance protein 4
Q9ZS31	PGSC0003DMG400021887	TNL	821 aa	TIR-NBD-ARC1-ARC2-LRR	NL27
M0ZV73	PGSC0003DMG400003445	TNL	1141 aa	TIR-NBD-ARC1-ARC2-LRR	Disease resistance protein
M1BF44	PGSC0003DMG402016979	TNL	993 aa	TIR-NBD-ARC1-ARC2-LRR	Bacterial spot disease resistance protein 4
M0ZR09	PGSC0003DMG402002428	TNL	652 aa	TIR-NBD-ARC1-ARC2-LRR	NL25
M1CI75	PGSC0003DMG401026432	TNL	582 aa	TIR-NBD-ARC1-ARC2-X	ATP binding protein
M1A5Y9	PGSC0003DMG400006003	TNL	1014 aa	TIR-NBD-ARC1-X-LRR	Nematode resistance
M1ACW5	PGSC0003DMG400007743	TNLX	1692 aa	TIR-NBD-ARC1-ARC2-LRR-NBD-ARC1-ARC2-LRR-X	TIR-NBS-LRR resistance protein
M1BUY7	PGSC0003DMG400020722	TNLX	1047 aa	TIR-NBD-ARC1-ARC2-LRR-X	TMV resistance protein N
M1BZB1	PGSC0003DMG400021887	TNLX	1127 aa	TIR-NBD-ARC1-ARC2-LRR-X	Bacterial spot disease resistance protein 4
M0ZR07	PGSC0003DMG400002427	TNLX	1197 aa	TIR-NBD-ARC1-ARC2-LRR-X	Bacterial spot disease resistance protein 4
M1B044	PGSC0003DMG400013098	TNLX	1173 aa	TIR-NBD-ARC1-ARC2-LRR-X	TMV resistance protein N
A0A0X7YK15	PGSC0003DMG401015682	TNLX	1076 aa	TIR-NBD-ARC1-ARC2-LRR-X	N-like putative resistance gene 1
M1B043	PGSC0003DMG400013098	TNLX	1196 aa	TIR-NBD-ARC1-ARC2-LRR-X	TMV resistance protein N
M1C2N4	PGSC0003DMG400022699	TNLX	1307 aa	TIR-NBD-ARC1-ARC2-LRR-X	Resistance gene
M1BUY8	PGSC0003DMG400020722	TNLX	1162 aa	TIR-NBD-ARC1-ARC2-LRR-X	TMV resistance protein N
Q6XZH7	PGSC0003DMG400017317	TNLX	1121 aa	TIR-NBD-ARC1-ARC2-LRR-X	Nematode resistance-like protein

Q6XZH5	PGSC0003DMG400017317	TNLX	1136 aa	TIR-NBD-ARC1-ARC2-LRR-X	Nematode resistance-like protein
Q6XZH8	PGSC0003DMG400017317	TNLX	1136 aa	TIR-NBD-ARC1-ARC2-LRR-X	Nematode resistance protein
M1BF53	PGSC0003DMG402016981	TNLX	1111 aa	TIR-NBD-ARC1-ARC2-LRR-X	Bacterial spot disease resistance protein 4
M1C838	PGSC0003DMG400024055	TNLX	1324 aa	TIR-NBD-ARC1-ARC2-LRR-X	Nematode resistance
A0A0X7YE88	PGSC0003DMG402016981	TNLX	1109 aa	TIR-NBD-ARC1-ARC2-LRR-X	N-like putative resistance gene 2
M1CNT1	PGSC0003DMG400027797	TNLX	1064 aa	TIR-NBD-ARC1-ARC2-LRR-X	TIR-NBS-LRR disease resistance
M1BVN8	PGSC0003DMG400020935	TNLX	1112 aa	TIR-NBD-ARC1-ARC2-LRR-X	TIR-NBS-LRR resistance protein
M1BGG4	PGSC0003DMG400017317	TNLX	1146 aa	TIR-NBD-ARC1-ARC2-LRR-X	Nematode resistance
M1BUY5	PGSC0003DMG401020721	TNLX	1105 aa	TIR-NBD-ARC1-ARC2-LRR-X	Leucine-rich repeat-containing protein
M1BKQ7	PGSC0003DMG400018428	TNLX	985 aa	TIR-NBD-ARC1-ARC2-LRR-X	Bacterial spot disease resistance protein 4
M1D6X5	PGSC0003DMG400033334	TNLX	968 aa	TIR-NBD-ARC1-ARC2-LRR-X	Bacterial spot disease resistance protein 4
M1BKV7	PGSC0003DMG400018461	TNLX	1169 aa	TIR-NBD-ARC1-ARC2-LRR-X	Resistance gene
M1BKQ8	PGSC0003DMG400018429	TNLX	1070 aa	TIR-NBD-ARC1-ARC2-LRR-X	Bacterial spot disease resistance protein 4
Q6XZH6	PGSC0003DMG400017317	TNLX	1136 aa	TIR-NBD-ARC1-ARC2-LRR-X	Nematode resistance-like protein
M1B2A0	PGSC0003DMG400013627	TNLX	1230 aa	TIR-NBD-ARC1-ARC2-LRR-X	TIR-NBS-LRR resistance protein
Q93YA7	Unknown	TNLX	1126 aa	TIR-NBD-ARC1-ARC2-LRR-X	Resistance gene-like
Q93YA6	Unknown	TNLX	1101 aa	TIR-NBD-ARC1-ARC2-LRR-X	Resistance gene-like
M1C298	PGSC0003DMG401022603	TNLX	1225 aa	TIR-NBD-ARC1-ARC2-X-LRR	ATP binding / protein binding / transmembrane receptor
M0ZR05	PGSC0003DMG400002426	TNLX	1159 aa	TIR-NBD-ARC1-ARC2-X-LRR	Resistance gene
M0ZSQ5	PGSC0003DMG400002799	TNLX	1205 aa	TIR-NBD-ARC1-ARC2-X-LRR-X	TMV resistance protein N
M1BK03	PGSC0003DMG400018256	TNLX	1227 aa	TIR-NBD-ARC1-ARC2-X-LRR-X	TMV resistance protein N
M1BJT4	PGSC0003DMG400018216	TNLX	1217 aa	TIR-NBD-ARC1-ARC2-X-LRR-X	TMV resistance protein N
M1B7T6	PGSC0003DMG400015128	TNLX	926 aa	TIR-NBD-ARC1-ARC2-X-LRR-X	Leucine-rich repeat-containing protein
M1D0W4	PGSC0003DMG401030700	TNLX	1093 aa	TIR-NBD-ARC1-ARC2-X-LRR-X	Resistance gene
M1BK04	PGSC0003DMG400018256	TNLX	1238 aa	TIR-NBD-ARC1-ARC2-X-LRR-X	TMV resistance protein N
M1CVI4	PGSC0003DMG400029415	TNLX	920 aa	TIR-NBD-ARC1-X-LRR	Nematode resistance protein

M1A5Z0	PGSC0003DMG400006003	TNLX	1239 aa	TIR-NBD-ARC1-X-LRR-X	Nematode resistance
M1BK09	PGSC0003DMG402018257	TNX	607 aa	TIR-NBD-ARC1-ARC2-X	Leucine-rich repeat-containing protein
M1BA43	PGSC0003DMG400015693	TNX	733 aa	TIR-NBD-ARC1-ARC2-X	Resistance gene
M1BUA6	PGSC0003DMG401020581	TTN	270 aa	TIR-TIR-NBD	TIR-NBS-LRR resistance protein
M1DVX2	PGSC0003DMG400044837	TTN	214 aa	TIR-TIR-NBD	Nematode resistance
M1CEE6	PGSC0003DMG400025545	XCN	650 aa	LateB-CC-NBD-ARC1-ARC2	Late blight resistance protein homolog R1B-23
M1A861	PGSC0003DMG400006570	XCN	729 aa	X-CC-NBD	Tospovirus resistance protein C
M1BVE8	PGSC0003DMG400020851	XCN	572 aa	X-CC-NBD	Tospovirus resistance protein D
M1CVW2	PGSC0003DMG400029506	XCN	624 aa	X-CC-NBD	Hero resistance protein
M0ZUW1	PGSC0003DMG400003353	XCN	365 aa	X-CC-NBD	NBS-LRR resistance protein
M1DGX1	PGSC0003DMG400038440	XCN	501 aa	X-CC-NBD	Tospovirus resistance protein D
M1BHU9	PGSC0003DMG400017666	XCN	341 aa	X-CC-NBD	Disease resistance protein I2C-5
M1BAT6	PGSC0003DMG402015877	XCN	427 aa	X-CC-NBD	NBS-coding resistance gene analog
M1DSB9	PGSC0003DMG400043186	XCN	430 aa	X-CC-NBD	Tospovirus resistance protein A
M1BDM2	PGSC0003DMG401016602	XCN	758 aa	X-CC-NBD-ARC1	Tospovirus resistance protein C
M1BV05	PGSC0003DMG400020740	XCN	712 aa	X-CC-NBD-ARC1	NBS-LRR resistance protein
M1BZL5	PGSC0003DMG400021986	XCN	813 aa	X-CC-NBD-ARC1-ARC2	Late blight resistance protein Rpi-blb2
M1B1S9	PGSC0003DMG400013490	XCN	1263 aa	X-CC-NBD-ARC1-ARC2	Prf
M1APS8	PGSC0003DMG401010614	XCN	624 aa	X-CC-NBD-ARC1-ARC2	R2
M1D2Z2	PGSC0003DMG400031185	XCN	687 aa	X-CC-NBD-X	Tospovirus resistance protein C
M1B1Y2	PGSC0003DMG401013522	XCN	1240 aa	X-LateB-X-CC-CC-NBD-ARC1-ARC2	Prf
M1CCL8	PGSC0003DMG400025099	XCN	1353 aa	X-LateB-X-CC-NBD-ARC1-ARC2	PRF
M0ZUZ5	PGSC0003DMG400003380	XCNL	898 aa	LateB-CC-NBD-ARC1-ARC2-LRR	Late blight resistance protein homolog R1A-4
M1BKT0	PGSC0003DMG400018441	XCNL	1138 aa	LateB-CC-NBD-ARC1-ARC2-LRR	Late blight resistance protein
M1C460	PGSC0003DMG400023062	XCNL	1019 aa	LateB-CC-NBD-ARC1-ARC2-LRR	Disease resistance protein
M1CEE5	PGSC0003DMG400025545	XCNL	1031 aa	LateB-CC-NBD-ARC1-ARC2-X-LRR	Late blight resistance protein homolog R1B-23

M1AJE5	PGSC0003DMG400009307	XCNL	535 aa	X-CC-ARC1-ARC2-LRR	Blight resistance protein T118
M1CVN6	PGSC0003DMG400029460	XCNL	956 aa	X-CC-ARC1-ARC2-LRR	Hero resistance protein
M1B1S4	PGSC0003DMG400013486	XCNL	2060 aa	X-CC-CC-NBD-ARC1-ARC2-LRR	Disease resistance protein
M1CVN5	PGSC0003DMG400029457	XCNL	1257 aa	X-CC-NBD-ARC1-ARC2-LRR	Hero resistance protein
M1D4W2	PGSC0003DMG400031878	XCNL	1110 aa	X-CC-NBD-ARC1-ARC2-LRR	NBS-LRR resistance protein
M0ZZS9	PGSC0003DMG400004561	XCNL	1031 aa	X-CC-NBD-ARC1-ARC2-LRR	Late blight resistance protein Rpi-blb2
M0ZQ85	PGSC0003DMG400002217	XCNL	1251 aa	X-CC-NBD-ARC1-ARC2-LRR	NBS-LRR resistance protein
M1B1T0	PGSC0003DMG400013490	XCNL	1645 aa	X-CC-NBD-ARC1-ARC2-LRR	Prf
M1A865	PGSC0003DMG400006573	XCNL	1261 aa	X-CC-NBD-ARC1-ARC2-LRR	Tospovirus resistance protein A
M1CVW0	PGSC0003DMG400029504	XCNL	1261 aa	X-CC-NBD-ARC1-ARC2-LRR	Hero resistance protein
M1CEA7	PGSC0003DMG400025512	XCNL	1265 aa	X-CC-NBD-ARC1-ARC2-LRR	Late blight resistance protein Rpi-blb2
M1BV00	PGSC0003DMG400020735	XCNL	776 aa	X-CC-NBD-ARC1-ARC2-LRR	Root-knot nematode resistance protein
M1CVN3	PGSC0003DMG400029453	XCNL	1344 aa	X-CC-NBD-ARC1-ARC2-LRR	Hero resistance protein 1 homologue
M1CVN8	PGSC0003DMG400029462	XCNL	1246 aa	X-CC-NBD-ARC1-ARC2-LRR	Hero resistance protein 1 homologue
M1BLL8	PGSC0003DMG400018619	XCNL	1324 aa	X-CC-NBD-ARC1-ARC2-LRR	Tospovirus resistance protein B
M1BUZ9	PGSC0003DMG400020732	XCNL	1301 aa	X-CC-NBD-ARC1-ARC2-LRR	NBS-LRR resistance protein
M1BVH3	PGSC0003DMG400020872	XCNL	734 aa	X-CC-NBD-ARC1-ARC2-LRR	Tospovirus resistance protein C
M1A730	PGSC0003DMG402006288	XCNL	1297 aa	X-CC-NBD-ARC1-ARC2-LRR	Tospovirus resistance protein B
M1A864	PGSC0003DMG400006573	XCNL	1282 aa	X-CC-NBD-ARC1-ARC2-LRR	Tospovirus resistance protein A
M1BDM1	PGSC0003DMG400016601	XCNL	2477 aa	X-CC-NBD-ARC1-ARC2-LRR-X-CC-NBD-ARC2-LRR	Tospovirus resistance protein A
M1BLF3	PGSC0003DMG402018576	XCNL	681 aa	X-CC-NBD-ARC2-LRR	Potato late blight resistance protein R3a
M1CR23	PGSC0003DMG400028339	XCNL	1058 aa	X-LateB-CC-NBD-ARC1-ARC2	Late blight resistance protein
M1B1V4	PGSC0003DMG400013506	XCNL	1215 aa	X-LateB-CC-NBD-ARC1-ARC2-LRR	Late blight resistance protein homolog R1B-16
M1BKT1	PGSC0003DMG400018442	XCNL	1221 aa	X-LateB-CC-NBD-ARC1-ARC2-LRR	Late blight resistance protein
M1C459	PGSC0003DMG400023062	XCNL	1243 aa	X-LateB-CC-NBD-ARC1-ARC2-LRR	Disease resistance protein

A4UV41	PGSC0003DMG400023062	XCNL	1208 aa	X-LateB-CC-NBD-ARC1-ARC2-LRR	Putative disease resistance protein
M1CR22	PGSC0003DMG400028339	XCNL	1437 aa	X-LateB-CC-NBD-ARC1-ARC2-LRR	Late blight resistance protein
M1A5T6	PGSC0003DMG400005970	XCNL	1196 aa	X-LateB-CC-NBD-ARC1-ARC2-LRR	Late blight resistance protein
A4UV52	PGSC0003DMG400025545	XCNL	1282 aa	X-LateB-CC-NBD-ARC1-ARC2-LRR	Putative disease resistance protein
A4UV24	PGSC0003DMG400025545	XCNL	1244 aa	X-LateB-CC-NBD-ARC1-ARC2-LRR	Putative disease resistance protein
A4UV39	PGSC0003DMG400025545	XCNL	1313 aa	X-LateB-CC-NBD-ARC1-ARC2-LRR	Late blight resistance protein R1
A4UV40	PGSC0003DMG400025545	XCNL	1305 aa	X-LateB-LateB-CC-NBD-ARC1-ARC2-LRR	Putative disease resistance protein
A4UV25	PGSC0003DMG400025545	XCNL	1264 aa	X-LateB-LateB-CC-NBD-ARC1-ARC2-LRR	Putative disease resistance protein
A4UV51	PGSC0003DMG400025545	XCNL	1294 aa	X-LateB-LateB-CC-NBD-ARC1-ARC2-LRR	Putative disease resistance protein
A4UV53	PGSC0003DMG400025545	XCNL	1320 aa	X-LateB-LateB-CC-NBD-ARC1-ARC2-X-LRR	Putative disease resistance protein
M1CEK5	PGSC0003DMG400025611	XCNL	2508 aa	X-LateB-X-CC-CC-NBD-ARC1-ARC2-LRR	Disease resistance protein
M1CCM0	PGSC0003DMG400025099	XCNL	1733 aa	X-LateB-X-CC-NBD-ARC1-ARC2-LRR	PRF
M1DAI3	PGSC0003DMG400035467	XN	268 aa	KIN-NBD-ARC1	Disease resistance protein RGA2
M1CEL4	PGSC0003DMG400025615	XN	591 aa	X-ARC1-ARC2-X	Disease resistance protein
M1AV64	PGSC0003DMG400011907	XN	315 aa	X-NBD	NBS-coding resistance gene analog
M1C3W2	PGSC0003DMG400022980	XN	352 aa	X-NBD	Hydrolase
M1A743	PGSC0003DMG400006297	XN	502 aa	X-NBD	Tospovirus resistance protein C
M1BMV6	PGSC0003DMG400018954	XN	553 aa	X-NBD-ARC1-ARC2	Late blight resistance protein Rpi-blb2
M0ZQH2	PGSC0003DMG400002276	XN	516 aa	X-NBD-ARC1-ARC2	NB-ARC domain-containing protein
M0ZWT4	PGSC0003DMG400003794	XNL	553 aa	X-LRR-NBD-X	Tospovirus resistance protein E
M1ADV3	PGSC0003DMG400007999	XNL	733 aa	X-NBD-ARC1-ARC2-LRR	Nbs-LRR resistance protein

M0ZQH1	PGSC0003DMG400002275	XNL	967 aa	X-NBD-ARC1-ARC2-LRR	CC-NBS-LRR resistance protein
M1BV06	PGSC0003DMG400020741	XNL	831 aa	X-NBD-ARC1-ARC2-LRR	Late blight resistance protein Rpi-blb2
M1ABJ9	PGSC0003DMG400007385	XNL	1031 aa	X-NBD-ARC1-ARC2-LRR	CC-NB-LRR protein
M1AQX0	PGSC0003DMG400010895	XNL	980 aa	X-NBD-ARC1-ARC2-LRR	Late blight resistance protein Rpi-blb2
Q75VK7	PGSC0003DMG400007385	XNL	1036 aa	X-NBD-ARC1-ARC2-LRR	CC-NB-LRR protein
M1CGS3	PGSC0003DMG400026104	XTN	486 aa	X-TIR-NBD	NBS-coding resistance gene protein
M1AWX8	PGSC0003DMG400012335	XTN	997 aa	X-TIR-X-NBD-X	TIR-NBS resistance protein
M0ZJY0	PGSC0003DMG400000888	XTNL	1369 aa	NBD-X-TIR-NBD-ARC1-ARC2-LRR-X	Resistance gene
M0ZWU1	PGSC0003DMG400003800	LN	421 aa	LRR-NBD	Hero resistance protein
M1CI76	PGSC0003DMG402026432	LXN	649 aa	LRR-X-NBD-ARC1	NBS-coding resistance gene protein
M1ADV1	PGSC0003DMG400007999	RN	233 aa	RPW8-NBD	Nbs-LRR resistance protein
M1ADV2	PGSC0003DMG400007999	RNL	832 aa	RPW8-NBD-ARC1-ARC2-LRR	Nbs-LRR resistance protein
F2YDV0	PGSC0003DMG400007999	RNL	832 aa	RPW8-NBD-ARC1-ARC2-LRR	ADR1

Table S2. Correspondence between PGSC gene IDs and the 642 UniProt protein entries listed in Supplementary Table S1. Of the 642 UniProt protein entries listed in Supplementary Table S1, 598 are associated with 427 distinct PGSC gene IDs, whereas 44 have no identified PGSC gene ID. Protein entries sharing the same PGSC ID are grouped in a single row, with UniProt accessions separated by semicolons. The 44 entries without an identified PGSC ID are grouped under "Unknown", which is excluded from the count of 427 distinct PGSC gene IDs. "No." denotes the sequential row number. NLR subclasses are listed without repetition within each group, and "Protein entry count" indicates the number of protein entries in that group.

No.	PGSC ID	UniProt ID	NLR subclass	Protein entry count
1	PGSC0003DMG400010946	M1AR37	CN	1 protein entry
2	PGSC0003DMG402002917	M0ZT66	CN	1 protein entry
3	PGSC0003DMG400021477	M1BXR3; M1BXR2	CN; CNL	2 protein entries
4	PGSC0003DMG401008383	M1AFE8	CN	1 protein entry
5	PGSC0003DMG401011522	M1ATP8	CN	1 protein entry
6	PGSC0003DMG400006576	M1A868	CN	1 protein entry
7	PGSC0003DMG401011117	M1ARU9	CN	1 protein entry
8	PGSC0003DMG400011898	M1AV54; M1AV53; Q5YEK7; Q5YEK5; Q5YEK9	CN; CNLX; N	5 protein entries
9	PGSC0003DMG400020749	M1BV14	CN	1 protein entry
10	PGSC0003DMG400020586	M1BUB7	CN	1 protein entry
11	PGSC0003DMG401026842	M1CJT1; M1CJS7	CN	2 protein entries
12	PGSC0003DMG401011526	M1ATQ2	CN	1 protein entry
13	PGSC0003DMG400021862	M1BZ78; M1BZ79	CN; NL	2 protein entries
14	PGSC0003DMG401027371	M1CM40; Q38MI2; Q38MD1; Q38MD6	CN; N	4 protein entries
15	PGSC0003DMG400007608	M1ACF6	CN	1 protein entry
16	PGSC0003DMG400007605	M1ACF4	CN	1 protein entry
17	PGSC0003DMG401002242	M0ZQC7	CN	1 protein entry
18	PGSC0003DMG400027867	M1CP25	CN	1 protein entry
19	PGSC0003DMG402026842	M1CJS8	CN	1 protein entry
20	PGSC0003DMG400016628	M1BDR2	CN	1 protein entry
21	PGSC0003DMG402015877	M1BAT9; M1BAT6	CN; XCN	2 protein entries
22	PGSC0003DMG400043650	M1DTD1	CN	1 protein entry
23	PGSC0003DMG400001994	M0ZPE2	CN	1 protein entry
24	PGSC0003DMG400032576	M1D626	CN	1 protein entry
25	PGSC0003DMG400030044	M1CY33	CN	1 protein entry
26	PGSC0003DMG400001990	M0ZPD9; M0ZPD8	CN	2 protein entries
27	PGSC0003DMG400011115	M1ARU2	CN	1 protein entry
28	PGSC0003DMG400032578	M1D627	CN	1 protein entry
29	PGSC0003DMG402030883	M1D1N7; M1D1N4	CN; CNL	2 protein entries
30	PGSC0003DMG400002947	M0ZTB6	CN	1 protein entry
31	PGSC0003DMG400026573	M1CIW2	CN	1 protein entry

32	PGSC0003DMG400018572	M1BLE7; Q38MD5; Q38MD2; Q38ME5; Q38MG6; M1BLE8	CN; N; NL	6 protein entries
33	PGSC0003DMG400019804	M1BR55	CN	1 protein entry
34	PGSC0003DMG400024429	M1C9I7	CN	1 protein entry
35	PGSC0003DMG400023250	M1C4R2	CN	1 protein entry
36	PGSC0003DMG402017089	M1BFI8	CN	1 protein entry
37	PGSC0003DMG400019900	M1BRH5	CN	1 protein entry
38	PGSC0003DMG400011109	M1ART7	CN	1 protein entry
39	PGSC0003DMG400032572	M1D623	CN	1 protein entry
40	PGSC0003DMG402009818	M1ALF3; M1ALF4	CN	2 protein entries
41	PGSC0003DMG400002820	M0ZST8	CN	1 protein entry
42	PGSC0003DMG400016823	M1BEG3	CN	1 protein entry
43	PGSC0003DMG400015872	M1BAS7; M1BAS6	CN; CNL	2 protein entries
44	PGSC0003DMG400023273	M1C4T6; Q5YEQ3	CN; N	2 protein entries
45	PGSC0003DMG400013165	M1B0D2	CN	1 protein entry
46	PGSC0003DMG400008449	M1AFN9	CN	1 protein entry
47	PGSC0003DMG400024365	M1C9B1	CN	1 protein entry
48	PGSC0003DMG400032582	M1D630	CN	1 protein entry
49	PGSC0003DMG400024231	M1C8T7	CN	1 protein entry
50	PGSC0003DMG400030239	M1CYU5	CN	1 protein entry
51	PGSC0003DMG401009818	M1ALF1; M1ALF0	CN; CNL	2 protein entries
52	PGSC0003DMG402032547	M1D5Z1	CN	1 protein entry
53	PGSC0003DMG400002960	M0ZTC8	CN	1 protein entry
54	PGSC0003DMG400014047	M1B3P2	CN	1 protein entry
55	PGSC0003DMG400007469	M1ABX5; Q5YEM2; Q5YEK2; Q5YEQ6; Q5YEL9; M1ABX4; Q5YEK3	CN; N	7 protein entries
56	PGSC0003DMG400019799	M1BR49	CN	1 protein entry
57	PGSC0003DMG400010886	M1AQV6; M1AQV7	CN; N	2 protein entries
58	PGSC0003DMG401010943	M1AR21	CN	1 protein entry
59	PGSC0003DMG400011521	M1ATP4; M1ATP5	CN; N	2 protein entries
60	PGSC0003DMG400018693	M1BLZ2	CN	1 protein entry
61	PGSC0003DMG400022785	M1C319	CN	1 protein entry
62	PGSC0003DMG401022784	M1C317	CNL	1 protein entry
63	PGSC0003DMG401011427	M1ATB1	CNL	1 protein entry
64	PGSC0003DMG400028001	M1CPJ0; M1CPJ2	CNL	2 protein entries
65	PGSC0003DMG402007871	Q9LKQ0; Q9S7Q4; Q9XGF5; M1ADC5; M1ADC4; B0FWN8; B0FWN7	CNL	7 protein entries
66	PGSC0003DMG401026043	M1CGI2; M1CGH8	CNL	2 protein entries
67	PGSC0003DMG401018576	Q5I6L7; M1BLF2; Q5I6L6; Q5I6L8; Q5I6L5	CNL	5 protein entries
68	PGSC0003DMG400017060	M1BFF0; Q6TAF9	CNL	2 protein entries
69	PGSC0003DMG400025259	M1CD99	CNL	1 protein entry

70	PGSC0003DMG400009455	M1AK50	CNL	1 protein entry
71	PGSC0003DMG400012466	M1AXG0; M1AXG1	CNL; NL	2 protein entries
72	PGSC0003DMG400006531	M1A821	CNL	1 protein entry
73	PGSC0003DMG401009305	M1AJE1	CNL	1 protein entry
74	PGSC0003DMG400019913	M1BRJ1	CNL	1 protein entry
75	PGSC0003DMG400011517	M1ATN9	CNL	1 protein entry
76	PGSC0003DMG400003527	M0ZVK0	CNL	1 protein entry
77	PGSC0003DMG400012918	M1AZF6; M1AZF4	CNL	2 protein entries
78	PGSC0003DMG400026161	M1CH05	CNL	1 protein entry
79	PGSC0003DMG400019741	M1BQY3; Q60CX9	CNL; NL	2 protein entries
80	PGSC0003DMG400018720	A6YP83	CNL	1 protein entry
81	PGSC0003DMG400007462	M1ABW0; M1ABW3	CNL; NL	2 protein entries
82	PGSC0003DMG400006785	M1A8Z3	CNL	1 protein entry
83	PGSC0003DMG400025511	M1CEA6	CNL	1 protein entry
84	PGSC0003DMG400019627	M1BQE7	CNL	1 protein entry
85	PGSC0003DMG400019418	M1BPN0	CNL	1 protein entry
86	PGSC0003DMG401015877	M1BAT8	CNL	1 protein entry
87	PGSC0003DMG400021861	M1BZ76	CNL	1 protein entry
88	PGSC0003DMG400012471	M1AXG7	CNL	1 protein entry
89	PGSC0003DMG400017146	M1BFQ3	CNL	1 protein entry
90	PGSC0003DMG400018903	M1BMQ8	CNL	1 protein entry
91	PGSC0003DMG401027402	M1CM95; A6YP89	CNL	2 protein entries
92	PGSC0003DMG401007575	M1ACA7	CNL	1 protein entry
93	PGSC0003DMG400024363	M1C9A9	CNL	1 protein entry
94	PGSC0003DMG400020587	M1BUC0	CNL	1 protein entry
95	PGSC0003DMG401019148	M1BNH5	CNL	1 protein entry
96	PGSC0003DMG400007872	M1ADD0; M1ADC9	CNL	2 protein entries
97	PGSC0003DMG400030498	M1CZW8	CNL	1 protein entry
98	PGSC0003DMG400007867	M1ADB8	CNL	1 protein entry
99	PGSC0003DMG400029405	M1CVH0	CNL	1 protein entry
100	PGSC0003DMG400009324	M1AJH8	CNL	1 protein entry
101	PGSC0003DMG400011520	M1ATP3	CNL	1 protein entry
102	PGSC0003DMG400008292	M1AF10	CNL	1 protein entry
103	PGSC0003DMG400004874	M1A1A6	CNL	1 protein entry
104	PGSC0003DMG401008349	M1AF85	CNL	1 protein entry
105	PGSC0003DMG400031405	M1D3S7; M1D3S8	CNL	2 protein entries
106	PGSC0003DMG400013891	M1B383	CNL	1 protein entry
107	PGSC0003DMG402001979	M0ZPB9	CNL	1 protein entry
108	PGSC0003DMG400001983	M0ZPC4	CNL	1 protein entry
109	PGSC0003DMG400013091	M1B035; M1B036	CNL	2 protein entries
110	PGSC0003DMG400011900	M1AV57	CNL	1 protein entry
111	PGSC0003DMG400018464	M1BKW7; M1BKW5; M1BKW6; M1BKW8	CNL	4 protein entries
112	PGSC0003DMG400029505	M1CVW1	CNL	1 protein entry

113	PGSC0003DMG400023288	M1C4V3	CNL	1 protein entry
114	PGSC0003DMG400011523	M1ATP9	CNL	1 protein entry
115	PGSC0003DMG401001984	M0ZPC5	CNL	1 protein entry
116	PGSC0003DMG400017062	M1BFF2	CNL	1 protein entry
117	PGSC0003DMG400026187	M1CH50; A0A0S1LG10	CNL; N	2 protein entries
118	PGSC0003DMG400024203	M1C8M9; M1C8M8	CNL	2 protein entries
119	PGSC0003DMG400007870	M1ADC2	CNL	1 protein entry
120	PGSC0003DMG400011529	M1ATQ6	CNL	1 protein entry
121	PGSC0003DMG400044423	M1DV09	CNL	1 protein entry
122	PGSC0003DMG400006769	M1A8W6; M1A8W7	CNL	2 protein entries
123	PGSC0003DMG400037159	M1DE52	CNL	1 protein entry
124	PGSC0003DMG400024337	M1C982	CNL	1 protein entry
125	PGSC0003DMG400018462	M1BKV8; M1BKW0; M1BKV9; M1BKW1; M1BKW3; M1BKW2	CNL	6 protein entries
126	PGSC0003DMG400008306	M1AF26	CNL	1 protein entry
127	PGSC0003DMG400006533	M1A823	CNL	1 protein entry
128	PGSC0003DMG400005046	M1A225	CNL	1 protein entry
129	PGSC0003DMG400011920	M1AV76	CNL	1 protein entry
130	PGSC0003DMG400027407	A6YP88; A6YP87; M1CMA4; A6YP82	CNL	4 protein entries
131	PGSC0003DMG400002920	M0ZT73; M0ZT72	CNL	2 protein entries
132	PGSC0003DMG400024202	M1C8M7	CNL	1 protein entry
133	PGSC0003DMG400023645	M1C6F8	CNL	1 protein entry
134	PGSC0003DMG401018696	M1BLZ6	CNL	1 protein entry
135	PGSC0003DMG400001081	M0ZKN3	CNL	1 protein entry
136	PGSC0003DMG400021948	M1BZI1	CNL	1 protein entry
137	PGSC0003DMG400019083	M1BN91	CNL	1 protein entry
138	PGSC0003DMG400038020	M1DG00	CNL	1 protein entry
139	PGSC0003DMG400018490	M1BL12; M1BL13; M1BL11	CNL; NL	3 protein entries
140	PGSC0003DMG401005337	M1A354	CNL	1 protein entry
141	PGSC0003DMG400011525	M1ATQ1; A0A1Z2X6Y5	CNL	2 protein entries
142	PGSC0003DMG400011045	M1ARI8	CNL	1 protein entry
143	PGSC0003DMG400008393	M1AFF9	CNL	1 protein entry
144	PGSC0003DMG402020585	M1BUB4	CNL	1 protein entry
145	PGSC0003DMG401017416	M1BGU8	CNL	1 protein entry
146	PGSC0003DMG400030497	M1CZW7	CNL	1 protein entry
147	PGSC0003DMG402004578	M0ZZX0; M0ZZW9	CNL	2 protein entries
148	PGSC0003DMG400020584	M1BUB2; A1YUW8	CNL	2 protein entries
149	PGSC0003DMG400021863	M1BZ80	CNL	1 protein entry
150	PGSC0003DMG400007344	M1ABE4	CNL	1 protein entry
151	PGSC0003DMG400019732	Q60CX8; M1BQW9; M1BQX0	CNL	3 protein entries
152	PGSC0003DMG400033651	M1D7C1	CNL	1 protein entry
153	PGSC0003DMG400011924	M1AV79	CNL	1 protein entry
154	PGSC0003DMG400018050	M1BJ65	CNL	1 protein entry

155	PGSC0003DMG400011926	M1AV80	CNL	1 protein entry
156	PGSC0003DMG400027377	A6YP90; M1CM52; M1CM53; Q38MF4; P93572	CNL; N	5 protein entries
157	PGSC0003DMG400019669	M1BQM1	CNL	1 protein entry
158	PGSC0003DMG401016933	M1BEY0; M1BEY2; M1BEY4	CNL; CNLX	3 protein entries
159	PGSC0003DMG400011527	M1ATQ4	CNL	1 protein entry
160	PGSC0003DMG404026043	M1CGI1	CNL	1 protein entry
161	PGSC0003DMG401030883	M1D1N3; M1D1N6; Q38MC3; Q38MI8	CNL; N	4 protein entries
162	PGSC0003DMG402017416	M1BGV0	CNL	1 protein entry
163	PGSC0003DMG400041458	M1DNM0	CNL	1 protein entry
164	PGSC0003DMG400011528	M1ATQ5	CNL	1 protein entry
165	PGSC0003DMG400002980	M0ZTE2	CNL	1 protein entry
166	PGSC0003DMG400008588	M1AG91	CNL	1 protein entry
167	PGSC0003DMG400021098	M1BW62	CNL	1 protein entry
168	PGSC0003DMG401020585	M1BUB3	CNL	1 protein entry
169	PGSC0003DMG401007461	M1ABV9	CNL	1 protein entry
170	PGSC0003DMG400006800	M1A918; M1A919	CNL; NL	2 protein entries
171	PGSC0003DMG400013721	M1B2N3	CNL	1 protein entry
172	PGSC0003DMG400030855	M1D1J6; Q6TAF8	CNL	2 protein entries
173	PGSC0003DMG400026666	M1CJ87; M1CJ86	CNL; CNX	2 protein entries
174	PGSC0003DMG400012472	M1AXG9	CNL	1 protein entry
175	PGSC0003DMG400020736	M1BV01	CNL	1 protein entry
176	PGSC0003DMG400019740	M1BQY0	CNL	1 protein entry
177	PGSC0003DMG400005052	M1A232	CNL	1 protein entry
178	PGSC0003DMG400016372	M1BCN1	CNL	1 protein entry
179	PGSC0003DMG400010612	M1APS6	CNL	1 protein entry
180	PGSC0003DMG400012470	M1AXG5; M1AXG6	CNL; NL	2 protein entries
181	PGSC0003DMG400004296	M0ZYV3	CNL	1 protein entry
182	PGSC0003DMG400008394	M1AFG0; M1AFG4; M1AFG2	CNL	3 protein entries
183	PGSC0003DMG401004423	M0ZZC5; M0ZZC6	CNL	2 protein entries
184	PGSC0003DMG400008595	M1AGA2	CNL	1 protein entry
185	PGSC0003DMG400008596	M1AGA5; M1AGA4	CNL	2 protein entries
186	PGSC0003DMG400029344	M1CV69	CNL	1 protein entry
187	PGSC0003DMG400029586	M1CW59	CNL	1 protein entry
188	PGSC0003DMG400024430	M1C9I8	CNL	1 protein entry
189	PGSC0003DMG400001996	M0ZPE3	CNL	1 protein entry
190	PGSC0003DMG400042937	M1DRT6	CNL	1 protein entry
191	PGSC0003DMG401003025	M0ZTK9; M0ZTK5	CNL; CNX	2 protein entries
192	PGSC0003DMG400013889	M1B376; H6U2T9; Q5YEK8	CNLX; N	3 protein entries
193	PGSC0003DMG400003104	M0ZTX7	CNLX	1 protein entry
194	PGSC0003DMG400004413	M0ZZA8	CNLX	1 protein entry
195	PGSC0003DMG400011906	M1AV63	CNLX	1 protein entry
196	PGSC0003DMG400011809	M1AUU6	CNLX	1 protein entry

197	PGSC0003DMG400029314	M1CV20	CNLX	1 protein entry
198	PGSC0003DMG400001981	M0ZPC2	CNX	1 protein entry
199	PGSC0003DMG400046201	M1DYW3	CNX	1 protein entry
200	PGSC0003DMG400029313	M1CV19	CNX	1 protein entry
201	PGSC0003DMG400043400	M1DST2	CXNL	1 protein entry
202	PGSC0003DMG400022087	M1BZW6	N	1 protein entry
203	PGSC0003DMG400004523	M0ZZN4	N	1 protein entry
204	PGSC0003DMG401011899	M1AV55	N	1 protein entry
205	PGSC0003DMG400018573	M1BLE9	N	1 protein entry
206	PGSC0003DMG402020581	M1BUA9; M1BUA7	N; NL	2 protein entries
207	PGSC0003DMG400028056	M1CPQ2	N	1 protein entry
208	PGSC0003DMG401030700	M1D0W6; M1D0W5; M1D0W4	N; NLX; TNLX	3 protein entries
209	PGSC0003DMG402027371	M1CM41; M1CM42	N	2 protein entries
210	PGSC0003DMG400033160	M1D6U1	N	1 protein entry
211	PGSC0003DMG400003020	M0ZTK3	N	1 protein entry
212	PGSC0003DMG400032581	M1D629	N	1 protein entry
213	PGSC0003DMG400026104	Q9FQ18; P93578; A0A0S1LG15; M1CGS3	N; XTN	4 protein entries
214	PGSC0003DMG400004303	M0ZYW2	N	1 protein entry
215	PGSC0003DMG400011193	M1AS82	N	1 protein entry
216	PGSC0003DMG400046190	M1DYV5	N	1 protein entry
217	PGSC0003DMG400030267	M1CYY1	N	1 protein entry
218	PGSC0003DMG400032580	M1D628	N	1 protein entry
219	PGSC0003DMG400024234	M1C8U1	N	1 protein entry
220	PGSC0003DMG400015031	M1B7J3; P93575; P93576; M1B7J2	N; NX	4 protein entries
221	PGSC0003DMG400034825	M1D934	N	1 protein entry
222	PGSC0003DMG400019803	M1BR53; M1BR54	N; NL	2 protein entries
223	PGSC0003DMG400004524	M0ZZN5	N	1 protein entry
224	PGSC0003DMG400016029	M1BBA6	N	1 protein entry
225	PGSC0003DMG400017086	M1BFI2	N	1 protein entry
226	PGSC0003DMG400033151	M1D6T5	N	1 protein entry
227	PGSC0003DMG402021043	M1BW09	N	1 protein entry
228	PGSC0003DMG400027409	Q38MC5; Q38MF2; Q38ME1; Q38MG9; Q38MG4; Q38MD4; M1CMA5	N; NL	7 protein entries
229	PGSC0003DMG400009635	M1AKU1	N	1 protein entry
230	PGSC0003DMG401029961	M1CXT3	N	1 protein entry
231	PGSC0003DMG400018907	M1BMR1	N	1 protein entry
232	PGSC0003DMG400007999	A0A0S1LFZ7; A0A0S1LG04; A0A0S1LG13; A0A0S1LG24; A0A0S1LG02; M1ADV3; M1ADV1; M1ADV2; F2YDV0	N; XNL; RN; RNL	9 protein entries
233	PGSC0003DMG400023253	M1C4R3	N	1 protein entry

234	PGSC0003DMG400018046	M1BJ60	N	1 protein entry
235	PGSC0003DMG400017666	Q38MF7; Q38MF3; M1BHU9	N; XCN	3 protein entries
236	PGSC0003DMG400000815	M0ZJP0	N	1 protein entry
237	PGSC0003DMG400017061	M1BFF1	N	1 protein entry
238	PGSC0003DMG400042556	M1DR07	N	1 protein entry
239	PGSC0003DMG400004579	M0ZZX1; Q5YEQ2; Q5YEK6; Q5YEP9	N	4 protein entries
240	PGSC0003DMG400005577	Q60CW4	N	1 protein entry
241	PGSC0003DMG403016979	M1BF49	N	1 protein entry
242	PGSC0003DMG402000927	M0ZK38	N	1 protein entry
243	PGSC0003DMG400001982	M0ZPC3	N	1 protein entry
244	PGSC0003DMG400013904	M1B3A5	N	1 protein entry
245	PGSC0003DMG400024273	M1C8Z2	N	1 protein entry
246	PGSC0003DMG400025615	Q5YEN3; Q5YEM0; M1CEL4	N; XN	3 protein entries
247	PGSC0003DMG400011524	M1ATQ0	N	1 protein entry
248	PGSC0003DMG400021986	Q5YEN4; M1BZL5	N; XCN	2 protein entries
249	PGSC0003DMG402029995	M1CXY0	N	1 protein entry
250	PGSC0003DMG400032584	M1D631	N	1 protein entry
251	PGSC0003DMG400023656	M1C6G7	N	1 protein entry
252	PGSC0003DMG400033161	M1D6U2	N	1 protein entry
253	PGSC0003DMG400010944	M1AR33	N	1 protein entry
254	PGSC0003DMG400033159	M1D6U0	N	1 protein entry
255	PGSC0003DMG400029606	M1CW84	N	1 protein entry
256	PGSC0003DMG400027932	M1CPB3	N	1 protein entry
257	PGSC0003DMG400005174	M1A2J7	N	1 protein entry
258	PGSC0003DMG400002799	M0ZSQ3; M0ZSQ4; M0ZSQ5	N; NL; TNLX	3 protein entries
259	PGSC0003DMG402004425	M0ZZD2	NL	1 protein entry
260	PGSC0003DMG400002982	M0ZTE6	NL	1 protein entry
261	PGSC0003DMG401007871	M1ADC7; M1ADC3	NL; NLX	2 protein entries
262	PGSC0003DMG400030045	M1CY34	NL	1 protein entry
263	PGSC0003DMG402011117	M1ARU5	NL	1 protein entry
264	PGSC0003DMG400007869	M1ADC0; M1ADC1	NL	2 protein entries
265	PGSC0003DMG400018570	M1BLE6	NL	1 protein entry
266	PGSC0003DMG403026842	M1CJT0; M1CJS9	NL	2 protein entries
267	PGSC0003DMG402011522	M1ATP7	NL	1 protein entry
268	PGSC0003DMG400019970	M1BRP9	NL	1 protein entry
269	PGSC0003DMG400006316	M1A777	NL	1 protein entry
270	PGSC0003DMG400016656	M1BDV5	NL	1 protein entry
271	PGSC0003DMG400018463	M1BKW4	NL	1 protein entry
272	PGSC0003DMG400001992	M0ZPE0	NL	1 protein entry
273	PGSC0003DMG401007609	M1ACF7	NL	1 protein entry
274	PGSC0003DMG402008383	M1AFE7	CN	1 protein entry
275	PGSC0003DMG400009633	M1AKT2; M1AKT3	NL	2 protein entries
276	PGSC0003DMG400031521	M1D467	NL	1 protein entry

277	PGSC0003DMG400000813	M0ZJN9	NL	1 protein entry
278	PGSC0003DMG402011427	M1ATB4	NL	1 protein entry
279	PGSC0003DMG400000816	M0ZJP2	NL	1 protein entry
280	PGSC0003DMG400010287	M1ANG8	NL	1 protein entry
281	PGSC0003DMG400012650	M1AYC0	NL	1 protein entry
282	PGSC0003DMG402020874	M1BVH5	NL	1 protein entry
283	PGSC0003DMG400010953	M1AR44	NL	1 protein entry
284	PGSC0003DMG401009819	M1ALF6	NL	1 protein entry
285	PGSC0003DMG400004756	M1A0R5	NL	1 protein entry
286	PGSC0003DMG400025545	M1CEE4; A4UV23; M1CEE6; M1CEE5; A4UV52; A4UV24; A4UV39; A4UV40; A4UV25; A4UV51; A4UV53	NL; XCN; XCNL	11 protein entries
287	PGSC0003DMG400008257	M1AEV9	NL	1 protein entry
288	PGSC0003DMG400022989	M1C3X4	NL	1 protein entry
289	PGSC0003DMG400002278	M0ZQH5	NL	1 protein entry
290	PGSC0003DMG400001993	M0ZPE1	NL	1 protein entry
291	PGSC0003DMG400004295	M0ZYV2	NL	1 protein entry
292	PGSC0003DMG400001989	M0ZPD7	NL	1 protein entry
293	PGSC0003DMG402030235	M1CYT7	NL	1 protein entry
294	PGSC0003DMG400011425	M1ATA8	NL	1 protein entry
295	PGSC0003DMG400011111	M1ART8	NL	1 protein entry
296	PGSC0003DMG400007471	M1ABX6	NL	1 protein entry
297	PGSC0003DMG400013308	M1B115	NL	1 protein entry
298	PGSC0003DMG400027325	M1CLX6	NL	1 protein entry
299	PGSC0003DMG400025683	M1CEV6	NL	1 protein entry
300	PGSC0003DMG400023249	M1C4R1	NL	1 protein entry
301	PGSC0003DMG403020585	M1BUB6	NL	1 protein entry
302	PGSC0003DMG400029590	M1CW62	NL	1 protein entry
303	PGSC0003DMG400030238	M1CYU4	NL	1 protein entry
304	PGSC0003DMG402011526	M1ATQ3	NL	1 protein entry
305	PGSC0003DMG400013407	M1B1G0	NL	1 protein entry
306	PGSC0003DMG400005970	M1A5T5; M1A5T6	NL; XCNL	2 protein entries
307	PGSC0003DMG400033153	M1D6T7	NL	1 protein entry
308	PGSC0003DMG400021469	M1BXQ5	NL	1 protein entry
309	PGSC0003DMG400013736	M1B2Q1	NL	1 protein entry
310	PGSC0003DMG400002277	M0ZQH4	NL	1 protein entry
311	PGSC0003DMG402007461	M1ABV8	NL	1 protein entry
312	PGSC0003DMG400004873	M1A1A5	NL	1 protein entry
313	PGSC0003DMG400021169	M1BWI5	NL	1 protein entry
314	PGSC0003DMG400021779	M1BYW4	NL	1 protein entry
315	PGSC0003DMG400007742	M1ACW4	NL	1 protein entry
316	PGSC0003DMG400002271	M0ZQG7	NL	1 protein entry
317	PGSC0003DMG400016600	M1BDL8	NL	1 protein entry

318	PGSC0003DMG400005172	M1A2J4	NL	1 protein entry
319	PGSC0003DMG400017317	Q6XZH4; Q6XZH7; Q6XZH5; Q6XZH8; M1BGG4; Q6XZH6	NLX; TNLX	6 protein entries
320	PGSC0003DMG400016808	M1BEE2	NLX	1 protein entry
321	PGSC0003DMG402020721	M1BUY4	NLX	1 protein entry
322	PGSC0003DMG400013094	M1B039	NLX	1 protein entry
323	PGSC0003DMG400033334	M1D6X4; M1D6X6; M1D6X5	NLX; TNL; TNLX	3 protein entries
324	PGSC0003DMG400015128	M1B7T7; M1B7T6	NX; TNLX	2 protein entries
325	PGSC0003DMG403019668	M1BQM0	NX	1 protein entry
326	PGSC0003DMG402018257	M1BK11; M1BK10; M1BK09	NX; TN; TNX	3 protein entries
327	PGSC0003DMG402009305	M1AJE2	NX	1 protein entry
328	PGSC0003DMG400028081	M1CPT6	NX	1 protein entry
329	PGSC0003DMG400022004	M1BZN8	NX	1 protein entry
330	PGSC0003DMG400046545	M1DZM7	NX	1 protein entry
331	PGSC0003DMG401030236	M1CYT8	NXL	1 protein entry
332	PGSC0003DMG400033131	M1D6Q7	NXL	1 protein entry
333	PGSC0003DMG400005170	M1A2J2	NXL	1 protein entry
334	PGSC0003DMG400005173	M1A2J6	NXL	1 protein entry
335	PGSC0003DMG401015682	M1BA17; A0A0X7YK15	TN; TNLX	2 protein entries
336	PGSC0003DMG400024206	M1C8N7	TN	1 protein entry
337	PGSC0003DMG400026433	M1CI84	TN	1 protein entry
338	PGSC0003DMG402002428	Q9ZS32; M0ZR09	TN; TNL	2 protein entries
339	PGSC0003DMG400031318	M1D3I2	TN	1 protein entry
340	PGSC0003DMG400009686	M1AKZ2	TN	1 protein entry
341	PGSC0003DMG400013543	M1B208	TNL	1 protein entry
342	PGSC0003DMG403016981	M1BF54	TNL	1 protein entry
343	PGSC0003DMG400002357	M0ZQS6	TNL	1 protein entry
344	PGSC0003DMG400013090	M1B034	TNL	1 protein entry
345	PGSC0003DMG400024055	M1C837; M1C838	TNL; TNLX	2 protein entries
346	PGSC0003DMG400020722	M1BUY9; M1BUY7; M1BUY8	TNL; TNLX	3 protein entries
347	PGSC0003DMG400015681	M1BA09	TNL	1 protein entry
348	PGSC0003DMG400002426	A0A109NGX3; M0ZR05	TNL; TNLX	2 protein entries
349	PGSC0003DMG400010527	M1APH0	TNL	1 protein entry
350	PGSC0003DMG400021887	Q9ZS31; M1BZB1	TNL; TNLX	2 protein entries
351	PGSC0003DMG400003445	M0ZV73	TNL	1 protein entry
352	PGSC0003DMG402016979	M1BF44	TNL	1 protein entry
353	PGSC0003DMG401026432	M1CI75	TNL	1 protein entry
354	PGSC0003DMG400006003	M1A5Y9; M1A5Z0	TNL; TNLX	2 protein entries
355	PGSC0003DMG400007743	M1ACW5	TNLX	1 protein entry
356	PGSC0003DMG400002427	M0ZR07	TNLX	1 protein entry
357	PGSC0003DMG400013098	M1B044; M1B043	TNLX	2 protein entries
358	PGSC0003DMG400022699	M1C2N4	TNLX	1 protein entry
359	PGSC0003DMG402016981	M1BF53; A0A0X7YE88	TNLX	2 protein entries
360	PGSC0003DMG400027797	M1CNT1	TNLX	1 protein entry

361	PGSC0003DMG400020935	M1BVN8	TNLX	1 protein entry
362	PGSC0003DMG401020721	M1BUY5	TNLX	1 protein entry
363	PGSC0003DMG400018428	M1BKQ7	TNLX	1 protein entry
364	PGSC0003DMG400018461	M1BKV7	TNLX	1 protein entry
365	PGSC0003DMG400018429	M1BKQ8	TNLX	1 protein entry
366	PGSC0003DMG400013627	M1B2A0	TNLX	1 protein entry
367	PGSC0003DMG401022603	M1C298	TNLX	1 protein entry
368	PGSC0003DMG400018256	M1BK03; M1BK04	TNLX	2 protein entries
369	PGSC0003DMG400018216	M1BJT4	TNLX	1 protein entry
370	PGSC0003DMG400029415	M1CVI4	TNLX	1 protein entry
371	PGSC0003DMG400015693	M1BA43	TNX	1 protein entry
372	PGSC0003DMG401020581	M1BUA6	TTN	1 protein entry
373	PGSC0003DMG400044837	M1DVX2	TTN	1 protein entry
374	PGSC0003DMG400006570	M1A861	XCN	1 protein entry
375	PGSC0003DMG400020851	M1BVE8	XCN	1 protein entry
376	PGSC0003DMG400029506	M1CVW2	XCN	1 protein entry
377	PGSC0003DMG400003353	M0ZUW1	XCN	1 protein entry
378	PGSC0003DMG400038440	M1DGX1	XCN	1 protein entry
379	PGSC0003DMG400043186	M1DSB9	XCN	1 protein entry
380	PGSC0003DMG401016602	M1BDM2	XCN	1 protein entry
381	PGSC0003DMG400020740	M1BV05	XCN	1 protein entry
382	PGSC0003DMG400013490	M1B1S9; M1B1T0	XCN; XCNL	2 protein entries
383	PGSC0003DMG401010614	M1APS8	XCN	1 protein entry
384	PGSC0003DMG400031185	M1D2Z2	XCN	1 protein entry
385	PGSC0003DMG401013522	M1B1Y2	XCN	1 protein entry
386	PGSC0003DMG400025099	M1CCL8; M1CCM0	XCN; XCNL	2 protein entries
387	PGSC0003DMG400003380	M0ZUZ5	XCNL	1 protein entry
388	PGSC0003DMG400018441	M1BKT0	XCNL	1 protein entry
389	PGSC0003DMG400023062	M1C460; M1C459; A4UV41	XCNL	3 protein entries
390	PGSC0003DMG400009307	M1AJE5	XCNL	1 protein entry
391	PGSC0003DMG400029460	M1CVN6	XCNL	1 protein entry
392	PGSC0003DMG400013486	M1B1S4	XCNL	1 protein entry
393	PGSC0003DMG400029457	M1CVN5	XCNL	1 protein entry
394	PGSC0003DMG400031878	M1D4W2	XCNL	1 protein entry
395	PGSC0003DMG400004561	M0ZZS9	XCNL	1 protein entry
396	PGSC0003DMG400002217	M0ZQ85	XCNL	1 protein entry
397	PGSC0003DMG400006573	M1A865; M1A864	XCNL	2 protein entries
398	PGSC0003DMG400029504	M1CVW0	XCNL	1 protein entry
399	PGSC0003DMG400025512	M1CEA7	XCNL	1 protein entry
400	PGSC0003DMG400020735	M1BV00	XCNL	1 protein entry
401	PGSC0003DMG400029453	M1CVN3	XCNL	1 protein entry
402	PGSC0003DMG400029462	M1CVN8	XCNL	1 protein entry
403	PGSC0003DMG400018619	M1BLL8	XCNL	1 protein entry
404	PGSC0003DMG400020732	M1BUZ9	XCNL	1 protein entry

405	PGSC0003DMG400020872	M1BVH3	XCNL	1 protein entry
406	PGSC0003DMG402006288	M1A730	XCNL	1 protein entry
407	PGSC0003DMG400016601	M1BDM1	XCNL	1 protein entry
408	PGSC0003DMG402018576	M1BLF3	XCNL	1 protein entry
409	PGSC0003DMG400028339	M1CR23; M1CR22	XCNL	2 protein entries
410	PGSC0003DMG400013506	M1B1V4	XCNL	1 protein entry
411	PGSC0003DMG400018442	M1BKT1	XCNL	1 protein entry
412	PGSC0003DMG400025611	M1CEK5	XCNL	1 protein entry
413	PGSC0003DMG400035467	M1DAI3	XN	1 protein entry
414	PGSC0003DMG400011907	M1AV64	XN	1 protein entry
415	PGSC0003DMG400022980	M1C3W2	XN	1 protein entry
416	PGSC0003DMG400006297	M1A743	XN	1 protein entry
417	PGSC0003DMG400018954	M1BMV6	XN	1 protein entry
418	PGSC0003DMG400002276	M0ZQH2	XN	1 protein entry
419	PGSC0003DMG400003794	M0ZWT4	XNL	1 protein entry
420	PGSC0003DMG400002275	M0ZQH1	XNL	1 protein entry
421	PGSC0003DMG400020741	M1BV06	XNL	1 protein entry
422	PGSC0003DMG400007385	M1ABJ9; Q75VK7	XNL	2 protein entries
423	PGSC0003DMG400010895	M1AQX0	XNL	1 protein entry
424	PGSC0003DMG400012335	M1AWX8	XTN	1 protein entry
425	PGSC0003DMG400000888	M0ZJY0	XTNL	1 protein entry
426	PGSC0003DMG400003800	M0ZWU1	LN	1 protein entry
427	PGSC0003DMG402026432	M1CI76	LXN	1 protein entry
428	Unknown	A0A411D3A0; A0A6M5WSJ6; P93579; P93577; Q38MH5; A0A410HXX5; P93573; P93581; P93580; P93582; A0A451G5H9; P93583; Q9XGF6; Q38MI4; Q38MC2; Q38MF1; Q38MH7; Q38MH6; Q38MF0; Q38MG2; Q38MC4; Q38MG8; Q38MI0; Q38MG5; Q38MJ1; Q38MI1; Q38MC7; Q38MI9; Q38MG3; Q38MF6; Q38MH0; Q5YEN6; Q5YEN7; Q5YEM3; Q5YEM6; H6U2T8; Q5YEM7; Q5YEM1; Q5YEN5; Q5YEM8; Q5YEK4; Q5YEQ4; Q93YA7; Q93YA6	CN; CNL; N; TNLX	44 protein entries

Table S3. 193 selected potato NLR protein entries used for phylogenetic, *cis*-element, and transcriptomic analyses. UniProt ID, UniProt accession of each selected protein; PGSC gene ID, corresponding potato genome gene identifier; Subtype, NLR subtype assigned according to domain composition; Promoter available, availability of the corresponding 2-kb upstream sequence for PlantCARE *cis*-element analysis; RNA-seq available, availability of corresponding expression data in the PVY$^{N\text{-}Wilga}$ transcriptome dataset. "Yes" indicates that the corresponding data were available and included in the analysis, whereas "No" indicates that the relevant promoter sequence or transcriptome data were unavailable.

Uniprot ID	PGSC ID	Subtype	*Cis*-element_availability	RNA seq_availability
M1AV53	PGSC0003DMG400011898	CNLX	Yes	Yes
M0ZZA8	PGSC0003DMG400004413	CNLX	Yes	Yes
M1BEY2	PGSC0003DMG401016933	CNLX	Yes	Yes
M1BEY4	PGSC0003DMG401016933	CNLX	Yes	Yes
M0ZTX7	PGSC0003DMG400003104	CNLX	Yes	No
M1AV63	PGSC0003DMG400011906	CNLX	Yes	Yes
M1AUU6	PGSC0003DMG400011809	CNLX	Yes	Yes
M1CV20	PGSC0003DMG400029314	CNLX	Yes	Yes
M1B376	PGSC0003DMG400013889	CNLX	Yes	No
M1CJ86	PGSC0003DMG400026666	CNX	Yes	Yes
M1DYW3	PGSC0003DMG400046201	CNX	Yes	Yes
M0ZPC2	PGSC0003DMG400001981	CNX	Yes	Yes
M0ZTK5	PGSC0003DMG401003025	CNX	Yes	Yes
M1CV19	PGSC0003DMG400029313	CNX	Yes	Yes
M1DST2	PGSC0003DMG400043400	CXNL	Yes	Yes
M1CD99	PGSC0003DMG400025259	CNL	Yes	Yes
M1AK50	PGSC0003DMG400009455	CNL	Yes	Yes
M1AXG0	PGSC0003DMG400012466	CNL	Yes	Yes
M1A821	PGSC0003DMG400006531	CNL	Yes	No
M1AJE1	PGSC0003DMG401009305	CNL	Yes	Yes
M1BRJ1	PGSC0003DMG400019913	CNL	Yes	Yes
M1ATN9	PGSC0003DMG400011517	CNL	Yes	Yes
M1CPJ2	PGSC0003DMG400028001	CNL	Yes	Yes
M0ZVK0	PGSC0003DMG400003527	CNL	Yes	Yes
M1CH05	PGSC0003DMG400026161	CNL	Yes	Yes
M1D5Z1	PGSC0003DMG402032547	CN	Yes	Yes
M0ZST8	PGSC0003DMG400002820	CN	Yes	Yes
M1BEG3	PGSC0003DMG400016823	CN	Yes	Yes
M1C4T6	PGSC0003DMG400023273	CN	Yes	Yes
M1B0D2	PGSC0003DMG400013165	CN	Yes	Yes
M1AFN9	PGSC0003DMG400008449	CN	Yes	Yes
M1C9B1	PGSC0003DMG400024365	CN	Yes	Yes
M1D630	PGSC0003DMG400032582	CN	Yes	Yes
M1C8T7	PGSC0003DMG400024231	CN	Yes	Yes

M1CYU5	PGSC0003DMG400030239	CN	Yes	Yes
M1BQM0	PGSC0003DMG403019668	NX	Yes	Yes
M1BK11	PGSC0003DMG402018257	NX	No	No
M1B7T7	PGSC0003DMG400015128	NX	No	No
M1AJE2	PGSC0003DMG402009305	NX	Yes	Yes
M1DZM7	PGSC0003DMG400046545	NX	Yes	Yes
M1CPT6	PGSC0003DMG400028081	NX	No	Yes
M1B7J2	PGSC0003DMG400015031	NX	Yes	Yes
M1BZN8	PGSC0003DMG400022004	NX	Yes	Yes
Q6XZH4	PGSC0003DMG400017317	NLX	No	Yes
M1BEE2	PGSC0003DMG400016808	NLX	Yes	Yes
M1ADC3	PGSC0003DMG401007871	NLX	No	Yes
M1BUY4	PGSC0003DMG402020721	NLX	Yes	No
M1B039	PGSC0003DMG400013094	NLX	Yes	Yes
M1D6X4	PGSC0003DMG400033334	NLX	No	Yes
M1CYT8	PGSC0003DMG401030236	NXL	Yes	Yes
M1D6Q7	PGSC0003DMG400033131	NXL	Yes	Yes
M1A2J2	PGSC0003DMG400005170	NXL	Yes	Yes
M1A2J6	PGSC0003DMG400005173	NXL	Yes	Yes
Q60CW4	PGSC0003DMG400005577	N	Yes	Yes
M1BF49	PGSC0003DMG403016979	N	No	No
M0ZK38	PGSC0003DMG402000927	N	Yes	Yes
M1AQV7	PGSC0003DMG400010886	N	No	Yes
M0ZPC3	PGSC0003DMG400001982	N	Yes	Yes
M1CXY0	PGSC0003DMG402029995	N	Yes	Yes
M1CW84	PGSC0003DMG400029606	N	Yes	Yes
M1C6G7	PGSC0003DMG400023656	N	Yes	Yes
M1ATQ0	PGSC0003DMG400011524	N	Yes	Yes
M1CPB3	PGSC0003DMG400027932	N	No	No
M1D467	PGSC0003DMG400031521	NL	Yes	Yes
M0ZJP2	PGSC0003DMG400000816	NL	Yes	Yes
M1BVH5	PGSC0003DMG402020874	NL	No	No
M1AR44	PGSC0003DMG400010953	NL	No	No
M1D6T7	PGSC0003DMG400033153	NL	Yes	Yes
M1CYT7	PGSC0003DMG402030235	NL	Yes	No
M1BUB6	PGSC0003DMG403020585	NL	Yes	Yes
M1CW62	PGSC0003DMG400029590	NL	Yes	Yes
M1ATQ3	PGSC0003DMG402011526	NL	Yes	Yes
M1ABW3	PGSC0003DMG400007462	NL	Yes	Yes
M1BDM2	PGSC0003DMG401016602	XCN	Yes	Yes
M1B1Y2	PGSC0003DMG401013522	XCN	Yes	Yes
M1A861	PGSC0003DMG400006570	XCN	Yes	Yes
M1BVE8	PGSC0003DMG400020851	XCN	Yes	Yes
M1CVW2	PGSC0003DMG400029506	XCN	Yes	Yes

M1CEE6	PGSC0003DMG400025545	XCN	Yes	Yes
M1CCL8	PGSC0003DMG400025099	XCN	Yes	Yes
M1BZL5	PGSC0003DMG400021986	XCN	Yes	Yes
M1B1S9	PGSC0003DMG400013490	XCN	Yes	Yes
M0ZUW1	PGSC0003DMG400003353	XCN	Yes	Yes
M1D2Z2	PGSC0003DMG400031185	XCN	No	No
M1DGX1	PGSC0003DMG400038440	XCN	Yes	Yes
M1BHU9	PGSC0003DMG400017666	XCN	Yes	Yes
M1BAT6	PGSC0003DMG402015877	XCN	No	No
M1BV05	PGSC0003DMG400020740	XCN	Yes	Yes
M1DSB9	PGSC0003DMG400043186	XCN	No	No
M1APS8	PGSC0003DMG401010614	XCN	Yes	Yes
M1CVN5	PGSC0003DMG400029457	XCNL	Yes	Yes
M1CCM0	PGSC0003DMG400025099	XCNL	Yes	Yes
M1D4W2	PGSC0003DMG400031878	XCNL	Yes	Yes
M0ZZS9	PGSC0003DMG400004561	XCNL	No	Yes
M0ZUZ5	PGSC0003DMG400003380	XCNL	Yes	Yes
M1B1V4	PGSC0003DMG400013506	XCNL	Yes	Yes
M0ZQ85	PGSC0003DMG400002217	XCNL	Yes	Yes
M1BKT1	PGSC0003DMG400018442	XCNL	Yes	Yes
M1B1T0	PGSC0003DMG400013490	XCNL	Yes	Yes
M1A865	PGSC0003DMG400006573	XCNL	Yes	Yes
A4UV40	PGSC0003DMG400025545	XCNL	Yes	Yes
M1CVW0	PGSC0003DMG400029504	XCNL	Yes	Yes
M1BDM1	PGSC0003DMG400016601	XCNL	No	No
M1C459	PGSC0003DMG400023062	XCNL	Yes	Yes
A4UV41	PGSC0003DMG400023062	XCNL	Yes	Yes
M1CR22	PGSC0003DMG400028339	XCNL	Yes	Yes
M1AJE5	PGSC0003DMG400009307	XCNL	Yes	Yes
M1CEA7	PGSC0003DMG400025512	XCNL	Yes	Yes
M1BV00	PGSC0003DMG400020735	XCNL	Yes	Yes
M1CEE5	PGSC0003DMG400025545	XCNL	Yes	Yes
M1CVN3	PGSC0003DMG400029453	XCNL	Yes	Yes
M1CVN8	PGSC0003DMG400029462	XCNL	Yes	Yes
M1CEK5	PGSC0003DMG400025611	XCNL	Yes	Yes
M1A5T6	PGSC0003DMG400005970	XCNL	Yes	Yes
A4UV52	PGSC0003DMG400025545	XCNL	Yes	Yes
M1BLL8	PGSC0003DMG400018619	XCNL	Yes	Yes
A4UV25	PGSC0003DMG400025545	XCNL	Yes	Yes
M1BKT0	PGSC0003DMG400018441	XCNL	Yes	Yes
M1CVN6	PGSC0003DMG400029460	XCNL	Yes	Yes
M1C460	PGSC0003DMG400023062	XCNL	Yes	Yes
M1CR23	PGSC0003DMG400028339	XCNL	Yes	Yes
M1BUZ9	PGSC0003DMG400020732	XCNL	Yes	Yes

M1BVH3	PGSC0003DMG400020872	XCNL	No	No
M1B1S4	PGSC0003DMG400013486	XCNL	Yes	Yes
M1BLF3	PGSC0003DMG402018576	XCNL	Yes	Yes
M1A730	PGSC0003DMG402006288	XCNL	Yes	Yes
A4UV24	PGSC0003DMG400025545	XCNL	Yes	Yes
M1A864	PGSC0003DMG400006573	XCNL	Yes	Yes
A4UV53	PGSC0003DMG400025545	XCNL	Yes	Yes
A4UV51	PGSC0003DMG400025545	XCNL	Yes	Yes
A4UV39	PGSC0003DMG400025545	XCNL	Yes	Yes
M1DAI3	PGSC0003DMG400035467	XN	No	Yes
M1CEL4	PGSC0003DMG400025615	XN	Yes	No
M1AV64	PGSC0003DMG400011907	XN	Yes	Yes
M1C3W2	PGSC0003DMG400022980	XN	No	No
M1BMV6	PGSC0003DMG400018954	XN	Yes	Yes
M1A743	PGSC0003DMG400006297	XN	Yes	Yes
M0ZQH2	PGSC0003DMG400002276	XN	Yes	Yes
M1ADV3	PGSC0003DMG400007999	XNL	Yes	Yes
M0ZQH1	PGSC0003DMG400002275	XNL	Yes	Yes
M1BV06	PGSC0003DMG400020741	XNL	Yes	Yes
M1ABJ9	PGSC0003DMG400007385	XNL	Yes	Yes
M0ZWT4	PGSC0003DMG400003794	XNL	Yes	Yes
M1AQX0	PGSC0003DMG400010895	XNL	Yes	Yes
Q75VK7	PGSC0003DMG400007385	XNL	Yes	Yes
M1AWX8	PGSC0003DMG400012335	XTN	Yes	Yes
M1CGS3	PGSC0003DMG400026104	XTN	No	No
M0ZJY0	PGSC0003DMG400000888	XTNL	No	No
M1BUY7	PGSC0003DMG400020722	TNLX	Yes	Yes
M1BZB1	PGSC0003DMG400021887	TNLX	Yes	No
M0ZR07	PGSC0003DMG400002427	TNLX	Yes	Yes
M0ZSQ5	PGSC0003DMG400002799	TNLX	Yes	Yes
M1B044	PGSC0003DMG400013098	TNLX	Yes	Yes
A0A0X7YK15	PGSC0003DMG401015682	TNLX	Yes	Yes
M1B043	PGSC0003DMG400013098	TNLX	Yes	Yes
M1C2N4	PGSC0003DMG400022699	TNLX	No	No
M1BUY8	PGSC0003DMG400020722	TNLX	Yes	Yes
Q6XZH7	PGSC0003DMG400017317	TNLX	Yes	Yes
M1BK03	PGSC0003DMG400018256	TNLX	Yes	Yes
M1ACW5	PGSC0003DMG400007743	TNLX	No	No
M1BJT4	PGSC0003DMG400018216	TNLX	Yes	Yes
Q6XZH5	PGSC0003DMG400017317	TNLX	Yes	Yes
Q6XZH8	PGSC0003DMG400017317	TNLX	Yes	Yes
M1C298	PGSC0003DMG401022603	TNLX	Yes	Yes
M1BF53	PGSC0003DMG402016981	TNLX	Yes	Yes
M1C838	PGSC0003DMG400024055	TNLX	Yes	Yes

M1B7T6	PGSC0003DMG400015128	TNLX	Yes	Yes
A0A0X7YE88	PGSC0003DMG402016981	TNLX	Yes	Yes
M1A5Z0	PGSC0003DMG400006003	TNLX	Yes	Yes
M1CNT1	PGSC0003DMG400027797	TNLX	Yes	Yes
M1D0W4	PGSC0003DMG401030700	TNLX	Yes	Yes
M1CVI4	PGSC0003DMG400029415	TNLX	Yes	Yes
M1BVN8	PGSC0003DMG400020935	TNLX	Yes	Yes
M1BGG4	PGSC0003DMG400017317	TNLX	Yes	Yes
M0ZR05	PGSC0003DMG400002426	TNLX	Yes	Yes
M1BUY5	PGSC0003DMG401020721	TNLX	Yes	Yes
M1BKQ7	PGSC0003DMG400018428	TNLX	No	No
M1D6X5	PGSC0003DMG400033334	TNLX	Yes	Yes
M1BKV7	PGSC0003DMG400018461	TNLX	Yes	Yes
M1BKQ8	PGSC0003DMG400018429	TNLX	No	No
M1BK04	PGSC0003DMG400018256	TNLX	Yes	Yes
Q6XZH6	PGSC0003DMG400017317	TNLX	Yes	Yes
M1B2A0	PGSC0003DMG400013627	TNLX	Yes	Yes
Q93YA7	Unknow	TNLX	No	No
Q93YA6	Unknow	TNLX	No	No
M1BK09	PGSC0003DMG402018257	TNX	Yes	Yes
M1BA43	PGSC0003DMG400015693	TNX	Yes	Yes
M1BK10	PGSC0003DMG402018257	TN	Yes	Yes
Q9ZS32	PGSC0003DMG402002428	TN	Yes	Yes
M1D3I2	PGSC0003DMG400031318	TN	Yes	Yes
M1AKZ2	PGSC0003DMG400009686	TN	Yes	Yes
M1BF44	PGSC0003DMG402016979	TNL	Yes	Yes
M0ZR09	PGSC0003DMG402002428	TNL	Yes	Yes

Table S4. Primers used for RT-qPCR assays.

Product Name	Primer Name	Primer Sequence (5′-3′)	Product Length (bp)	Accession number
PVX	PVX-F	AAGCCTGAGCACAAATTCGC	101	NC_011620
	PVX-R	GCTTCAGACGGTGGCCG		
PVY	PVY-F	GGGTTTAGCGCGTTATGCC	150	AY884985
	PVY-R	TCTTGTGTACTGATGCCACCG		
NbPR-1	NbPR1-F	TCGTGCAGTTGTAGGCGTAG	136	Niben101Scf13926g01014.1
	NbPR1-R	CTCAGCTAGGTTTTCGCCGT		
NbPR-5	NbPR5-F	ACGTGAACCCAGGAACAGTC	105	Niben101Scf01400g00014.1
	NbPR5-R	GCATCCCGTTACAGTCTCCA		
GUS	GUS-F	ACGCTCACACCAGATACCAT	297	NC_000913.3
	GUS-R	ATACCTGTTCACCGACGACG		
NbActin	NbActin-F	CGCACCACCAGAGAGGAAAT	57	AY594294
	NbActin-R	AGGGAAGCCAAGATAGAGCCT		
StPR-1	StPR1-F:	CGGATGGTGGTTCATTTCATGC	95	AY050221
	StPR1-R:	CATTTAATCCCTTACACATCATAAG		
StPR-5	stPR5-F:	CCCGTTTGACATTGACCTTTG	108	AY737317
	stPR5-R:	CGAATACGGTGGAACATGGA		
StActin	StActin-F	CAAGTCATTACCATCGGTGCTGA	81	Soltu.DM.11G008990
	StActin-R	AGCTGCTTCCATTCCTATCAGT		
M1BEE2	M1BEE2-F	GTTTGCCCGTGCCATGTTTC	120	PGSC0003DMT400043300
	M1BEE2-R	TTATCTGTCCCCCGATGGTG		
M1BF53	M1BF53-F	CCACGAATTTGCCTTATCCAACA	200	PGSC0003DMT400043732
	M1BF53-R	ACAAACGAACTCCAATTTTCTTTTC		
M1A5Z0	M1A5Z0-F	ACATCATTGAGGAGTACTGGCAT	192	PGSC0003DMT400015396
	M1A5Z0-R	GATTCTTGAGTTAACGAACGTGT		
M1BZB1	M1BZB1-F	CCGTCGGGGAACTGGTAAAA	192	PGSC0003DMT400056337
	M1BZB1-B	CTGTCGGGCTCCAGTAAGTC		
